# Synthetic Strategies, Reactivity and Applications of 1,5-Naphthyridines

**DOI:** 10.3390/molecules25143252

**Published:** 2020-07-16

**Authors:** Maria Fuertes, Carme Masdeu, Endika Martin-Encinas, Asier Selas, Gloria Rubiales, Francisco Palacios, Concepcion Alonso

**Affiliations:** Departamento de Química Orgánica I, Facultad de Farmacia, Universidad del País Vasco/Euskal Herriko Unibertsitatea (UPV/EHU), Paseo de la Universidad 7, 01006 Vitoria-Gasteiz, Spain; maria.fuertes@ehu.eus (M.F.); carmen.masdeu@ehu.eus (C.M.); endika_690@hotmail.com (E.M.-E.); asier.selas@ehu.eus (A.S.); gloria.rubiales@ehu.eus (G.R.)

**Keywords:** 1,5-naphthyridines, heterocycle synthesis, metal complexes, biological activity

## Abstract

This review covers the synthesis and reactivity of 1,5-naphthyridine derivatives published in the last 18 years. These heterocycles present a significant importance in the field of medicinal chemistry because many of them exhibit a great variety of biological activities. First, the published strategies related to the synthesis of 1,5-naphthyridines are presented followed by the reactivity of these compounds with electrophilic or nucleophilic reagents, in oxidations, reductions, cross-coupling reactions, modification of side chains or formation of metal complexes. Finally, some properties and applications of these heterocycles studied during this period are examined.

## 1. Introduction

The focus of this review is on the collection of recent advances in the synthesis, reactivity and applications of the 1,5-naphthyridines that have been published in the last 18 years, excluding fused 1,5-naphthyridine analogues. Several reviews have appeared in the naphthyridine area [1,2,3,4], so the coverage of this review is not designed to overlap with these contributions, and therefore, references are, in general, presented from 2003 to the present.

**Figure 1:**
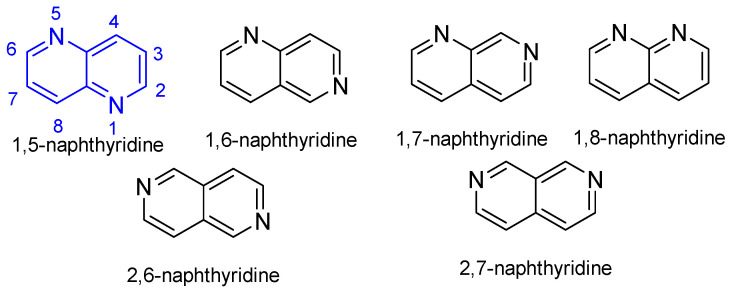
Structures of isomeric naphthyridines.

The first derivative of the cyclic naphthyridine system (a 1,8-naphthyridine) was obtained in 1893 by Reissert [5], who proposed this name for the new class of heterocyclic derivatives, since naphthyridine was considered to be the naphthalene analog, containing two fused pyridine rings with different mutual arrangements of nitrogen atoms. As indicated in Figure 1, six isomeric naphthyridines are possible:

Over the years, these ring systems have received various names, such as “pyridopyridines”, “benzodiazines”, “diazadecalins” or by the “aza” system when named as “diazanaphthalene”. Finally, in 1936, these compounds were indexed in Chemical Abstracts as “naphthyridines”.

In 1927 Brobansky and Sucharda reported the synthesis of the first representatives of unsubstituted naphthyridines, precisely 1,5-naphthyridines, by adapting the Skraup quinoline synthesis to 3-aminopyridine [6]. Since then, several researchers have published their work on the chemistry of 1,5-naphthyridines. From the year 2000, there are over 600 published papers related only to 1,5-naphthyridine, 400 of them as patents. 

The great interest in 1,5-naphthyridines is caused by the wide variety of applications. Among them, biological activities as for example antiproliferative, antibacterial, antiparasitic, antiviral and anti-inflammatory activities could be highlighted. They find application in the cardiovascular, central nervous system and hormonal diseases. In addition, chemical applications as ligands in analytical chemistry or hydrogen acceptors and as organic light-emitting diodes (OLEDs), sensors, semiconductors, solar cells, among others have been considered.

This paper, first, summarizes the work published from 2003, involving the synthesis of 1,5-naphthyridines. Following that that, the reactivity of the compounds giving rise to substituted 1,5-naphthyridines, including several metal complexes, will then be presented. Finally, some properties and applications of these heterocycles studied during this period are reviewed. 

## 2. Synthesis of 1,5-Naphthyridines

Synthetic methods such as cyclization (including Skraup and Friedländer reactions among others), inter- and intramolecular cycloaddition processes (including Povarov reaction, [3+2] cycloadditions, etc.) and other reactions like cross coupling reactions were used to prepare 1,5-naphthyridines. Many of these methods have been well described in previously published reviews [4].

### 2.1. Synthesis of 1,5-Naphthyridines by Cyclization Reactions

**Scheme 1:**
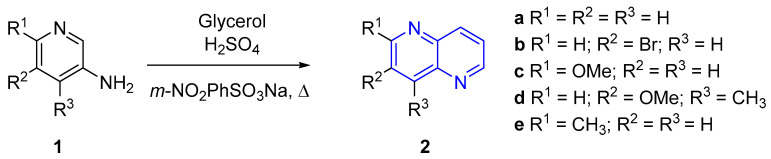
Skraup synthesis of 1,5-naphthyridines.

The simplest Skraup reaction has been used for the synthesis of 1,5-naphthyridine derivatives from substituted 3-aminopyridine compounds **1** and glycerol using different catalyst such as iodine, NaNO_2_, KI, KIO_3_, MnO_2_ or KMnO_4_. Good results were obtained when iodine was used as catalyst in a mixture of dioxane/water (1/1), which is cheap, easy handling and can be recovered and reused efficiently up to five times [7]. Likewise, *m*-NO_2_PhSO_3_Na was used as an oxidant for the preparation of 1,5-naphthyridines **2** (Scheme 1), which displayed a higher yield (45–50%) and a better reproducibility than I_2_ [8]. Additionally, the 3-bromo-1,5-naphthyridine **2b** [9], was reached through a modified Skraup reaction using *m*-NO_2_PhSO_3_Na [10]. 2-Methyl- [11] and 3-methoxy-4-methyl-1,5-naphthyridines **2c**–**2e** [12,13,14] (Scheme 1) were also reported.

**Scheme 2:**
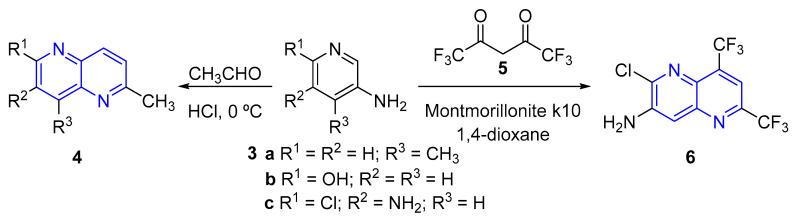
Preparation of 1,5-naphthyridines from 3-aminopyridines and carbonyl derivatives.

2,8-Dimethyl-1,5-naphthyridine **4a** was obtained when 3-amino 4-methyl pyridine **3a** (R^1^ = R^2^ = H, R^3^ = Me) reacted with acetaldehyde (Scheme 2). In the same way, starting from 6-methoxy-3-aminopyridine, 2-hydroxy-6-methyl-1,5-naphthyridine **4b** (R^1^ = OH, R^2^ = R^3^ = H) was prepared [15]. In the same context, the reaction of 2-chloro-3,5-diaminopyridine **3c** (R^1^ = Cl, R^2^ = NH_2_, R^3^ = H) in the presence of hexafluoroacetylacetone **5** and montmorillonite k10 as catalyst, yielded the corresponding 1,5-naphthyridine **6** as a yellowish solid (Scheme 2) [16].

**Scheme 3:**
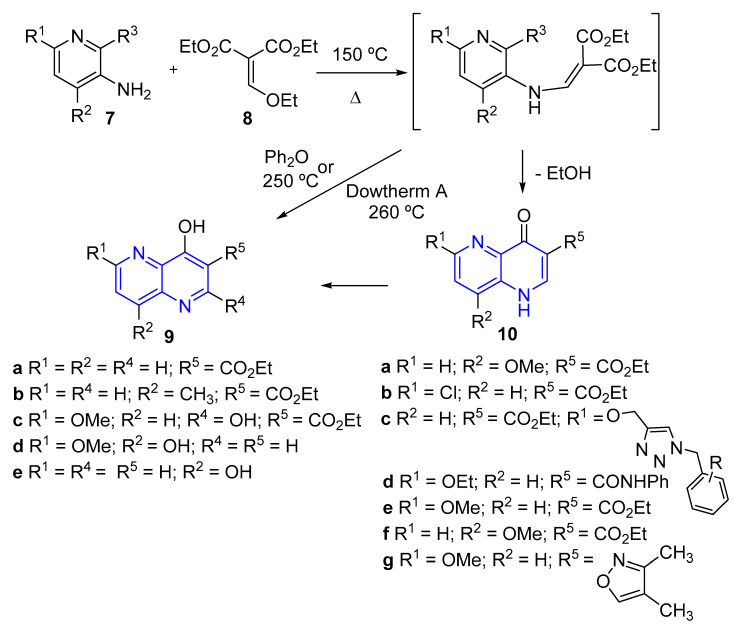
Gould-Jacobs reaction for the preparation of 1,5-naphthyridine derivatives.

Gould-Jacobs reaction, reported by Brown and Dewar in 1978 [17], between 3-aminopyridine **7** and diethyl methylenemalonate **8** followed by a thermal cyclization afforded the 1,5-naphthyridine **9a** (Scheme 3) [18]. 4-Hydroxy-1,5-naphthyridine **9b** was also prepared by a condensation reaction at 150 °C followed by a ring cyclization to yield the 1,5-naphthyridine skeleton (Scheme 3) [19]. Similarly, 1,5-naphthyridine derivative **10a** has been obtained [20]. Moreover, in a recent study, the same reaction was applied for the synthesis of 1,5-naphthyridine **10b** (Scheme 3). However, in this particular case chlorobenzene was the solvent of choice to carry out the cyclization [21].

This reaction was used to synthesize 1,5-naphthyridine derivative **10c** [22] and **9d** by the condensation followed by a cyclization and decarboxylation [11] (Scheme 3). As well as for 6-ethoxy-4-oxo-1,4-dihydro-1,5-naphthyridine-3-carboxylic acid benzylamide **10d** (Scheme 3), including a multikilogram scale synthesis of this compound [23]. This methodology was also applied for the preparation of biologically active compounds [24,25]. The same strategy [26] was used to find new methodologies for the late-stage introduction of fluorine into advanced hydroxy-naphthyridine, leading the 1,5-naphthyridone rings in compounds **10e**–**10f** (Scheme 3). Similarly, this process was used for the development of 1,5-naphthyridine **9e** (Scheme 3). In this case, formation of the second pyridine ring and the subsequent decarboxylation step were performed by prolonged refluxing in concentrated hydrobromic acid, rather than using quinoline and vacuum-thermal decarboxylation, which sped up and eased the synthesis [27].

**Scheme 4:**
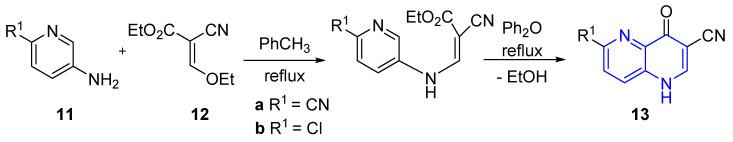
Modified Gould-Jacobs reaction for the preparation of 1,5-naphthyridinecarbonitriles.

Wei et al., following their studies to develop naphthyridine-based Eu(III) complexes, reported an extension of the method by using methyl-2-cyano-3-methoxybut-2-enoate **12** instead of ethoxymethylene malonate in order to obtain the corresponding 1,5-naphthyridinecarbonitrile derivatives **13a** and **13b** (Scheme 4) [28,29].

**Scheme 5:**
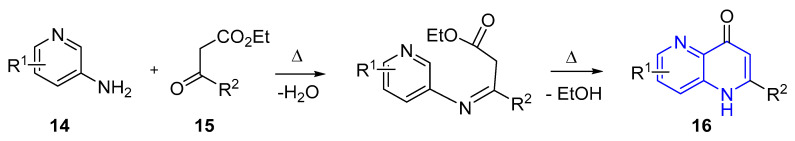
Conrad-Limpach reaction with β-ketoesters for the preparation of 1,5-naphthyridinones.

Another of the earliest methods to obtain naphthyridin-4-ones **16** (Scheme 5), which often isomerize (or aromatize) to 4-hydroxyquinolines was the Conrad and Limpach reaction [4]. The reaction consists a thermal condensation of primary aromatic amines **14** with the carbonyl group of *β*-ketoesters **15** followed by the cyclization of the corresponding Schiff base intermediates [30].

**Scheme 6:**
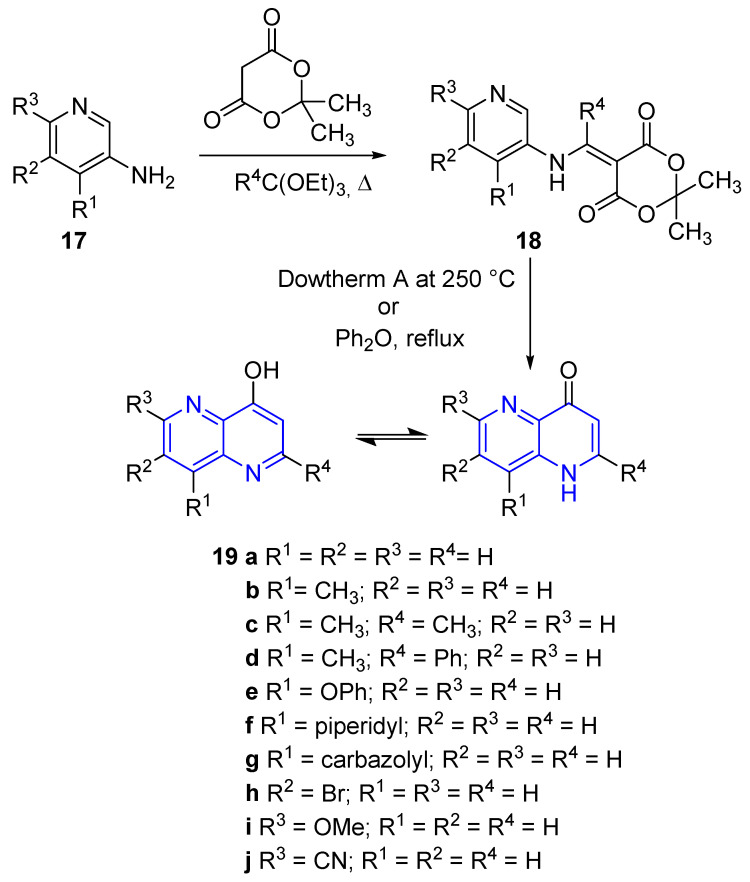
Conrad-Limpach reaction with Meldrum’s acid for the preparation of 1,5-naphthyridinones.

This reaction was later extended for the use of Meldrum’s acid instead of *β*-ketoesters to afford 4-hydroxynaphthyridines **19** (Scheme 6). Therefore, the synthesis of the 8-hydroxy-1,5-naphthyridines **19a**–**d** (Scheme 6) has been carried out by reaction between Meldrum’s acid and 3-aminopyridine derivatives **17** after condensation [31]. Initially, it furnished the corresponding enamine **18** in good yields. Ring formation was accomplished by heat-assisted intramolecular cyclization in Dowtherm A or diphenyl ether and the subsequent decarboxylation led to the formation of 8-hydroxy-1,5-naphthyridine **19h** (Scheme 6) [32]. Similarly, in the synthesis of some heteroleptic platinum complexes (FPtXND) bearing 4-hydroxy-1,5-naphthyridine the use of Meldrum’s acid was also the method of choice using substituted triethyl orthoformate to introduce a substituent at the 8 position of the ring (**19a**,**c**,**e**–**g**, Scheme 6) [33].

Some 1,5-naphthyridine intermediates for fluorination assays [26] were obtained through a cyclization method involving aminopyridine with Meldrum’s acid in triethylformate at 100 °C, and subsequently heating in Dowtherm A at 250 °C (compound **19a**, Scheme 6) [34]. Likewise, this process was used for the preparation of the ligand 8-hydroxy-1,5-naphthyridine-2-carboxylic acid (H2L1) [28], a 2,8-disubstituted-1,5-naphthyridine [35] and compounds **19i**,**j** (Scheme 6) [27].

**Scheme 7:**
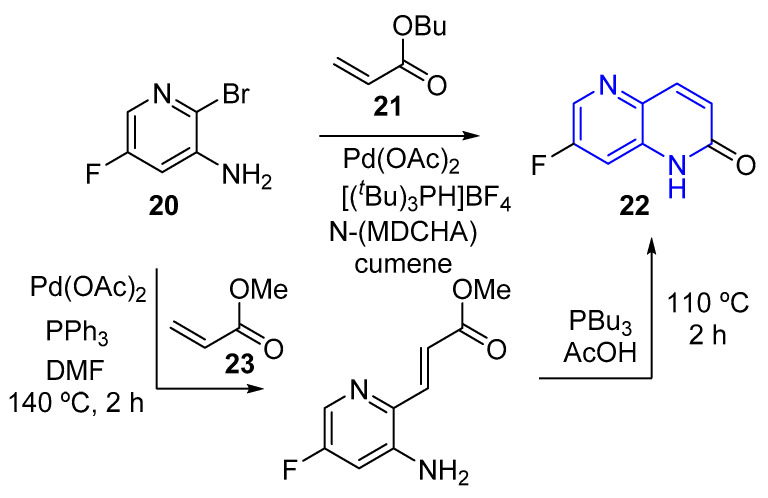
Palladium mediated synthesis of 1,5-naphtyridinones from 3-aminopyridines and acrylates.

Another synthetic approach to lead 1,5-naphthyridine consists on cross-coupling reactions followed by cyclization reactions. In this context, the Heck reaction of the corresponding aminopyridine **20** with Pd(OAc)_2_, tri-*tert*-butylphosphonium tetrafluoroborate, *N*,*N*-dicyclohexylmethylamine (N-MDCHA) in cumene at 150 °C overnight yielded an intermediate which evolves by cyclization to 1,5-naphthyridine **22** [36]. In the same background, a novel electron-accepting bis-lactam building block structurally based on 1,5-dihydro-1,5-naphthyridine-2,6-dione was achieved following the same procedure above mentioned [37]. Similarly, a 1,5-naphthyridine derivative **22** was prepared from 2-bromo-6-fluoropyridin-3-amine **20** using Heck reaction of the substituted pyridine with methyl acrylate **23** in good yield. Cyclization was achieved using PBu_3_ in AcOH in excellent yields (Scheme 7) [38]. 

**Scheme 8:**
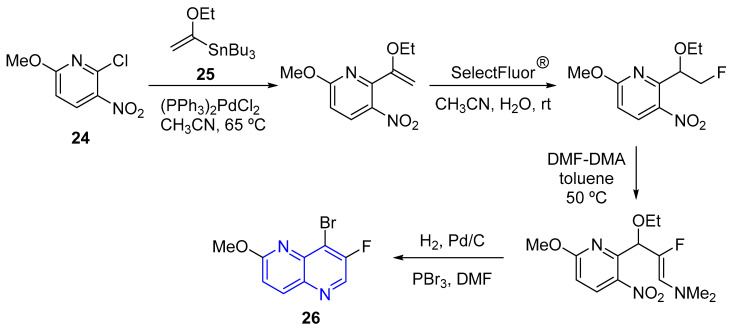
Synthesis of 1,5-naphthyridines based on Stille cross-coupling reaction.

A Stille cross-coupling reaction was also used to reach the 1,5-naphthyridine ring. Thus, chloronitropyridine **24** underwent reaction with tributyl(1-ethoxyvinyl)tin **25**, which after fluorination and subsequent condensation with DMF·dimethylacetal and followed by the nitro reduction−deoxobromination sequence, resulted in the 1,5-naphthyridine ring **26** in good yield (Scheme 8) [26].

**Scheme 9:**
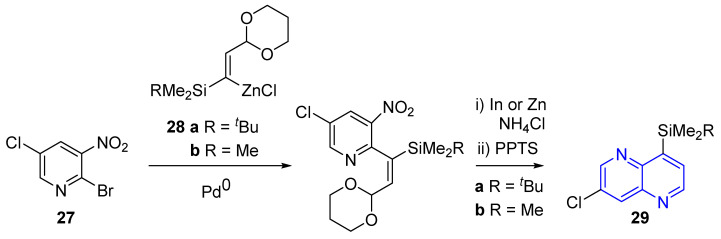
Knochel approach for the synthesis of 1,5-naphthyridines.

In another approach, Knochel et al. described the preparation of new conjunctive alkenyl-metal reagents **28** (Li, Mg, Zn), bearing a latent aldehyde function and a silyl group facilitating further functionalization, which may be converted into various carbon–carbon bonds. These versatile building blocks allow the synthesis of various classes of important heterocycles, specially the 1,5-naphthyridine. Using bromonitro pyridine **27** as coupling reagents provided a short access to the valuable 1,5-naphthyridines **29a**,**b** in 60% yield when pyridinium *p*-toluenesulfonate (PPTS) was used (Scheme 9) [39].

**Scheme 10:**
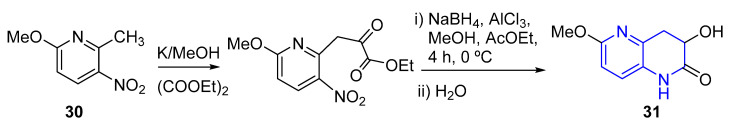
Synthesis of 3-hydroxy-1,5-naphthyridinone.

Through an alternative cyclization process, the core of 1,5-naphthyridine **31** was accomplished by treatment of the corresponding pyridine **30** with diethyl oxalate, subsequent reduction of the nitro and carbonyl functions with aluminium borohydride, followed by concomitant lactamization (Scheme 10) [40].

**Scheme 11:**
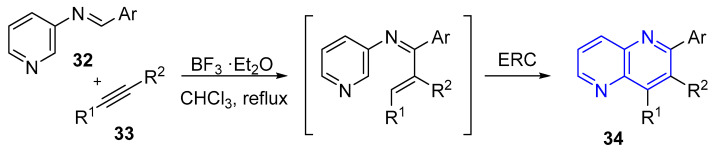
[4+2] cycloaddition reaction of imines and acetylenes for the synthesis of 1,5-naphthyridines.

On the other hand, our group described the synthesis of 1,5-naphthyridines **34** through an electrocyclic ring closure by reaction between *N*-(3-pyridyl)aldimines **32** and alkynes **33** (Scheme 11) [41]. The reaction was performed in the presence of a Lewis acid, such as BF_3_·Et_2_O. Moreover, the combined experimental and computational investigations between *N*-(3-pyridyl)aldimines **32** and acetylenes **33** with a Lewis acid may suggest a stepwise [4+2]-cycloaddition mechanism. The presence of nitrogen in the pyridine ring deactivates the electrophilic substitution with respect to the benzene, yielding the formation of a 3-azatriene whose electrocyclic ring closure (ERC) may give the corresponding heterocyclic intermediates, followed by prototropic tautomerization and subsequent aromatization, to afford naphthyridines **34** (Scheme 11).

### 2.2. Synthesis of 1,5-Naphthyridines by Cycloaddition Reactions

**Scheme 12:**
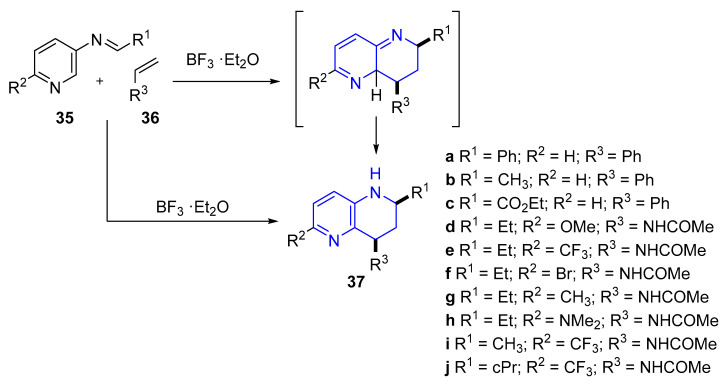
Aza-Diels-Alder reaction for the synthesis of 1,2,3,4-tetrahydro-1,5-naphthyridines.

First, the aza-Diels-Alder reaction (Povarov reaction) [42] activated by Lewis acid has been studied theoretically and experimentally for the synthesis of 1,2,3,4-tetrahydro-1,5-naphthyridine derivatives **37a** and **37b** (Scheme 12), which can be obtained through *endo* intermediates in a regio- and stereoselective manner [43]. In the same way, glyoxalate derived 1,5-naphthyridine **37c** was prepared through a [4+2] cycloaddition process via *endo* transition states [44].

Following this strategy, a series of 4-phenyl-1,5-naphthyridine derivatives were synthesized between imines **35**, prepared from 3-aminopyridines and aldehydes, and styrenes **36** as olefins (Scheme 12). The cycloaddition proceeds through *endo* transition states to afford tetrahydro-1,5-naphthyridines **37a**–**c** with the control of two stereocenters. Subsequent aromatization afforded the corresponding 4-phenyl-1,5-naphthyridines [45,46]. 1,5-Naphthyridine derivatives **37d**–**j** were prepared by a modification of a Diels-Alder reaction between aldimines **35**, derived from 3-aminopyridine and vinyl acetamide **36** (R^3^ = NHCOMe) to give the corresponding racemic mixture of the *cis* isomers (Scheme 12) [47].

## 3. Reactivity of 1,5-Naphthyridines

The reactivity pattern of 1,5-naphthyridines shows similarities with quinolines [4]. Therefore, electrophilic substitution including *N*-alkylation reactions, nucleophilic substitution, reduction, oxidation, metalation and cross coupling as well as the modifications in the side chain, metal complex formation and polymerization among other reactions should be considered.

### 3.1. Reactions with Electrophilic Reagents

Reactions in this category applied for pyridine, quinoline and isoquinoline, which involve the donation of the nitrogen lone pair to electrophiles, also occur with the 1,5-naphthyridines [4].

#### 3.1.1. *N*-alkylation, *N*-acylation and *N*-tosylation

**Scheme 13:**
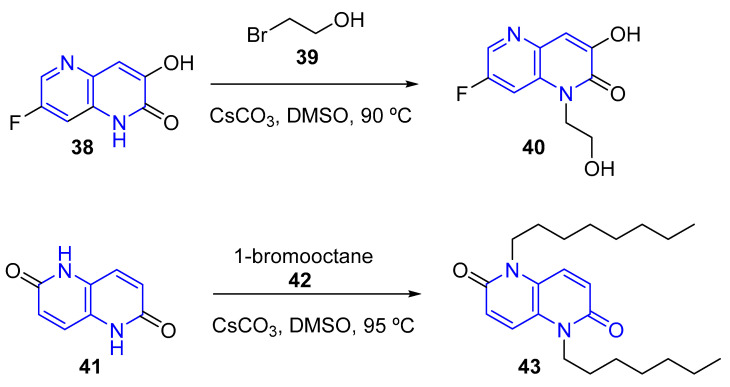
*N*-alkylation reaction of 1,5-naphthyridinones.

Alkyl halides react readily with 1,5-naphthyridines to furnish the corresponding *N*-alkylsubstituted 1,5-naphthyridines **40** and **43** (Scheme 13) through quaternary salts intermediates, which undergo base-induced hydrogen halide elimination. In this sense, the alkylation with 2-bromoethanol **39** in the presence of cesium carbonate as a base provided *N*-alkylated 1,5-naphthyridine **40** [36]. Similarly, the same procedure was used to dialkylate 1,5-naphthyridine-2,6-dione **41** with 1-bromooctane **42** to give derivative **43** [37]. 

**Scheme 14:**
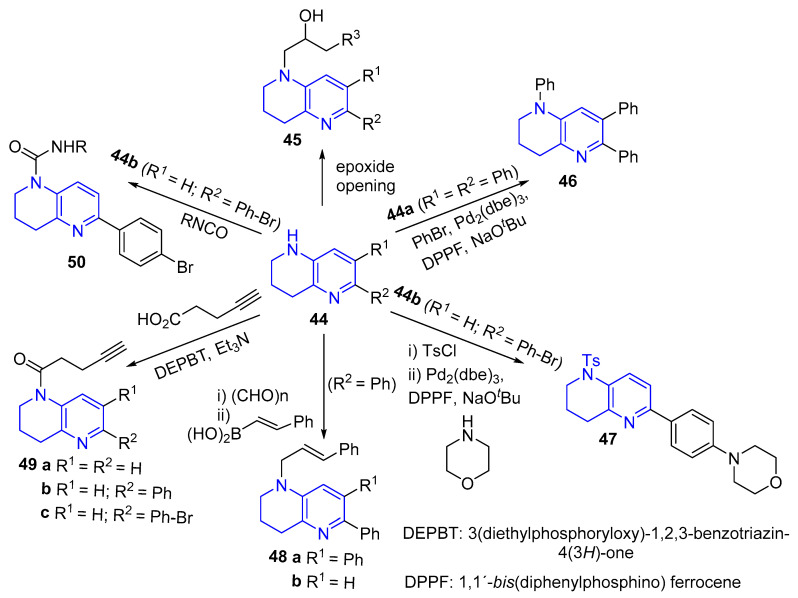
*N*-functionalization reactions of 1,5-naphthyridines.

Snyder et al. studied the use of tetrahydro-1,5-naphthyridines as scaffolds to construct a compound library (Scheme 14). Through the reactivity of the N1 nitrogen as a nucleophile with a wide range of electrophilic reagents such as isocyanates, homopropargylic acid, tosyl halides, epoxides, and in cross-coupling processes, the pyridine ring was modified to prepare a 24 membered library [48].

#### 3.1.2. Halogen Substitution at Carbon

(a) Fluorination

**Scheme 15:**
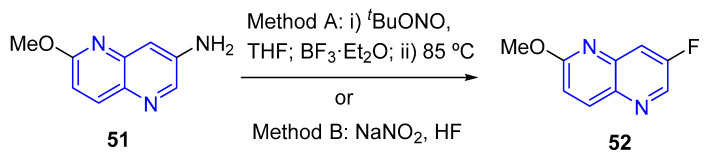
Diazotation−fluorodediazoniation sequence for the synthesis of fluorinated 1,5-naphthyridine.

Diazotation−fluorodediazoniation is a useful method for the regioselective introduction of fluorine into aromatic rings. However, two major risks have to be assessed when scaling up this process. First, the thermal instability of the diazonium salt might lead to premature loss of nitrogen (sudden pressure build up) so prolonged storage in tanks or transfer through lines is dangerous. In addition, some diazonium salts are friction sensitive and explode after subjection to mechanical stress. Second, the fluorodediazoniation, the release of nitrogen, is performed at elevated temperature and the exothermic reaction, gas evolution, foaming, and accumulation have to be controlled. To perform the reaction, firstly, the diazonium tetrafluoroborate was prepared with tert-butylnitrite and BF_3_·Et_2_O in tetrahydrofuran (THF). The diazonium salt was then dosed in portions to heptane at 85 °C to trigger the fluorodediazoniation to the fluorinated 1,5-naphthyridine **52** in good yields (see method A, Scheme 15). In an effort to find new methodologies for the late-stage introduction of fluorine into advanced intermediates, two new strategies have been described. The introduction of the fluorine was accomplished employing lower-cost fluorination reagents HF or F_2_ gas, both being routinely used on large scale by specialists in the fine chemicals industry. Then, the one-pot diazotation−fluorodediazoniation protocol of 1,5-naphthyridine **51** with NaNO_2_ in HF or pyridine/HF was an attractive option for scale-up as it afforded the fluorinated compound **52** in high yield (see method B, Scheme 15).

**Scheme 16:**
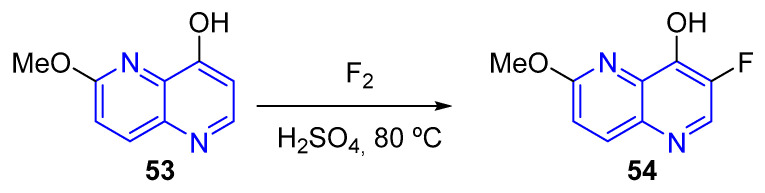
ortho-Fluorination of hydroxy-1,5-naphthyridines with fluor gas.

An additional alternative was offered by the surprisingly selective ortho-fluorination of hydroxy-naphthyridines **53** with F_2_ gas, without the ubiquitous tarry byproducts reported for such transformations (Scheme 16). A critical parameter was the mass transfer, hence the dispersion of F_2_ gas in the mixture, and the work-up. The preferred solvent was concentrated H_2_SO_4_. It might be worthwhile to further investigate other solvents (ideally acidic solvents that can be removed by distillation) with additives (e.g., HBF_4_) that will facilitate the work-up of fluorohydroxynaphthyridine **54** [26].

(b) Chlorination

Incorporation of chlorine in 1,5-naphthyridine derivatives may be favored by means of peracids involving the initial formation of the corresponding mono *N*-oxide derivatives (vide infra, Section 3.3.1.b).

(c) Bromination

**Scheme 17:**
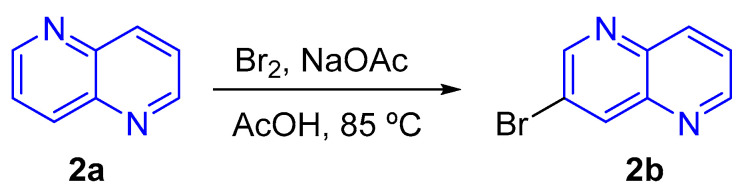
Bromination of 1,5-naphthyridine.

Bromination of 1,5-naphthyridine **2a** are often carried out to obtain valuable intermediates for further functionalization. As an example, bromination with bromine in acetic acid (Scheme 17) was used in the synthesis of 1,5-naphthyridine derivative **2b** (previously prepared, vide supra, Scheme 1) [9].

**Scheme 18:**
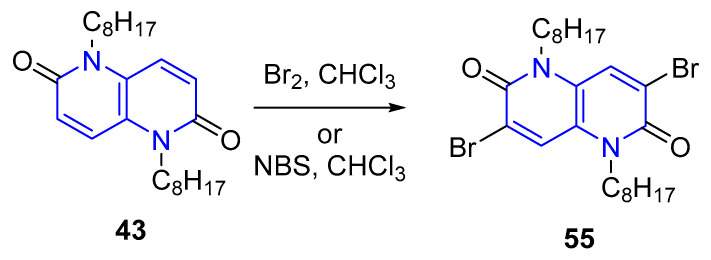
Dibromination of 1,5-naphthyridine-2,6-diones.

Dibromated compound was obtained when 1,5-dialkyl-1,5-naphthyridine-2,6-dione **43** (previously prepared, vide supra, Scheme 13) was treated with bromine to obtain 3,7-dibromo-1,5-dioctyl-1,5-naphthyridine-2,6-dione **55** (Scheme 18) as a suitable intermediate to perform next coupling reactions [37]. The same group, in further studies, used *N*-bromosuccinimide (NBS), as a bromine source, to synthesize more conjugated polymers [49].

### 3.2. Reactions with Nucleophilic Reagents

#### 3.2.1. Perfluoroalkylation

**Scheme 19:**
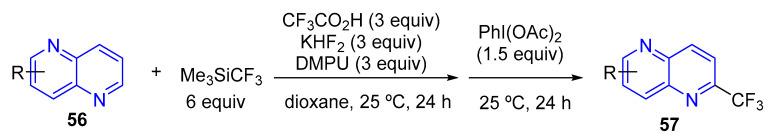
Trifluoromethylation of 1,5-naphthyridines.

Due to the importance of introducing perfluoroalkyl groups in order to improve the functions of organic molecules [50], Kuninobu et al. developed a regioselective direct C−H trifluoromethylation, pentafluoroethylation, and heptafluoropropylation of 1,5-naphthyridines, using perfluoroalkyltrimethylsilane, trifluoroacetic acid, KHF_2_, and 1,3-dimethyl-3,4,5,6-tetrahydro-2(1*H*)-pyrimidinone (DMPU). The key step of the reaction consists of dual activation of both *N*-heteroaromatic substrates and trifluoromethyltrimethylsilane by hydrogen fluoride (HF) through the formation of a six-membered transition state. In this way, 1,5-naphthyridine **56** was selectively trifluoromethylated at C-2 in 32% yield (compound **57**, Scheme 19). It is important to highlight that the reaction proceeded with high functional group tolerance, including an oxidation-sensitive formyl group, which might not be tolerated under the previous conditions [51].

**Scheme 20:**
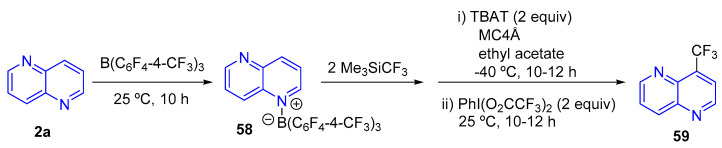
Trifluoromethylation of 1,5-naphthyridines mediated by boranes.

However, the first four-position-selective C−H perfluoroalkylation and perfluoroarylation of six-membered heteroaromatic compounds were achieved using nucleophilic perfluoroalkylated and perfluoroarylated reagents promoted by bulky borane Lewis acid. The regioselectivity was controlled by activating the heteroaromatic rings, while sterically hindering the two-position, with a bulky borane Lewis acid. The reaction proceeded to give 1,5-naphthyridine **59** in good yield (59%), even in gram scale, and by a sequential reaction involving tetrabutylammonium difluorotriphenylsilicate (TBAT) followed by the treatment with [bis(trifluoroacetoxy)iodo]benzene without isolating the intermediates (Scheme 20). This reaction could be applied to late stage trifluoromethylation of a bioactive compound [52].

#### 3.2.2. Cyanation

**Scheme 21:**
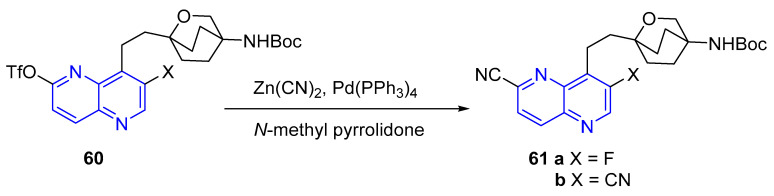
Metal catalyzed cyanation of 1,5-naphthyridines.

The incorporation of a cyano group into the heterocyclic ring of 1,5-naphthyridine (Scheme 21) was achieved by the formation of mono *N*-oxide or di-*N*-oxide derivatives (vide infra, Section 3.3.1). However, nucleophilic displacement of the triflate with cyano group in compound **60** produced the corresponding C-2 cyano derivatives **61** [53].

#### 3.2.3. Direct Amination

**Scheme 22:**
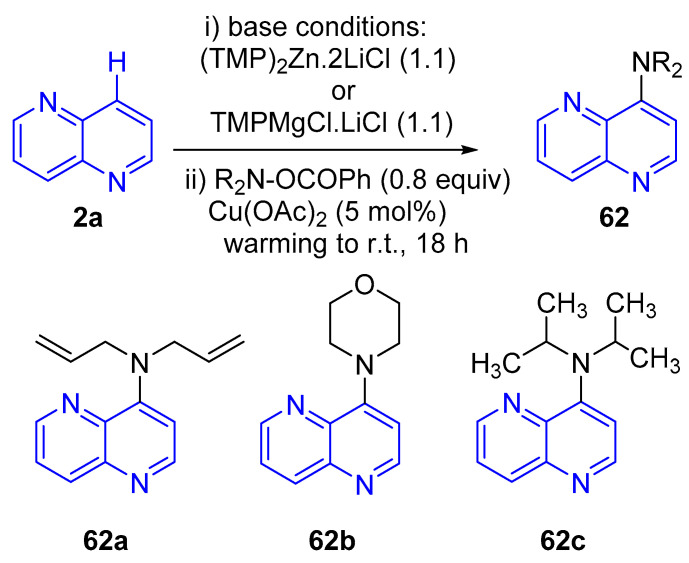
Deprotometalation-amination reaction of 1,5-naphthyridines.

Besides direct introduction of an amino group by a Chichibabin reaction [4], functionalization at C-4 can be achieved by a direct deprotometalation-amination reaction (Scheme 22). This methodology appears complementary to access to several 4-amino substituted 1,5-naphthyridines **62a**–**c** [54].

**Scheme 23:**
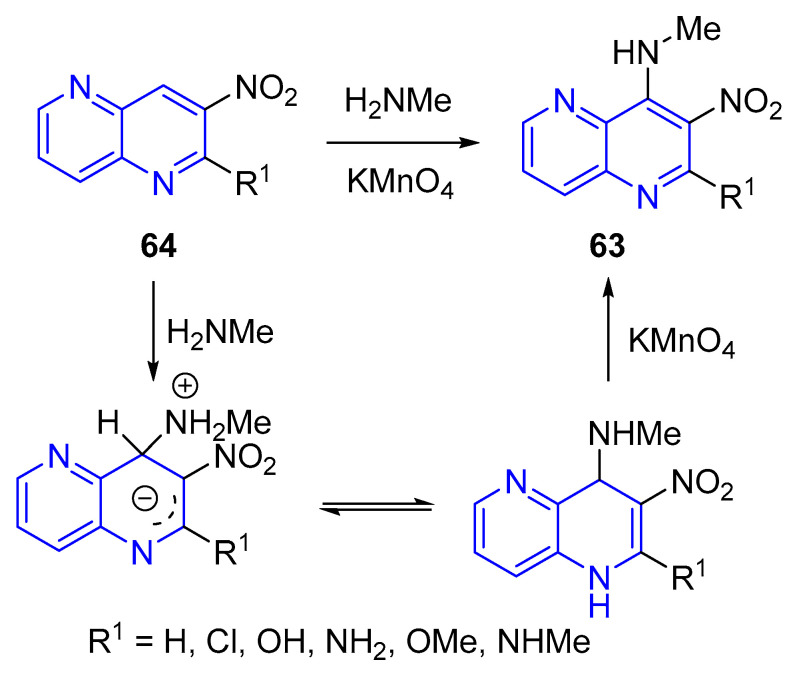
Amination of nitro-1,5-naphthyridines.

Grzegozek et al. reported the synthesis of 4-methylamino-3-nitro-1,5-naphthyridine **63** (Scheme 23) by a methylamination reaction of some 2-substituted-3-nitro-1,5-naphthyridine derivatives **64** with a solution of potassium permanganate in liquid methylamine [55].

#### 3.2.4. Phosphorylation

**Scheme 24:**
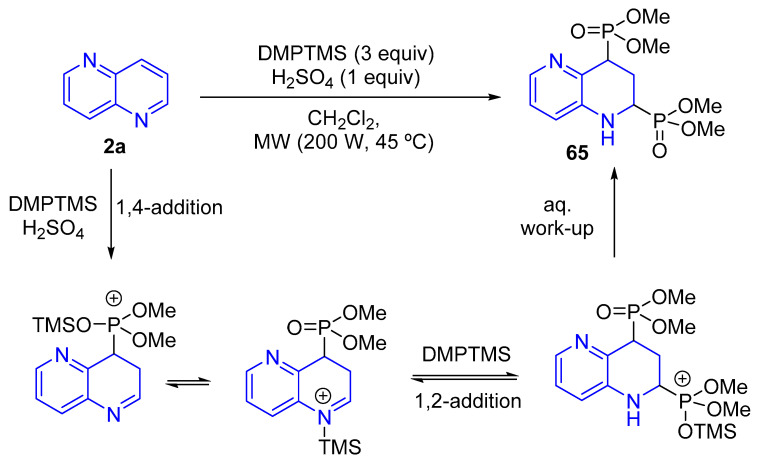
Diphosphorylation of 1,5-naphthyridine.

Aminophosphonates and their corresponding aminophosphonic acids are known as amino acid mimetics and, therefore, affect the physiological activity of the cell [56,57]. The synthesis of 3-phosphonylated aminophosphonates from α*,*β-unsaturated imines through tandem 1,4-1,2-phosphite addition was first accomplished by the use of both silylated dialkyl phosphite and trialkyl phosphates [58]. Diphosphonylated diazaheterocyclic compounds **65** were synthesized in a single step reaction by using dimethyl trimethylsilyl phosphite (DMPTMS) under acidic conditions (Scheme 24) in dry dichloromethane. The reaction of DMPTMS with 1,5-naphthyridine **2a** (previously prepared, vide supra, Scheme 1) yielded the corresponding diphosphonylated product **65** through a tandem 1,4-1,2 addition under microwave conditions. Reactions under reflux and microwave conditions were compared. 1,5-Naphthyridine derived substrates are less reactive than previously investigated quinolines [59].

**Scheme 25:**
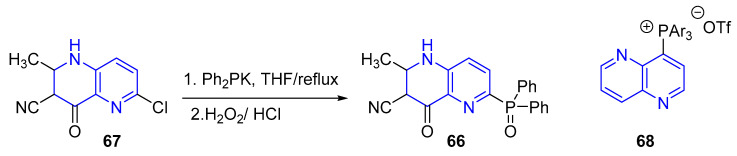
Preparation of 1,5-naphthyridine phosphanoxides and phosphonium salts from chloro-1,5-naphthyridines.

1,5-Naphthyridines containing a diphenylphosphoryl group **66** have been used for the synthesis of tridentate europium(III) complexes and were prepared by a nucleophilic substitution reaction between potassium diphenylphosphanide and compound **67** followed by hydrogen peroxide oxidation (Scheme 25) [29]. While, phosphonium salts **68** were prepared by the reaction of 1,5-naphthyridine **2a** with triarylphosphines and triflic anhydride in the presence of 1,8-diazabicycloundec-7-ene (DBU) [60].

#### 3.2.5. Halogenation of hydroxy-1,5-naphthyridine and/or 1,5-naphthyridinones

**Scheme 26:**
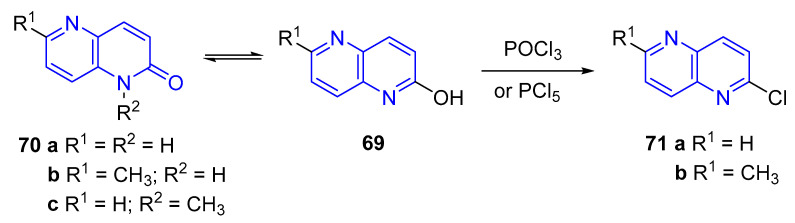
Chlorination of 1,5-naphthyridine-2(1*H*)-ones.

The conversion of the carbonyl group of 1,5-naphthyridinones into a leaving group has a very important place in the chemistry of these compounds, the most frequently encountered examples involving reaction with phosphoryl halide and/or phosphorus pentahalide leading to halo-naphthyridines, through an assumed dihalophosphate intermediate. Given that the halide group is a good leaving group, halo-1,5-naphthyridine derivatives are very interesting intermediates for the introduction of nucleophilic reagents in the 1,5-naphthyridine ring and may be prepared by halogenation of hydroxyl-1,5-naphthyridines. For example, the 1,5-naphthyridine derivatives were prepared [61] and converted into the corresponding 2-chloro derivative **71a** using phosphorus oxychloride with 1,5-naphthyridine-2(1*H*)-one **70a** (Scheme 26) [62], and has been used for synthesis of 1,5-naphthyridine functionalized indenyl and cyclopentadienyl ligands. The same strategy has been used for the preparation of chloro-1,5-naphthyridine derivative **71b** (Scheme 26) [15]. Chlorination of the carbonyl derivative **70c** using PCl_5_ or POCl_3_ afforded the corresponding 2-chloro-1,5-naphthyridine **71a** (Scheme 26) [63], used for the dimerization of the naphthyridine to obtain 2,2’-bi-1,5-naphthyridine dimer.

**Scheme 27:**
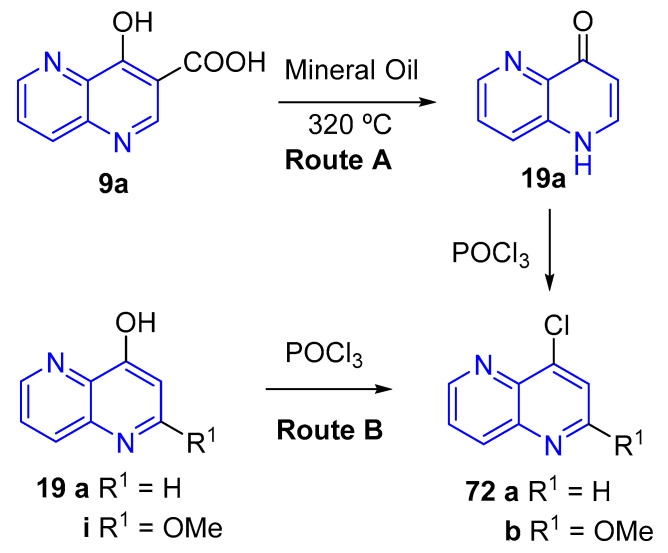
Chlorination of 1,5-naphthyridine-4(1*H*)-ones.

On the other hand, chlorination with POCl_3_ of the 1,5-naphthyridine-4(1*H*)-one **19a** (previously prepared, vide supra, Scheme 6), generated by thermal decarboxylation of 1,5-naphthyridinyl carboxylic acid **9a** (previously prepared, vide supra, Scheme 3) afforded 4-chloro 1,5-naphthyridine **72a** (Scheme 27, route A) [18]. Likewise, Lewin et al. reported a new method for the preparation of SB-334867 by a direct halogenation of commercially available 4-hydroxy-1,5-naphthyridine **19a** and **19i** (previously prepared, vide supra, Scheme 6) to afford the compounds **72a**,**b** (Scheme 27, route B). This process can also be extended to the formation of 5,8-dichloro-naphthyridine from 5,8-hydroxy-1,5-naphthyridine [64].

**Scheme 28:**
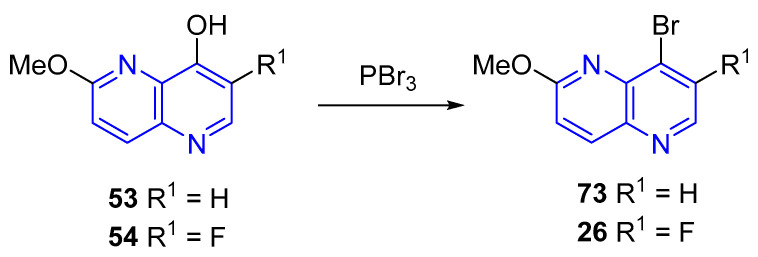
Bromination of 4-hydroxy-1,5-naphthyridines.

Similarly, 1,5-naphthyridine bromide derivatives **73** and **26** (previously prepared, vide supra, Scheme 8) were prepared by refluxing the corresponding hydroxyl derivatives **53**–**54** in neat phosphorus tribromide (Scheme 28) [65], while 4,8-dibromo 1,5-naphthyridine has been also synthesized by bromation with POBr_3_ from 1,5-naphthyridine-4,8(1*H*,5*H*)-dione [20].

**Figure 2:**
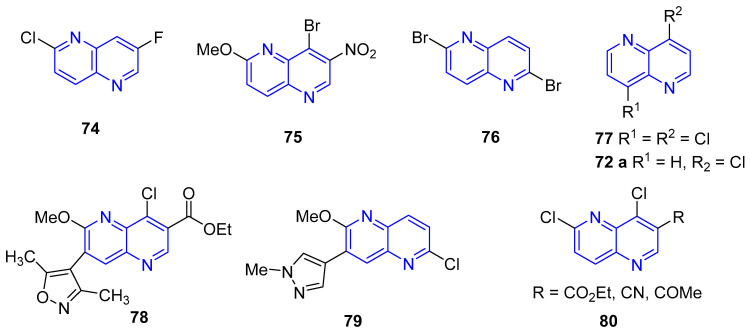
Compounds obtained by halogenation of hydroxy-1,5-naphthyridine derivatives.

Other halogenation reactions were carried out in the preparation of fluorine intermediates (compounds **74** and **75**, Figure 2) [26]. In this sense, the reaction was applied for the synthesis of 1,5-naphthyridine-based polymers, new functional materials for electronics (compound **76**, Figure 2) [40], active naphthyridine derivatives for the development of novel anti-Ebola virus pharmacophore (compound **77**, Figure 2) [27], a series of naphthyridine derivatives as bromo domain inibitors (compound **72a**, Figure 2) [24], for the development of some novel 1,5-naphthyridines as c-Met kinase inhibitors (compound **78**, Figure 2) [9], for the synthesis of a 1,5-naphthyridine analogues of a first in class Rpn 11-selective proteasome inhibitor (compound **79**, Figure 2) [34] or for the synthesis of 1,5-naphthyridine-based DYRK1A inhibitors (compound **80**, Figure 2) [21].

#### 3.2.6. Nucleophilic Substitution with Displacement of Good Leaving Groups

(a) Amination

**Scheme 29:**
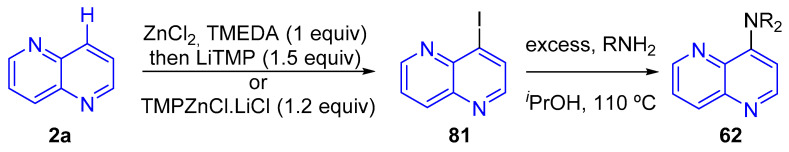
Iodation-amination sequence for the synthesis of 1,5-naphthyridine.

Functionalization at C-4 of 1,5-naphthyridine heterocycle **2a** can be achieved by a direct deprotometalation-amination reaction (vide supra, Scheme 22) or by an indirect amination. The latter method involves a previously deprotometalation-iodolysis substitution reaction to afford the intermediate **81** (Scheme 29), which permitted the access to a variety of 4-amino substituted 1,5-naphthyridines **62** (previously prepared, vide supra, Scheme 22) [54].

**Scheme 30:**
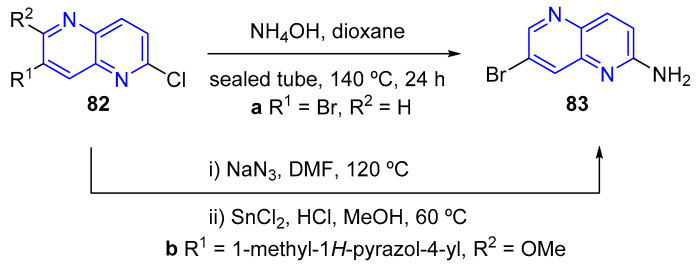
Preparation of 2-amino-1,5-naphthyridine from 2-chloro-1,5-naphthyridine.

Selective amination by substitution of chlorine atom in 1,5-naphthyridine **82** (R^1^ = Br, R^2^ = H), was performed in the presence of ammonium hydroxide in sealed tube at 140 °C to synthesize the corresponding amines **83** (Scheme 30) [10]. Azidation of **82** with NaN_3_ followed by reduction with SnCl_2_, the 1,5-naphthyridine **83** (R^1^ = 1-methyl-1*H*-pyrazol-4-yl, R^2^ = OMe) scaffold was also obtained in 63% (Scheme 30) [9].

**Scheme 31:**
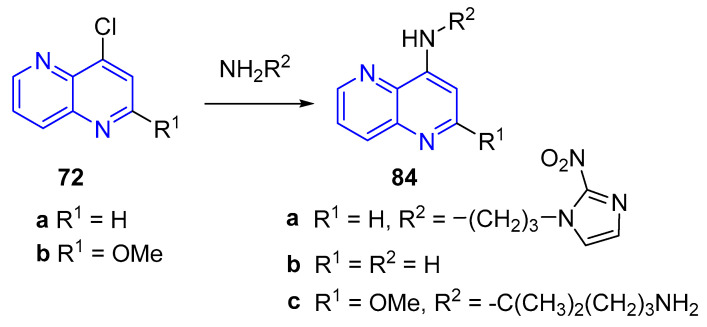
Amination of 4-chloro-1,5-naphthyridines.

Similarly, 4-amino-1,5-naphthyridine **84a** has been prepared by a conversion of the 4-chloro-1,5-naphthyridine **72a** (previously prepared, vide supra, Scheme 27) with 3-(2-nitro-1-imidazolyl)-propylamine [18]. This same methodology was applied by Lewin et al. for the preparation of a intermediate to obtain ligand SB-334867, where by a direct amination of the compound **72a** afforded the corresponding derivative **84b** in good yields (Scheme 31) [64].

**Scheme 32:**
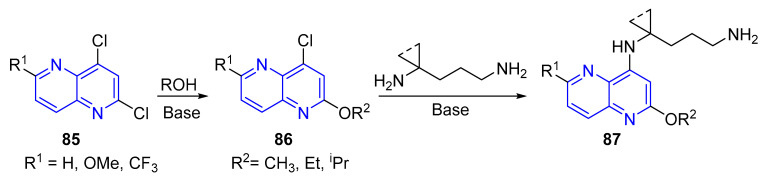
Alkoxylation-amination sequence of dichloronaphthyridine derivatives for the synthesis of alkoxy amino-1,5-naphthyridines.

4-Chloro-1,5-naphthyridine **72b** (previously prepared, vide supra, Scheme 27) was reacted also with 2-(4-amino-4-methylpentyl)-isoindole-1,3-dione after deprotonation with sodium hydride, in a S_N_2 type mode, which is followed by removal of the protective group on the terminal amine to form **84c** (Scheme 31). Whereas, the use of dichloro derivatives **85** led to the formation of functionalized 4-amino-1,5-naphthyridine derivatives **87**, by means of a sequential incorporation of the group followed by the amino moiety (Scheme 32) [11].

**Figure 3:**
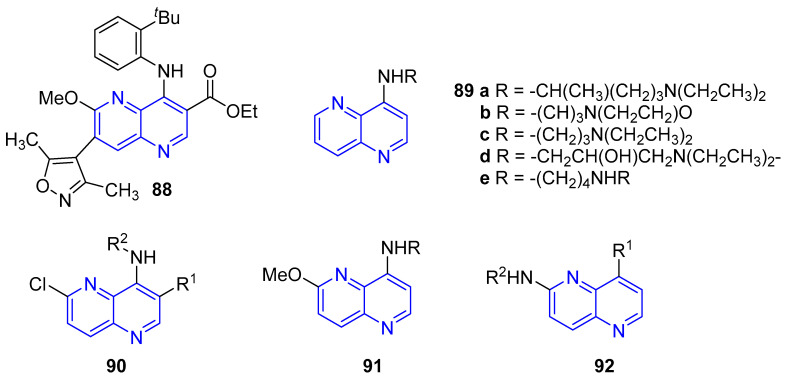
Compounds obtained by amination of halogenated 1,5-naphthyridine derivatives.

In this sense, a series of naphthyridine derivatives **88** were prepared in good yields by chlorination followed by thermal condensation with 2-*tert*-butylaniline (Figure 3) [24]. Recently, the development of a novel anti-Ebola virus pharmacophore, with a 1,5-naphthyridine core, have been reported from chlorinated naphthyridines. These compounds were then subjected to microwave-assisted nucleophilic substitution reactions with appropriate amines. This gave rise to different alkylamino substituted compounds **89** (Figure 3) [27].

A one step synthesis of 1,5-naphthyridine-based DYRK1A inhibitors consisted in the S_N_Ar reaction of the 1,5-naphthyridine core by means of a variety of elaborated amines for introducing amino substituents at the four-position (**90**, Figure 3) [21]. Likewise, a series of 2,8-disubstituted-1,5-naphthyridine analogues were synthesized and evaluated for in vitro antiplasmodial activity, as inhibitors of Plasmodium protein kinases (PKs). The brominated key intermediate was subjected to nucleophilic aromatic substitution using commercially available amines, respectively, in the presence of Cs_2_CO_3_ at 110 °C to give the corresponding derivatives **91** and **92** (Figure 3) [35].

**Scheme 33:**
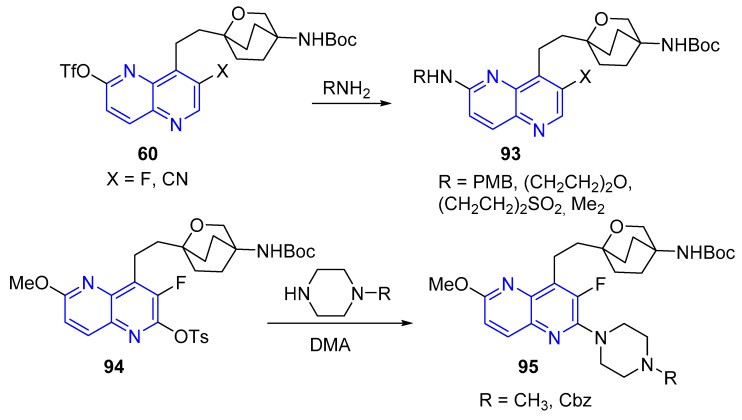
Nucleophilic amination of triflate and tosyl substituted 1,5-naphthyridines.

Some additional nucleophilic amination reactions with replacement of leaving groups other than halogens were also performed. Triflate and tosyl groups were substituted by nucleophilic amines to yield the corresponding functionalized 1,5-naphthyridine compounds **93** and **95** (Scheme 33) [53].

**Scheme 34:**
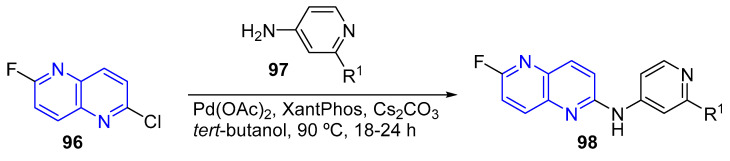
Palladium catalyzed amination of 2-chloro-1,5-naphthyridines.

2-Amino-1,5-naphthyridine derivatives **98** were obtained via Buchwald−Hartwig amination of **96** with the corresponding amines **97** by means of a palladium catalysed incorporation of the amine into the heterocyclic ring of **96** and in the presence of a phosphorated ligand, such as XantPhos (Scheme 34) [38]. Similar Buchwald couplings were used to synthesize other 2,8-disubstituted-1,5-naphthyridine analogues [35], and also compounds featuring a naphthyridine or cyano-naphthyridine segment as the electron acceptor and an acridine unit as the electron donor [34,66].

**Scheme 35:**
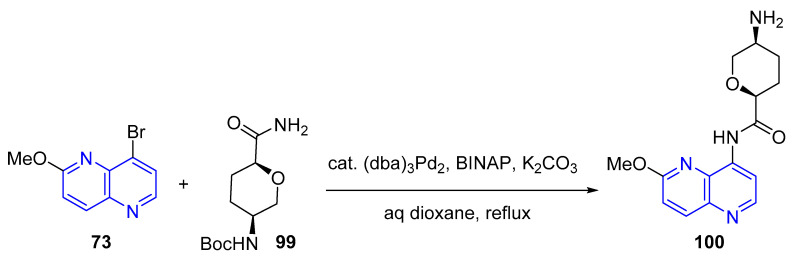
Cross-coupling amination of 4-bromo-1,5-naphthyridine.

Likewise, the preparation of amino-1,5-naphthyridines may involve the coupling of tetrahydropyran-amide **99** with the bromo compound **73** (previously prepared, vide supra, Scheme 28) catalyst by palladium in the presence of (*R*)-(+)-2,2′-*bis*(diphenylphosphino)-1,1´-binaphthalene (BINAP) to form the amide-containing derivative **100** (Scheme 35) [65].

(b) Alcoxylation, Phenoxylation

**Scheme 36:**
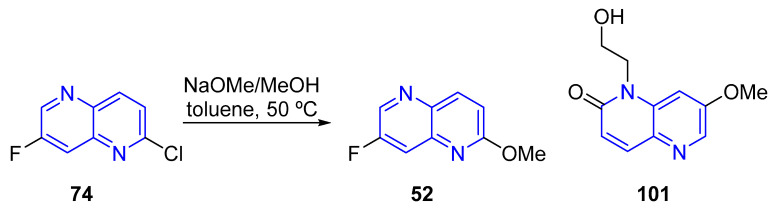
Methoxylation of chloro-1,5-naphthyridines.

In the study to find new methodologies for the preparation of fluorinated intermediates, some methoxylated derivatives/precursors **52** (previously prepared, vide supra, Scheme 15) were synthesized from 1,5-naphthyridine **74** through a S_N_Ar replacing the chloride with sodium methoxide (Scheme 36) [26]. This process has also been reported to obtain the derivative **101** by a nucleophilic displacement of fluorine with sodium methoxide in methanol under heating conditions (Scheme 36) [36].

**Scheme 37:**
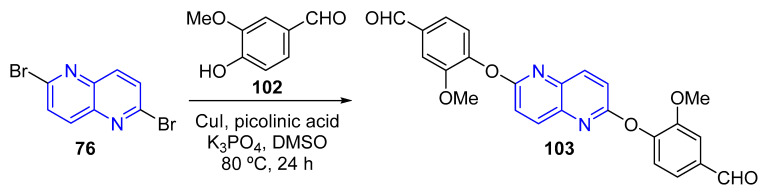
Diphenoxylation of dibromo-1,5-naphthyridines.

The synthesis of dialdehyde **103** (Scheme 37) was realized by the Ullman’s reaction between vanillin **102** and 2,6-dibromo-1,5-naphthyridine **76** (previously prepared, vide supra, Figure 1) in DMSO at 80 °C. In this case, using picolinic acid and copper(I) iodide as catalysts, the expected dialdehyde **103** was obtatined in 86% yield [67,68].

**Scheme 38:**
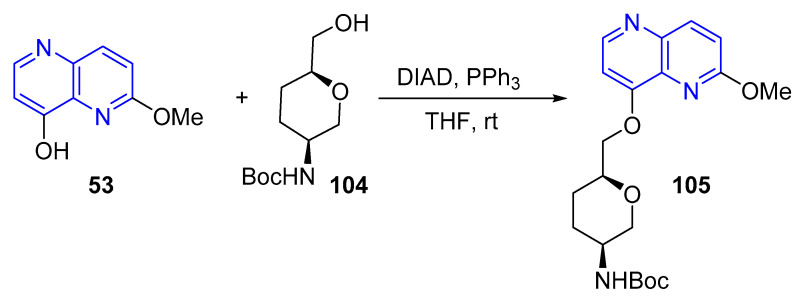
Mitsunobu coupling reaction for the synthesis of alkoxy-1,5-naphthyridine.

On the other hand, starting from a 8-hydroxyl 1,5-naphthyridine **53** (previously prepared, vide supra, Scheme 16) and tetrahydropyran **104**, the derivative **105** was prepared using a Mitsunobu coupling reaction (Scheme 38) [65].

(c) Sulfurization

**Scheme 39:**
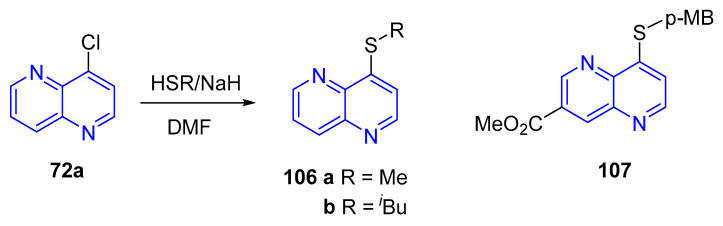
Sulfurization of 4-chloro-1,5-naphthyridines.

The synthesis of 1,5-naphthyridine functionalized indenyl and cyclopentadienyl ligands containing sulfur substituents has been carried out by treatment of chloro derivative **72a** (previously prepared, vide supra, Scheme 27) with sodium methanethiol in the presence of NaH to furnish the methylsulfanyl derivative **106a** (Scheme 39) [62]. Similarly, the nucleophilic substitution of **72a** with led to 2-methylpropane-2-thiol **106b** (Scheme 39) with a moderate yield. The nucleophilic substitution of the chloride precursor with 4-methoxybenzyl-mercaptan (*p*-MBSH) yielded the corresponding sulfurilated intermediate **107** (Scheme 39) [34].

(d) Alkylation

**Scheme 40:**
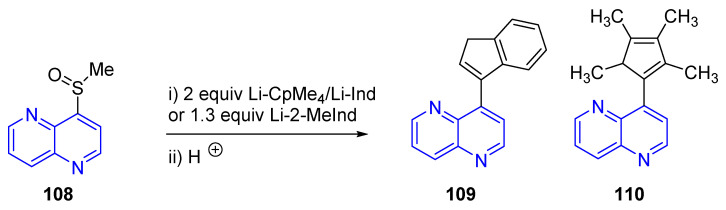
C-C bond formation for the preparation of 4-substituted-1,5-naphthyridines.

The sulfoxide 1,5-naphthyridine derivative **108**, obtained by oxidation of the corresponding sulphide, was reacted with Li-indene (Li-Ind) at low temperature (Scheme 40). The reaction with Li-Ind led to an increased acidity of the indene so that the product was deprotonated in the reaction mixture, and afforded the corresponding indenyl-1,5-naphthyridine derivative **109** with C-C bond formation. Similarly, when the lithium 1,2,3,4-tetramethylcyclopentadienide (Li-CpMe_4_) was used the cyclopentadienyl-1,5-naphthyridine derivative **110** was obtained (Scheme 40) [62].

### 3.3. Oxidations and Reductions

#### 3.3.1. Oxidation

(a) Aromatization of tetrahydro-1,5-naphthyridines

**Scheme 41:**
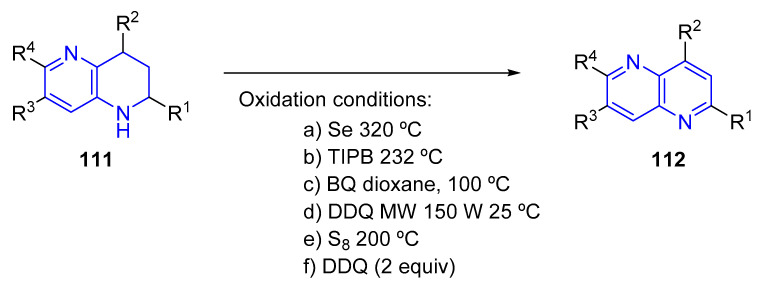
Oxidation of tetrahydro-1,5-naphthyridines to 1,5-naphthyridines.

Tetrahydro- and decahydro-1,5-naphthtyrines can be easily oxidized to the aromatic 1,5-naphthyridines. The oxidation of 1,2,3,4-tetrahydro-1,5-naphthyridines **111** may afford 1,5-naphthyridines **112** at high temperatures (Scheme 41). As examples, selenium at 320 °C or TIPB at 232 °C was heated with the tetrahydroquinoline **111** to prepare 1,5-naphthyridines **112** [69]. High temperatures with benzoquinone (BQ), molecular sulfur or 2,3-dichloro-5,6-dicyano-*p*-benzoquinone (DDQ) were also used to prepare the corresponding 1,5-naphthyridines by the oxidation of the corresponding tetrahydronaphthyridines [45].

**Scheme 42:**
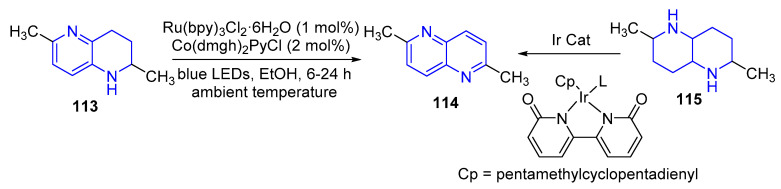
Metal-mediated aromatization of tetra- and octahydro-1,5-naphthyridines.

An acceptorless dehydrogenation of 1,5-naphthyridine **113** by merging visible-light photoredox catalysis and cobalt catalysis at ambient temperature was achieved by using Ru-(bpy)_3_Cl_2_·6H_2_O as a photosensitizer and Co(dmgH)_2_PyCl as a catalyst under optimized reaction conditions (Scheme 42). Aromatic 1,5-naphthyridine **114** was obtained in good yields [70]. A more general methodology is base on homogeneous catalysis using the iridium complexes with a functional bipyridonate ligand for the perdehydrogenation of bicyclic *N*-heterocycles (Scheme 42). Thus, aromatic 2,6-dimethyl-1,5-naphthyridine **114** was efficiently synthesized by this methodology (Scheme 42) [71].

(b) Preparation of 1,5-Naphthyridine *N*-oxides and Their Synthetic Application

**Scheme 43:**
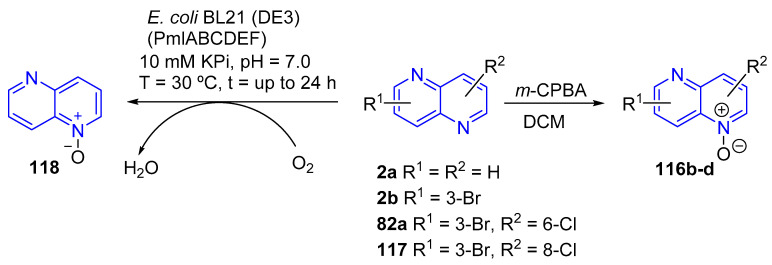
*N*-Oxidation of 1,5-naphthyridines.

*N*-oxides **116** were easily prepared from differently substituted 1,5-naphthyridines **2a**, **2b** (previously prepared, vide supra, Scheme 1), **82a** (previously prepared, vide supra, Scheme 30) and **117** (Scheme 43). In this way, standard reaction conditions (3-chloroperbenzoic acid, m-CPBA) to obtain the corresponding *N*-oxides **106b**–**d** were used to synthesize valuable intermediates for the construction of biologically active molecules [9,10,53]. Alternatively, 1,5-naphthyridines **2a** (previously prepared, vide supra, Scheme 1) were transformed to the corresponding *N*-oxide **118**, at high conversion yields, by a monooxygenase PmlABCDEF recombinantly expressed in *Escherichia coli* as biocatalyst (Scheme 43). The optimized whole cell bioconversions produced a controllable, productive, and green method for the synthesis of 1,5-naphthyridine *N*-oxides. The approach proved to be chemoselective since it was used for the molecules bearing oxidation-sensitive functional groups. Also, this method provides regioselectivity as only specific mono *N*-oxides were formed [72].

Then, after the formation of these *N*-oxides, they would serve for both electrophilic and nucleophilic addition to the 2- and 4-positions. 

**Scheme 44:**
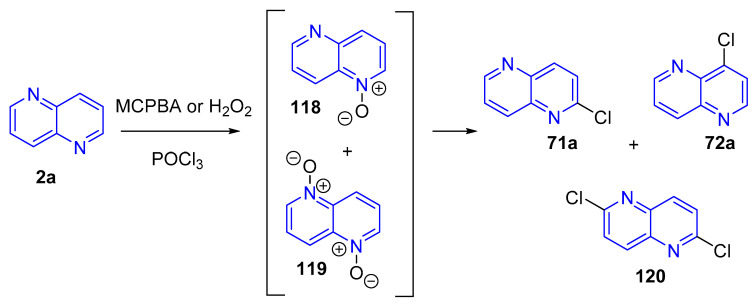
Chlorination of 1,5-naphthyridines by *N*-oxides mediation.

Thus, the reaction of commercial 1,5-naphthyridine **2a** (previously prepared, vide supra, Scheme 1) with POCl_3_, in the presence of a peracid such as *m*-CPBA, was used by Nishimura et al. to obtain a mixture of chlorinated 1,5-naphthyridine **71a** (previously prepared, vide supra, Scheme 26) and **72a** (previously prepared, vide supra, Scheme 27), through the formation of *N*-oxides **118** (Scheme 44) [73]. On the other hand, when starting from the di-*N*-oxide **119** derivative the corresponding dichloro 1,5-naphthyridine **120** was obtained [74].

**Scheme 45:**
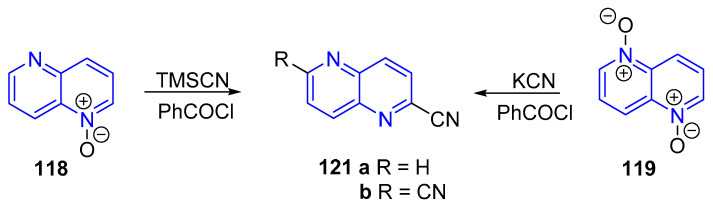
Cyanation of *N*-oxides.

Another application of mono *N*-oxide or di-*N*-oxide derivatives involved the preparation of 2-cyano heterocyclic scaffolds. From *N*-oxides **118** or **119** (previously prepared, vide supra, Scheme 44), after treatment with either tetramethylsilylcyanide (TMSCN) or potassium cyanide, the 2-cyano **121a** or 2,6-dicyano **121b** derivatives were respectively obtained (Scheme 45) [74]. In addition, conventional heating at 130 °C for 1 h of mono *N*-oxide with TMSCN or under microwave conditions (power 50 W) at 130 °C for 5 min led to the compound **121a** in good yield [75].

**Scheme 46:**
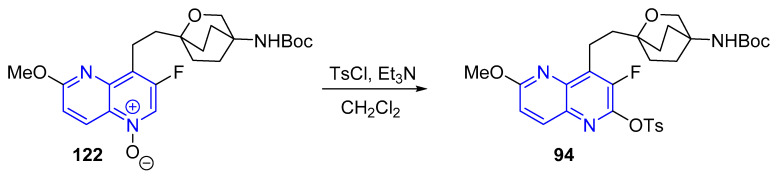
Tosylation of *N*-oxide.

And for the preparation of 2-tosyl substituted naphthyridines, the nucleophilic substitution with TsCl over *N*-oxide **122** allowed the formation of compound **94** (previously prepared, vide supra, Scheme 33) ready for further nucleophilic substitution (Scheme 46) [53]. In some cases, when water and K_2_CO_3_ was used as base, the 1,5-naphthyridin-2(1*H*)-ones were obtained [9].

#### 3.3.2. Reduction

Homogeneous hydrogenation of arenes is a challenging transformation because of the stability derived from the aromaticity. In the particular case of *N*-heteroarenes, this process has to overcome, not only the resonance stability, but also the possible poisoning of the catalyst by either the starting material or the product. Nevertheless, significant developments took place in the past decades and many applications are known for the hydrogenation of model *N*-heterocycles.

**Scheme 47:**
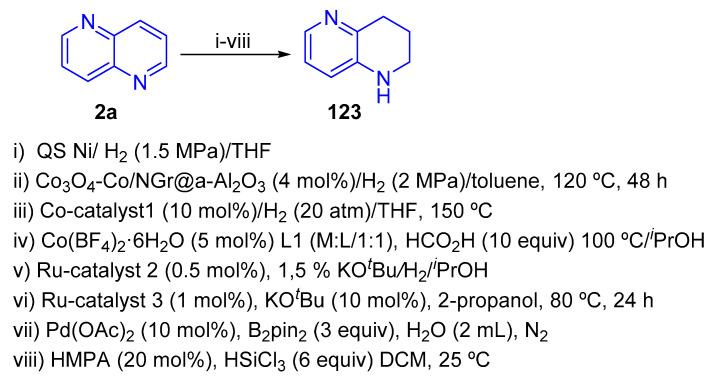
Selective reduction of 1,5-naphthyridine to 1,2,3,4-tetrahydro-1,5-naphthyridine.

**Figure 4:**
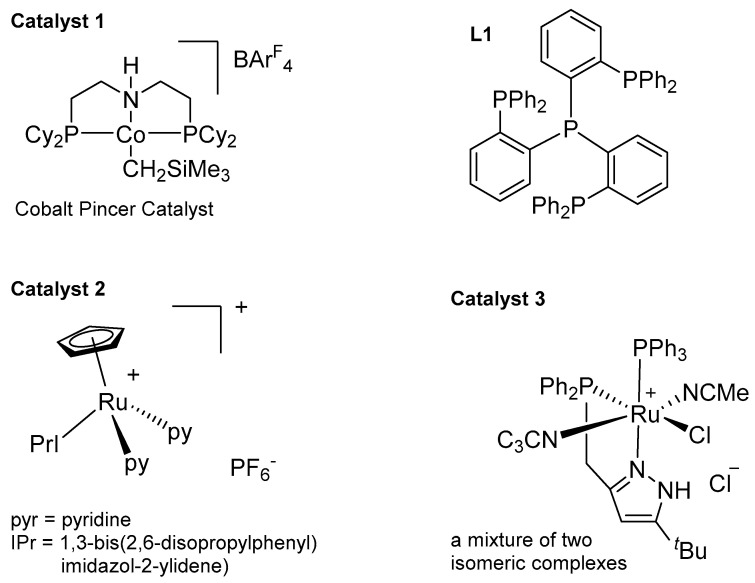
Catalyst and ligand structures.

In this context, quenched skeletal Ni (QS Ni) is an active and selective catalyst for predicable, controllable and selective partial hydrogenation of polycyclic aromatic hydrocarbons (PAHs). When heteroatoms substituted on the skeleton of the PAHs, the hydrogenation reaction would only occur at the substituted ring caused by the strong coordination ability of heteroatoms (i, Scheme 47) [76]. Hydrogenation using hydrogen as reductant and cobalt catalysts such as oxide/cobalt nanoparticles featuring nitrogen-doped graphene layers on alumina (Co_3_O_4_−Co/NGr@α-Al_2_O_3_) [77], or an aminobis(phosphine) [PN(H)P] pincer ligand coordinates to cobalt (**Catalyst 1**, Figure 4) [78], have been performed and 1,2,3,4-tetrahydro-1,5-naphthyridine **123** was obtained (ii-iii, Scheme 47; Figure 4). Transfer hydrogenation of *N*-heteroarenes has been firstly accomplished through homogeneous non-noble metal-catalysis. The combination of Co(BF_4_)_2_·6H_2_O with tris(2-(diphenylphosphino)-phenyl)phosphine **L1** (Figure 4) is able to reduce 1,5-naphthyridines in the presence of other sensitive functional groups, under mild conditions, using formic acid as a hydrogen source (iv, Scheme 47) [79].

In the presence of KO*^t^*Bu, *N*-heterocyclic carbene-supported half-sandwich complex [Cp(IPr)Ru(pyr)2]-[PF6] (**Catalyst 2**, Figure 4) catalyzes transfer hydrogenation of *N*-heterocycles in isopropanol at the catalyst loading of 0.5 mol%. Under these conditions, 1,5-naphthyridine **2a** (previously prepared, vide supra, Scheme 1) could be hydrogenated in good yields (v, Scheme 47; Figure 4) [80]. In another approach, the transfer hydrogenation of 1,5-naphthyridines could be accomplished in good yields, using ruthenium(II) complexes (**Catalyst 3**) supported by a pyrazole-phosphine ligand (vi, Scheme 47, Figure 4) [81].

A Pd-catalyzed transfer hydrogenation of various *N*-heteroaromatic compounds with *bis*(pinacolato)diboron (B2pin2), as a mediator in environmentally benign water as both solvent and hydrogen donor has been disclosed. This reaction proceeded under ambient temperature and 1,5-naphthyridine **2a** (previously prepared, vide supra, Scheme 1) underwent selective hydrogenation in one of the pyridine rings (vii, Scheme 47) [82].

A method for the hexamethylphosphoric triamide (HMPA)-catalyzed metal-free transfer hydrogenation of pyridines by using trichlorosilane hydride (HSiCl_3_) has been developed. The suitability of this method for the reduction of other *N*-heteroarenes has also been demonstrated. Thirty-three different substrates have been reduced to designed products in 45–96% yields. 1,5-Naphthyridine **2a** (previously prepared, vide supra*,* Scheme 1) could only be partially reduced to the corresponding tetrahydro derivative **123** (viii, Scheme 47) [83].

**Scheme 48:**
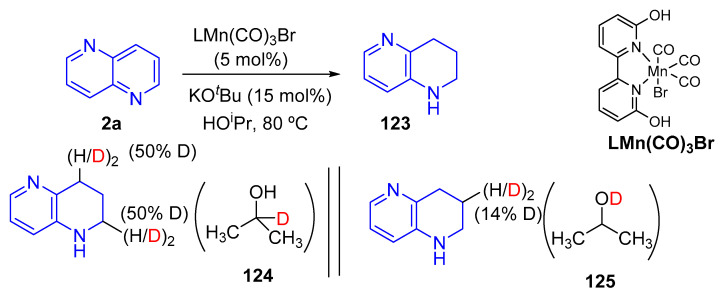
Hydrogen/deuterium exchange in selective reduction processes.

For the first time, Mn catalyzed transfer hydrogenation of aromatic *N*-heterocycles, which normally involve precious metal catalysts such as Ru, Rh and Ir complexes, has been reported for 1,5-naphthyridine to give the tetrahydro derivative **123** (previously prepared, vide supra, Scheme 47) in the presence of KO*^t^*Bu*/^i^*PrOH (Scheme 48) in excellent yield. Furthermore, deuterium labeling experiments using 1,5-naphthyridine has been performed. Interestingly, when 2-propanol-2-*d_1_* **124** was used as the solvent and hydrogen source, selective incorporation of deuterium was observed at the 2- and 4-positions, corresponding to incorporation of one D-atom to each of the CH_2_ groups (i.e., 50% deuteration at each of the methylene positions), while the 3-position did not contain D atom at detectable levels (Scheme 48). Contrarily, when 2-propanol-OD **125** was used, no deuterium was detected in 2- and 4-positions, and some deuterium was detected only in the 3-position (*ca.* 14% D incorporation in the CH_2_ group, Scheme 48) [84].

**Scheme 49:**
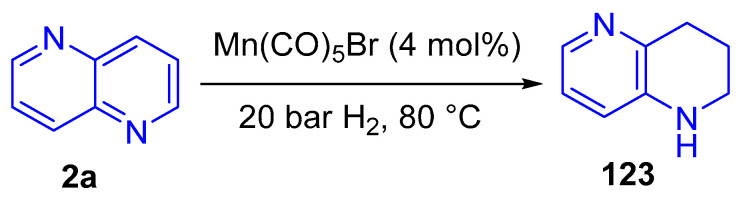
Manganese catalyzed reduction of 1,5-naphthyridine.

Recently, a chemoselective reduction of 1,5-naphthyridine **2a** (previously prepared, vide supra, Scheme 1) by molecular hydrogen has been described under very mild reaction conditions using a simple Mn(II) complex, manganese pentacarbonyl bromide (Scheme 49) [85].

**Scheme 50:**
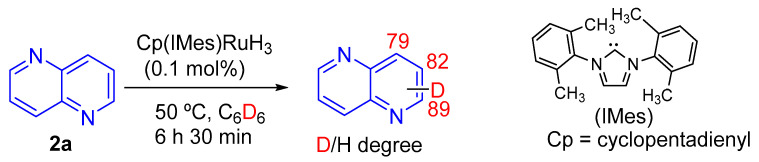
Hydrogen/deuterium exchange processes by using ruthenium catalyst.

In contrast, H/D exchange reactions in *N*-heterocyclic substrates have received much less attention. It was found that modification of the original catalyst by using the isolobal relationship between *N*-heterocyclic carbenes (NHCs) and phosphines affords a new catalytic system capable of H/D exchange in *N*-heterocyclic substrates under very mild conditions and at low catalyst loading. NHC-supported hydride complexes of ruthenium (Cp(IMes)RuH_3_) showed excellent catalytic activity in the H/D exchange of *N*-heterocyclic compounds under mild conditions and low catalyst loading (Scheme 50) [86].

**Scheme 51:**
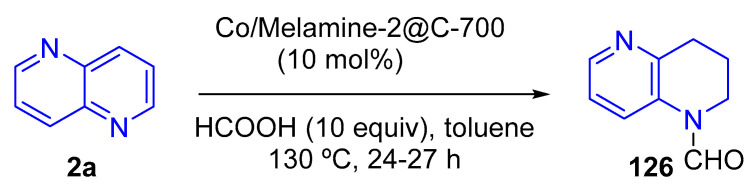
Cobalt catalyzed reduction and *N*-formylation sequence of 1,5-naphthyridine.

Nitrogen modified heterogeneous cobalt catalysts supported on carbon were prepared in water using inexpensive melamine or melamine resins as nitrogen source. The optimal catalyst Co/Melamine-2@C-700 allows the selective transfer hydrogenation of diverse heteroarenes using formic acid as reductant (Scheme 51). Compared to most known transfer hydrogenations, the addition of base is not necessary to obtain sufficient catalyst activity. In speed of this, the reduction of 1,5-naphthyridines required a 2.5 equiv. of Et_3_N and the corresponding formylated product **126** was obtained in a moderate yield (38%) [87].

**Scheme 52:**
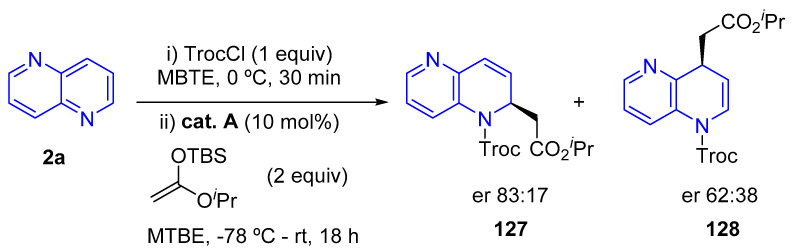
Enantioselective preparation of dihydro-1,5-naphthyridines.

**Figure 5:**
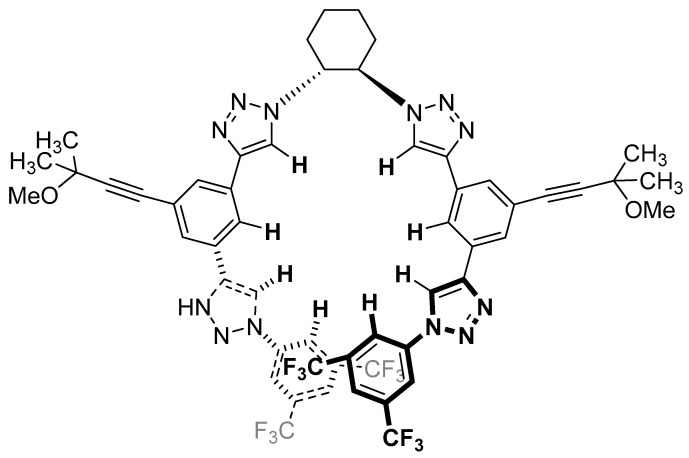
Catalyst A structure.

The first enantioselective nucleophilic dearomatization of diazarenes using an anion-binding organocatalysis approach has been developed. Tetrakistriazole-based H-bond donor catalysts type **A** (Figure 5) were the best catalysts. In the case of 1,5-naphthyridine **2a** (previously prepared, vide supra, Scheme 1), which has one nitrogen atom in each ring, the challenge was the control of the regioselectivity, since both the C-4 and C-2 positions of each heteroaromatic ring are prone to nucleophilic addition. A good regioselectivity of 95:5 was obtained in favor of the C-2-addition product **127** when 2,2,2-trichloroethyl chloroformate (trocCl) was used in the presence of methyl *tert*-butyl ether (MBTE) (Scheme 52). After the separation from the minor 4-addition product **128**, the 2-addition compound **127** was obtained in 86% yield and a good 83:17 enantiomeric ratio [88].

**Scheme 53:**
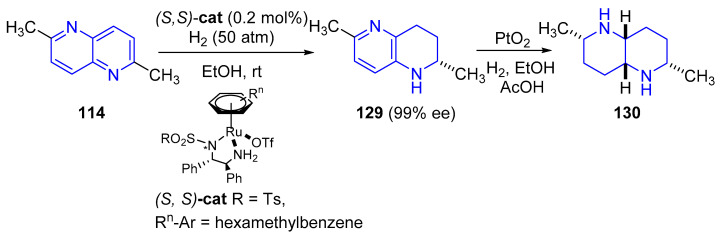
Enatioselective reductions of 1,5-naphthyridines.

Selective reduction of one of the pyridine or both pyridine rings moieties of 1,5-naphthyridines can be achieved. Thus, 2,6-disubstituted 1,5-naphthyridines **114** (previously prepared, vide supra, Scheme 42) were asymmetrically hydrogenated catalyzed by chiral cationic ruthenium diamine complexes, with good to excellent enantioselectivities. A wide range of 1,5-naphthyridine derivatives were efficiently hydrogenated to give 1,2,3,4-tetrahydro-1,5-naphthyridines **129** with up to 99% ee and full conversions (Scheme 53). Subsequently, the optically pure (*S*)-**129** was smoothly reduced using PtO_2_ as a catalyst to provide (2*S*,6*S*,9*R*,10*R*)-2,6-dimethyl-1,5-diaza-*cis*-decalin **130** in 87% yield as the sole product without other diastereomers (Scheme 53). In view of the unfavorable steric hindrance between the surface of the PtO_2_ catalyst and the methyl group in the 2-position, the opposite face of molecule is more inclined to approach the heterogeneous catalyst surface [89].

### 3.4. C-C Bond Constructions, Cross-Couplings

The generation of metalated aromatics has become extremely important for the introduction of substituents, especially carbon substituents, by subsequent reaction with an electrophile.

**Scheme 54:**
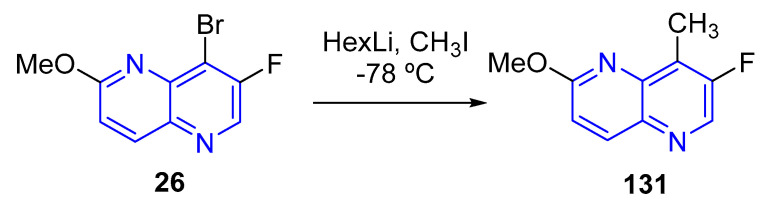
Methylation of bromo-1,5-naphthyridine with organolithium reagent.

In a study to find new methodologies for the late-stage introduction of fluorine into advanced intermediates, metal-halogen exchange was used to introduce a methyl group of the 1,5-naphthyrine **26** (previously prepared, vide supra, Scheme 8) leading to the methylated-1,5-naphthyridine **131** with good yield (Scheme 54) [26].

**Scheme 55:**
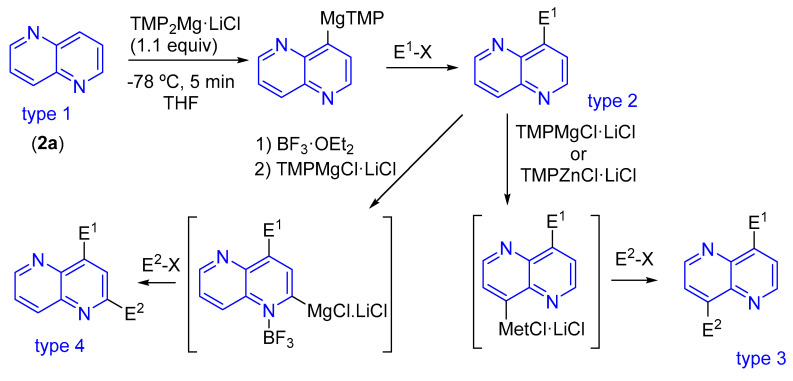
Knochel difunctionalization of 1,5-naphthyridines mediated by organometallic reagents.

Knochel et al. reported an excellent and efficient set of regioselective metalations and functionalizations of the 1,5-naphthyridine scaffold using Zn-, Mg-, and tetramethylpiperidyl (Li-TMP) bases. It was possible to metalate the 1,5-naphthyridine scaffold regioselectively providing mono-, di-, tri-, or tetrasubstituted naphthyridines [90]. Thus, the precomplexation of type 1 naphthyridine with the magnesium amide TMP_2_Mg·2LiCl was found to induced a magnesiation at the C-4-position, which after quenching with several electrophiles (E^1^-X), led to 4-substituted 1,5-naphthyridines of type 2 (Scheme 55). The presence of a substituent E1 on C-4 of the naphthyridine type 2 prevented further complexation of a metallic amide at N5 and favored the precomplexation of TMPMgCl·LiCl or TMPZnCl·LiCl at N1. This favored a directed magnesiation (or zincation) at C-8 providing, after quenching with several electrophiles (E^2^-X), the regioselectively defined 4,8-substituted 1,5-naphthyridines of type 3. Alternatively, the naphthyridines type 2 were treated with BF_3_·Et_2_O, which resulted in a regioselective magnesiation at C-2 using TMPMgCl·LiCl. After quenching with an electrophile (E^2^-X), 2,4-disubstituted 1,5-naphthyridines of type 4 were obtained (Scheme 55).

**Scheme 56:**
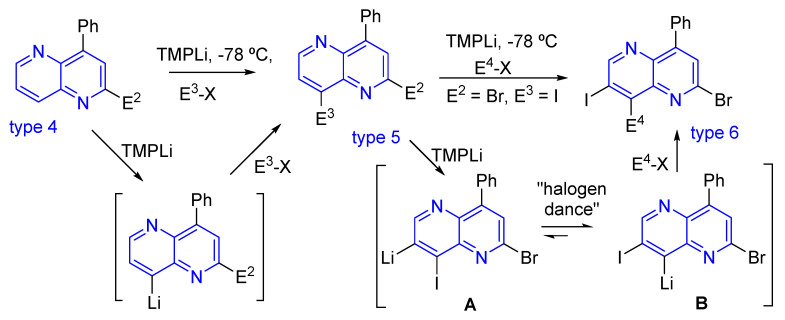
Knochel functionalization of disubstituted 1,5-naphthyridines mediated by organometallic reagents.

A third functionalization of 2,4-disubstituted 1,5-naphthyridines of type 4 (E^1^ = Ph) with stronger lithium amide TMPLi readily coordinated to N1 and lithiated the closest C-H bond at C-8. The 8-lithionaphthyridine was trapped by several electrophiles to give 1,5-naphthyridine type 5 (Scheme 56). A fourth functionalization was achieved using a lithium mediated “halogen dance” rearrangement. Thus, the reaction of the trifunctionalized naphthyridine type 5 with TMPLi followed by quenching with water gave the corresponding regioisomer type 6. The formation was explained by a lithiation at position 7, leading to the lithium intermediate **A**. The following very fast “halogen dance” provided the more stable C-8-lithiated naphthyridine **B**. Reaction of **A** with various electrophiles (E^4^-X) gave the 2,4,7,8-tetrasubstituted 1,5-naphthyridines type 6 in good yields (Scheme 56).

**Scheme 57:**
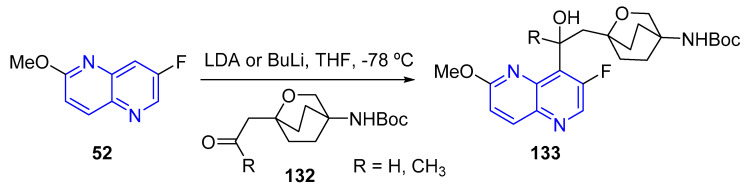
Synthesis of alcohol derivatives from carbonyl compounds and metallated 1,5-naphthyridine.

Other metalation examples have been applied in the structure-activity relantionship (SAR) study of different linkers for the preparation of some active compounds. Nucleophilic addition of deprotonated 1,5-naphthyridine **52** (previously prepared, vide supra, Scheme 15) to aldehyde or ketone **132** after treatment with *n*-BuLi or lithium diisopropylamide (LDA) produced the corresponding alcohol **133** (Scheme 57) [91].

**Scheme 58:**
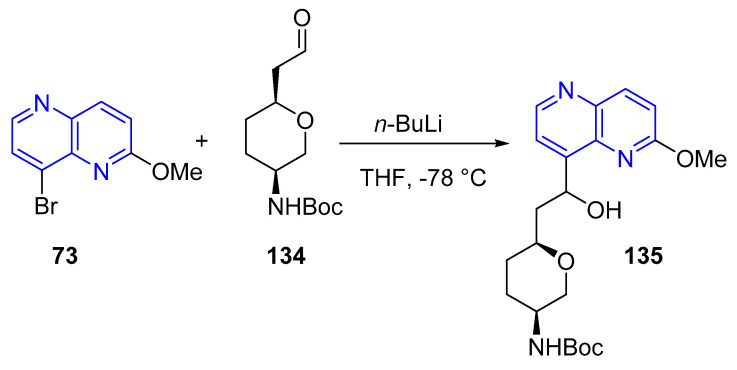
Synthesis of secondary alcohol derivative from aldehyde and metallated 1,5-naphthyridine.

Similarly, a general synthetic access to some 1,5-naphthyridines involved the reaction of bromo derivatives **73** (previously prepared, vide supra, Scheme 28) with *n*-butyl lithium at −78 °C generating the lithio anion that reacted with aldehyde **134** to yield benzylic alcohol **135** (Scheme 58), ready for further derivatization [65].

The 1,5-naphthyridine ring has been subjected to a wide variety of C-C bond forming cross-coupling reactions, such as Heck, Suzuki, Negishi, among others.

**Scheme 59:**
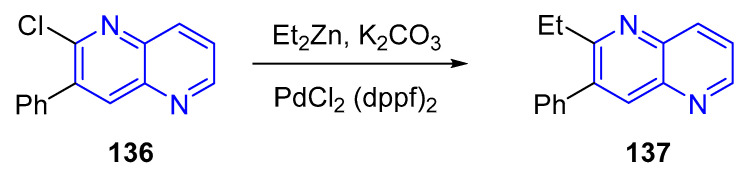
Cross-coupling ethylation of 1,5-naphthyridine.

An expert opinion of a patent was given by Norman where 1,5-naphthyridines were prepared from commercially available 1,5-naphthyridine derivatives with Et_2_Zn in the presence of a palladium catalyst (Scheme 59). A novel C-C bond construction occurred in position 6 of the 1,5-naphthyridine ring. With this method 10 derivatives were synthesized [92].

**Scheme 60:**
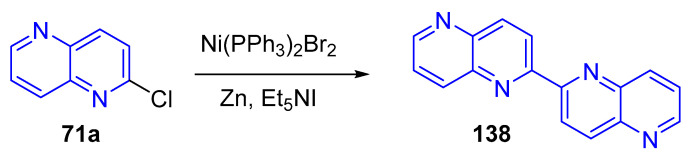
Metal catalyzed dimerization of 1,5-naphthyridine.

In order to prepare the 2,2´-bi-1,5-naphthyridine dimer **138**, a new ambivergent ligand in metal complexes, a previously obtained 2-chloro-1,5-naphthyridine **71a** (previously prepared, vide supra, Scheme 26) was dimerized by a new synthetic route which, included a Ni(0)-catalysed coupling process (Scheme 60) [63].

**Scheme 61:**
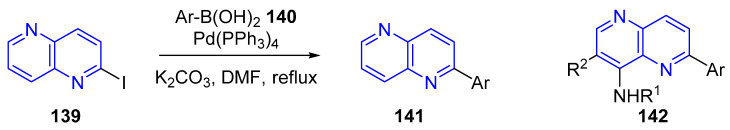
Suzuki cross-coupling reaction to obtain 2-aryl-1,5-naphthyridines.

Boronic acids have been widely used in Suzuki cross coupling reaction. This strategy favors the incorporation of alkyl and/or aryl groups in different positions of the 1,5-naphthyridine. Synthesis of 2-aryl-1,5-naphthyridine derivatives **141**, based on the cross-coupling reaction of commercially available 2-iodo-1,5-naphthyridine **139** with aromatic and heteroaromatic boronic acids **140** in the presence of Pd(PPh_3_)_4_, was reported (Scheme 61). Easily available starting materials, moderate reaction conditions, short reaction time, and higher yields of the products were the major advantages of this method [93]. A Suzuki coupling by using CsCO_3_ as base and Pd(dppf)Cl_2_ as catalyst was also completed for the preparation of 2-substituted 1,5-naphthyridines **142** (Scheme 61) [21].

**Scheme 62:**
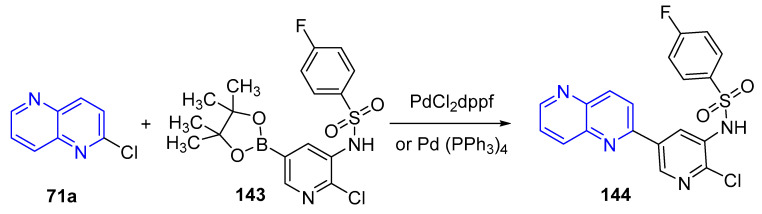
Suzuki cross-coupling reaction to obtain substituted 2-pyrido-1,5-naphthyridine.

Nishimura et al. prepared 2-aryl-1,5-naphthyridine derivative **144** based on the coupling reaction between boronic acid pinacol ester of functionalized 4-sulphonylamino pyridine **143**, and chloro 1,5-naphthyridine **71a** (previously prepared, vide supra, Scheme 26) in the presence of Pd complexes (Scheme 62) [73].

**Scheme 63:**
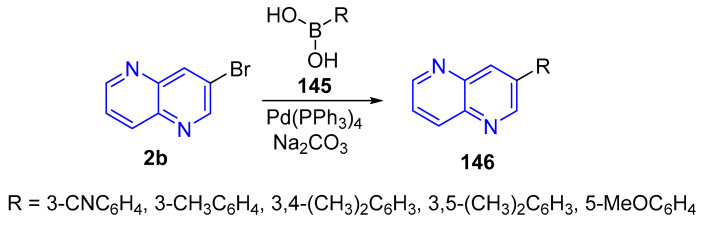
Cross-coupling reaction to obtain 3-aryl-1,5-naphthyridines.

A novel regioisomeric series of 3-aryl-1,5-naphthyridine derivatives **146** have been constructed by Galatsis et al. using a Suzuki cross coupling reaction (Scheme 63). The method involved 3-bromo-1,5-naphthyridine **2b** (previously prepared, vide supra*,* Scheme 1) and boronic acid **145** with palladium as catalyst to give derivatives **146** [94].

**Scheme 64:**
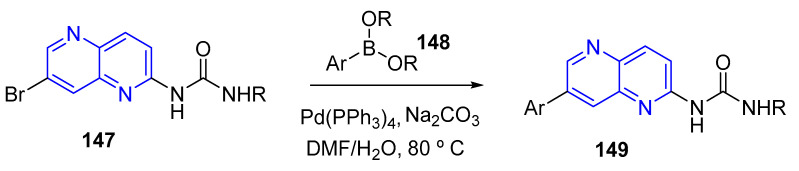
Cross-coupling reaction to obtain 7-aryl-1,5-naphthyridines.

For example, during the synthesis of (7-aryl-1,5-naphthyridin-2-yl)ureas **149** aromatic moieties were introduced at position 7 of compound **147** under a palladium-catalyzed Suzuki–Miyaura type reaction, using boronic acids or esters **148**. The target compounds **149** were obtained in low to good yields (Scheme 64) [10].

**Scheme 65:**
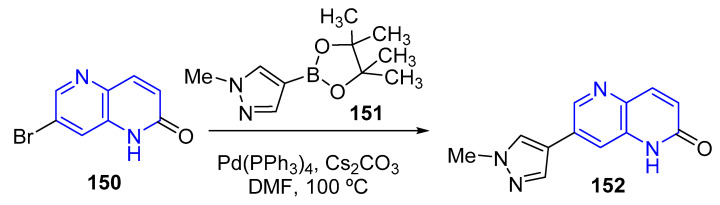
Suzuki reaction to obtain 7-pyrazolyl-1,5-naphthyridinone.

Another example can be found for the synthesis of some novel 1,5-naphthyridine derivatives **152**, which were prepared in 83% yield by Suzuki reaction under the catalysis of Pd(PPh_3_)_4_ with Cs_2_CO_3_ as the alkali (Scheme 65) [9].

**Scheme 66:**
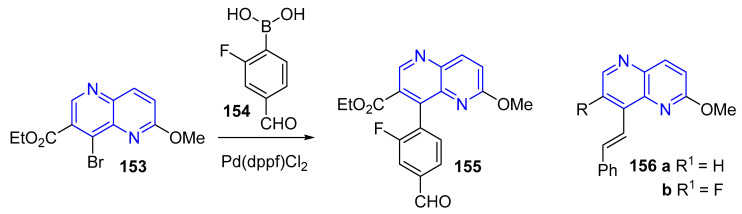
Cross-coupling reaction for the preparation of 8-substituted-1,5-naphthyridines.

A Suzuki-Miyaura protocol was used to combine 4-formylarylboronic acid **154**, Pd(dppf)Cl_2_ as catalyst and K_2_CO_3_ as a base with a previously prepared 8-bromo-1,5-naphthyridine **153** in order to prepare the 8-(4-formylaryl)-2-methoxynaphthyridine **155** in high yield (Scheme 66) [95]. The Suzuki coupling of 8-substituted-1,5-naphthyridine alkenes **156** started from bromides with *trans*-phenylvinyl boronic acid under the catalysis of Pd(PPh_3_)_4_ giving rise to the respective phenyl alkenes (Scheme 66) [65].

**Scheme 67:**
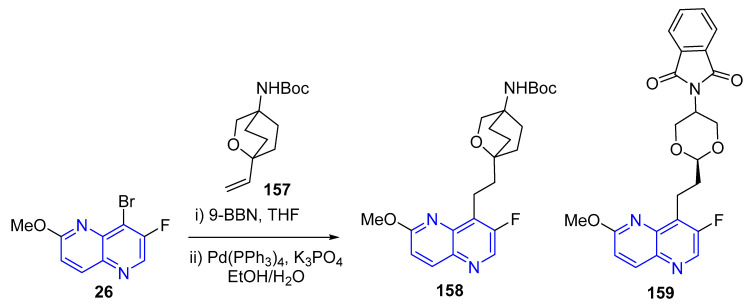
Suzuki reaction for the synthesis of functionalized 1,5-naphthyridines.

The hydroboration of vinyl oxabicyclooctane **157** followed by Suzuki coupling with 8-bromo-1,5-naphthyridine **26** (previously prepared, vide supra, Scheme 8), mediated by Pd(PPh_3_)_4_, and K_3_PO_4_ allowed the formation of the functionalized heterocycle **158** (Scheme 67) [25,53]. Similarly, hydroboration of the corresponding alkene with 9-borabicyclo(3.3.1)nonane (9-BBN) followed by Suzuki coupling in the presence of Pd(dppf)Cl_2_ as catalyst and Cs_2_CO_3_ as base afforded the corresponding dioxane-linked derivatives **159**, ready for further transformation (Scheme 67) [96].

**Scheme 68:**
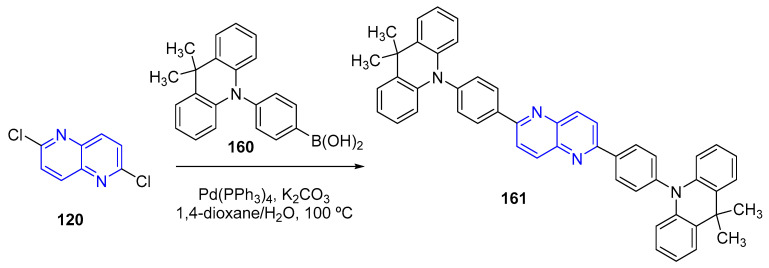
Difunctionalization of 1,5-naphthyridine by Suzuki reactions.

This strategy was also used for the double incorporation of alkyl or aryl groups into the skeleton of 1,5-naphthyridines. The last step to obtain the 2,6-*bis*(4-(9,9-dimethylacridin-yl)phenyl)-1,5-naphthyridine **161** was a Suzuki-Miyaura coupling between the chlorinated 2,6-naphthyridine **120** (previously prepared, vide supra, Scheme 44) and (4-(9,9-dimethylacridin-10(9*H*)-yl)phenyl) boronic acid **160** (Scheme 68) [40].

**Scheme 69:**
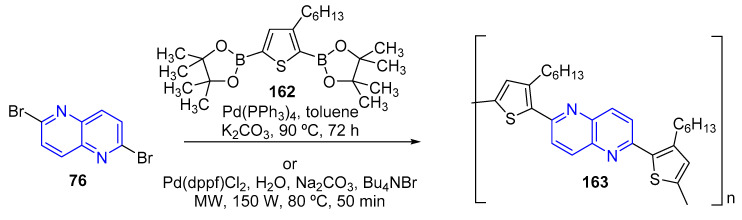
Cross-coupling polymerization of dibromo-1,5-naphthyridine.

In a similar way, the synthesis of poly [1,5-naphthyridine-(3-hexylthiophene)] semi-conducting polymer **163** has been accomplished by adopting both conventional and microwave-assisted Suzuki-Miyaura cross-coupling reaction between 3-hexylthiophene-2,5-diboronic ester **162** and 2,6-dibromo-1,5-naphthyridine **76** (previously prepared, vide supra, Figure 1) (Scheme 69) [40,97].

**Scheme 70:**
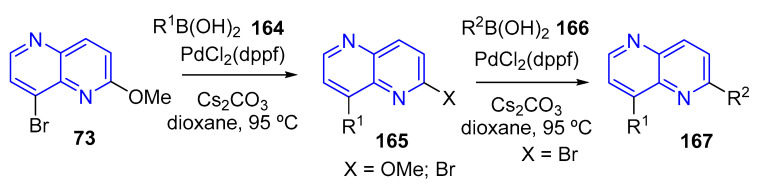
Sequential Suzuki reaction.

A series of 2,8-disubstituted-1,5-naphthyridine analogues were synthesized. The brominated key intermediate **73** (previously prepared, vide supra, Scheme 28) was subjected to a Suzuki cross-coupling reaction using commercially available boronates **164** (Scheme 70). A second Suzuki reaction using boronates **166** furnished the target compounds **167** [35].

**Scheme 71:**
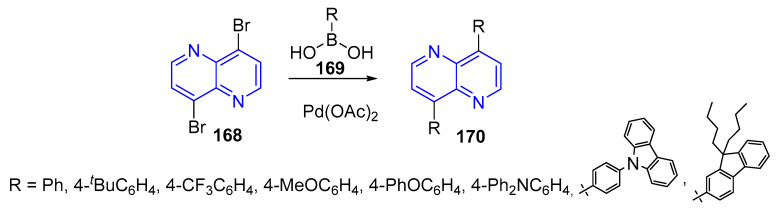
Double arylation of 1,5-naphthyridines by cross-coupling reactions.

Likewise, 4,8-substituted 1,5-naphthyridines **170** have been synthesized in yields of 41.4–75.8% from 4,8-dibromo-1,5-naphthyridine **168** and a wide range of boronic acids **169** in the presence of palladium acetate catalyst (Scheme 71) [20].

**Scheme 72:**
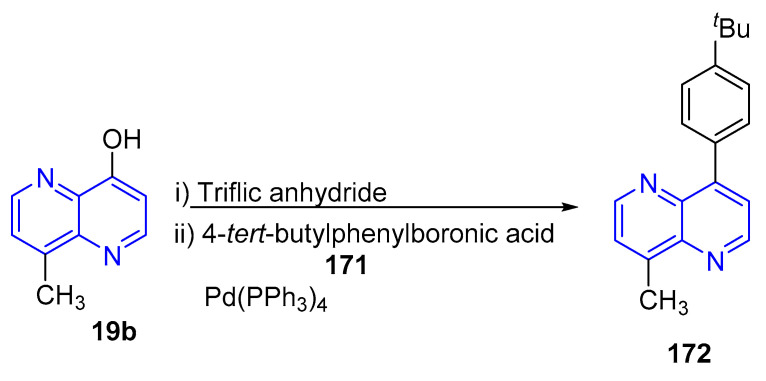
Suzuki coupling of hydroxy-1,5-naphthyridine mediated by triflic anhydride.

4-(4-*tert*-Butylphenyl)-8-methyl-1,5-naphthyridine **172** was prepared by the conversion of 4-hydroxy-8-methyl-1,5-naphthyridine **19b** (previously prepared, vide supra, Scheme 6) to its triflate, followed by Suzuki coupling with 4-*t*-butylphenylboronic acid **171** (Scheme 72) [98].

**Scheme 73:**
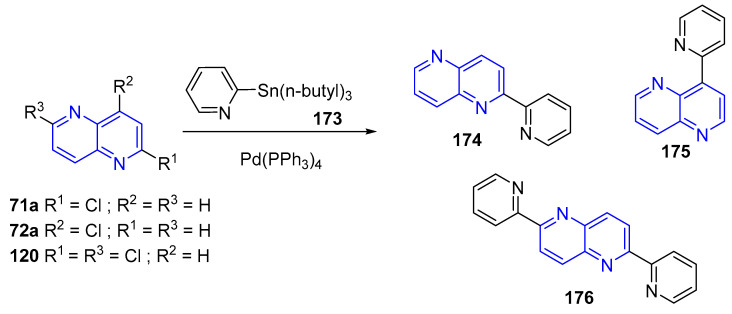
Stille reaction to obtain pyridine-1,5-naphthyridines.

Other cross-coupling reactions have also been used for 1,5-naphthyridines. The Stille reaction was applied for the coupling between 1,5-naphthyridines and other heterocycles. Thus, 1,5-naphthyridine derivatives have been synthesized as new linkers for the construction of bridging ligands. These derivatives were prepared from the corresponding chloro-1,5-naphthyridine **71a** (previously prepared, vide supra, Scheme 26), **72a** (previously prepared, vide supra, Scheme 27) and **120** (previously prepared, vide supra, Scheme 44), which underwent Stille type reaction with 2-(tributylstannyl)pyridine **173** to afford the corresponding 1,5-naphthyridine derivatives **174**–**176** (Scheme 73) [74]. 

**Scheme 74:**
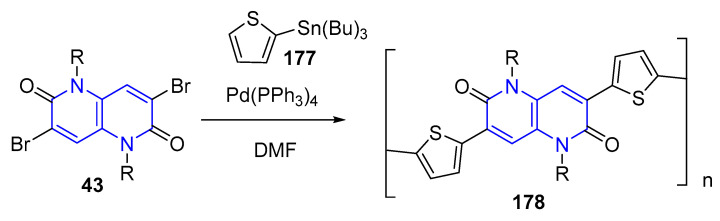
Polymerization Stille coupling reaction of dibromo-1,5-naphthyridinones.

This process has also been used for the synthesis of polymers **178**. Both monomers and polymers were synthesized using typical Stille coupling reaction conditions, between 1,5-naphthyridine **43** (previously prepared, vide supra, Scheme 13) and 2-(tributylstannyl)thiophene **177** (Scheme 74) [37,49].

**Scheme 75:**
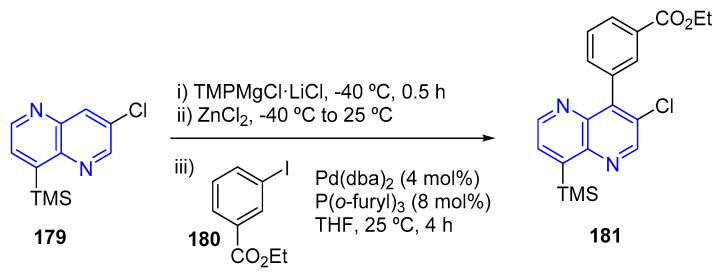
Negishi cross-coupling of 1,5-naphthyridine.

In a synthetic strategy developed by Knochel et al. involving silylated acetal-containing organometallic reagents for the construction of various polyfunctionalized 5-, 6-, and 7-membered heterocycles, Negishi coupling was also applied. The corresponding naphthyridine **179**, which was readily treated with TMPMgCl·LiCl and after Negishi cross-coupling with ethyl 3-iodo-benzoate **180**, provided the corresponding 4-substituted 1,5-naphthyridine **181** in 72% yield (Scheme 75) [39].

**Scheme 76:**
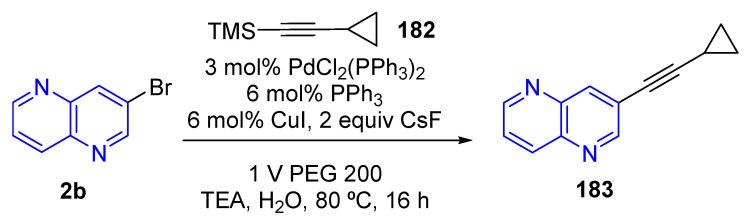
Sonogashira coupling reaction of 3-bromo-1,5-naphthyridine.

Recently, practical and mild conditions have been developed for the Sonogashira synthesis of a variety of alkynyl benzenes and heteroarenes **183** in good to excellent yields, using CsF-mediated in situ TMSalkyne desilylation (Scheme 76). This method is accompained by excellent functional group tolerance and simple purification, which allows large-scale industrial applications. This one-pot protocol enables a high-yielding Sonogashira coupling with volatile alkynes by avoiding challenging isolation of free alkynes [99].

**Scheme 77:**
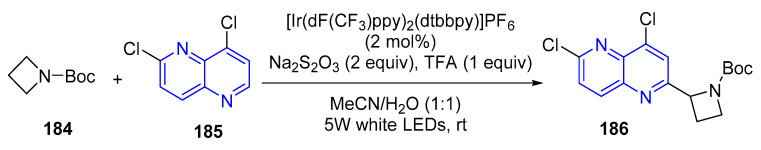
Cross-coupling reaction under Minisci photoredox conditions.

In another study, 1,5-naphthyridines **185** have been directly C-H functionalized in a single step with a variety of *tert*-butoxycarbonyl (Boc)-protected aliphatic and cyclic amines **184** under Minisci photoredox conditions from unactivated coupling partners (Scheme 77). The high functional group tolerance and versatility of these transformations make them directly applicable to elaborate compounds **186** at the very last stage of synthesis [100].

### 3.5. Modification of the Side Chain

**Scheme 78:**
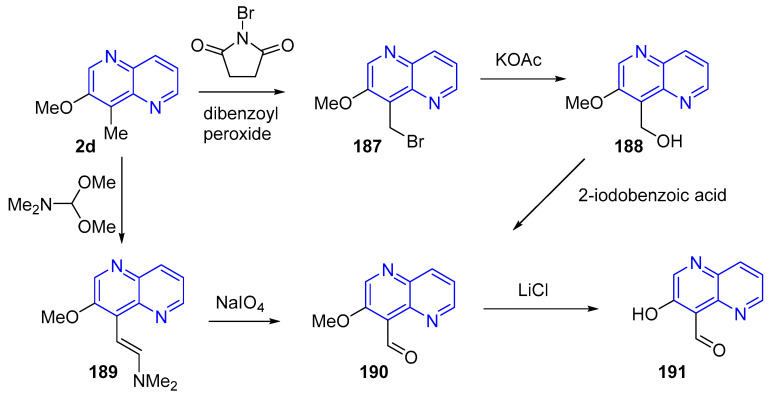
Preparation of 1,5-naphthyridine-4-carbaldehyde from 1,5-naphthyridine **2d**.

The substituents on the 1-5-naphthyridine ring have been subjected to a wide range of chemical transformations. Researchers at Pfizer modified the 2-methyl-3-methoxy-1,5-naphthyridine **2d** (previously prepared, vide supra, Scheme 1) to give, after three steps, the 3-hydroxy-1,5-naphthyridine-4-carbaldehyde **191** necessary for the preparation of a novel azetidinyl tethered ketolide series (Scheme 78) [13,14]. Before, the same group prepared the same carbaldehyde derivative **191** by synthesizing the hydroxyl derivative **188** followed by the proteccion of naphthyridine **190** [12].

**Scheme 79:**
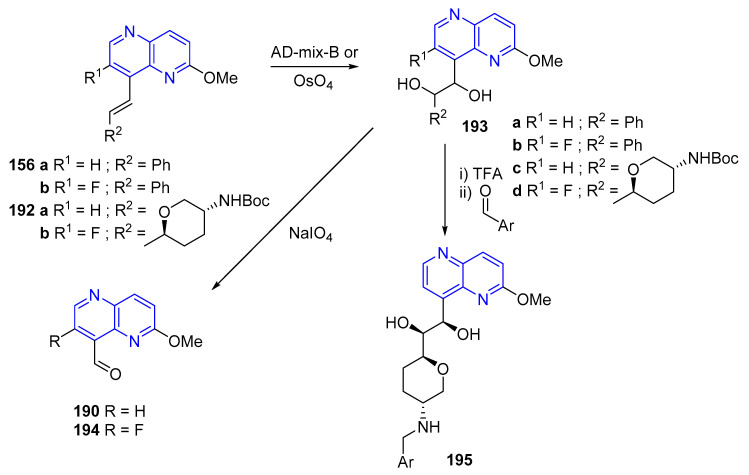
Preparation of 1,5-naphthyridine derivatives **190**, **194** and **195**.

For the synthesis of antibacterial drugs, after the preparation of (*E*)-alkenes **156a**,**b** (previously prepared, vide supra, Scheme 66) and **192a**,**b** with *E*/*Z* selectivities bigger than 6/1, the (1R,2S)-*syn*-diols **193a**–**d** were obtained by using AD-mix-β or OsO_4_ as the dihydroxylating reagent. Reaction with NaIO_4_ yielded the corresponding aldehydes **190** (previously prepared, vide supra, Scheme 78) and **194**. From compounds **193d**, after removal of the *tert*-butoxycarbonyl (Boc) group with TFA, the resulting amine was transformed to **195** using different aromatic aldehydes via reductive amination reaction (Scheme 79) [65].

**Scheme 80:**
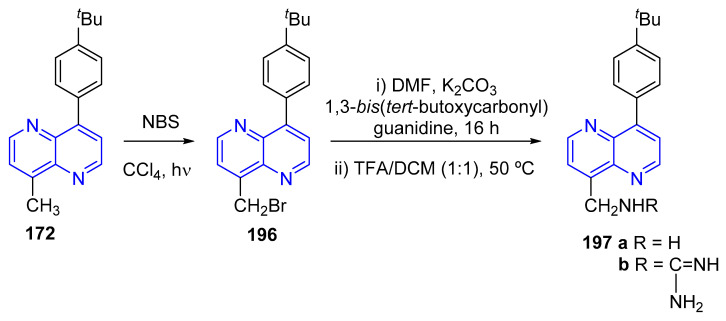
Preparation of compounds **197**.

To achieve the synthesis of aminomethyl substituted naphthyridines, methyl 1,5-naphthyridine **172** (previously prepared, vide supra, Scheme 72) was reacted with *N*-bromosuccinimide to afford the bromo methyl derivative **196** (Scheme 80). The treatment of this compound with DMF and 1,3-*bis*(*tert*-butoxycarbonyl)guanidine yielded the derivatives **197a**,**b** [98].

**Scheme 81:**
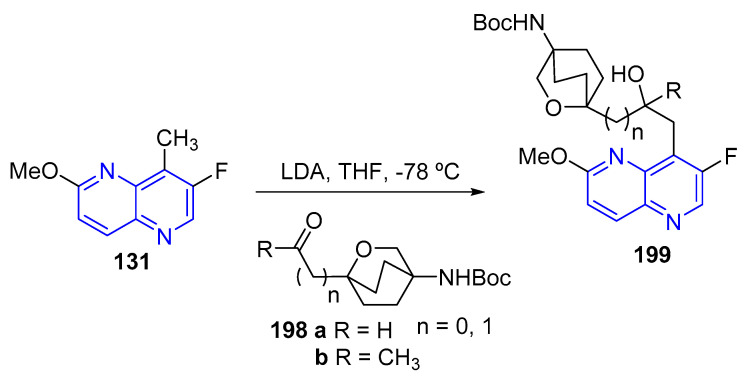
Synthesis of alkohol derivatives **199**.

On the other hand, Fukuda et al. have been involved in the study of a series of oxabicyclooctane-linked as novel bacterial topoisomerase inhibitors (NBTIs). A hydroxyl group in the linker was optimal for activity. The preparation of these alcohols **199** took place through addition of the deprotonated 8-methyl-1,5-naphthyridine **131** (previously prepared, vide supra, Scheme 54) to the corresponding aldehyde **198a** or ketone **198b** (Scheme 81) [25,91].

**Scheme 82:**
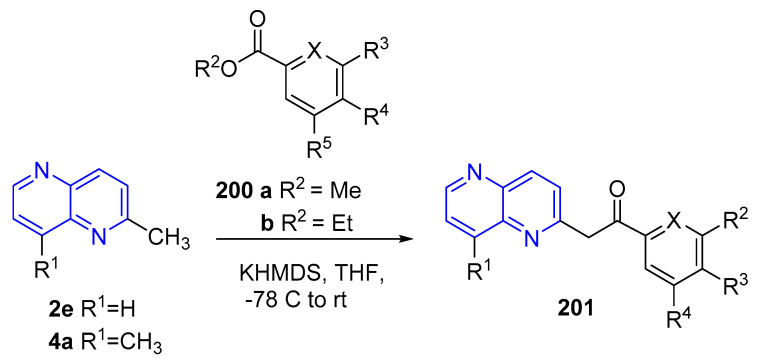
Synthesis of carbonylic compounds **201**.

The precursors of biologically interesting naphthyridine aminothiazoles are the carbonyl derivatives **201** (Scheme 82). They were readily prepared by metalation of 2-methyl-1,5-naphthyridines **2e** (previously prepared, vide supra, Scheme 1) or **4a** (previously prepared, vide supra, Scheme 2), in the presence of potassium *bis*(trimethylsilyl)amide at low temperature, and treatment with the corresponding aryl or pyridyl (X = N) esters **200**. The resulting ketones **201** were used in the subsequent step without purification [15,101].

**Scheme 83:**
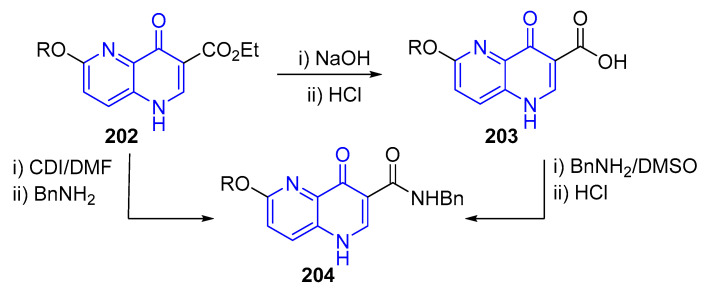
Synthesis of 1,5-naphthyridine carboxylic acids **203** and carboxamides **204**.

1,5-Naphthyridine-3-carboxylates **202** were hydrolysed to prepare the corresponding 1,5-naphthyridine-3-carboxylic acid derivatives **203** (Scheme 83) [22]. On the other hand, from either carboxylate **202** or carboxylic acid **203**, 6-ethoxy-4-oxo-1,4-dihydro-1,5-naphthyridine-3-carboxylic benzylamide **204** was obtained. In this context, Ripin et al. described a multikilogram scale synthesis of **204** [23].

**Scheme 84:**
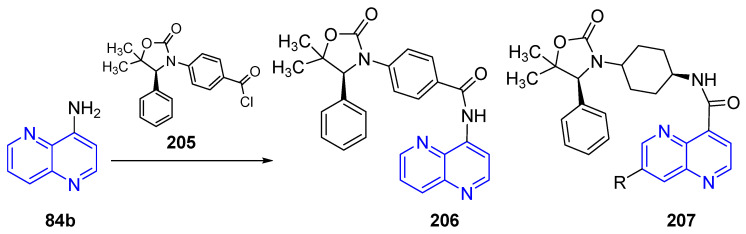
Preparation of functionalized amides **206** and **207**.

Structure-based design and molecular modeling has been used for the preparation of 1,5-naphthyridine with an amide substituent in their structure. The synthesis of the naphthyridine amide derivative **206** was realized by the reaction of an acyl chloride **205** with 1,5-naphthyridine-4-amine **84b** (previously prepared, vide supra, Scheme 31) in high yield (Scheme 84). Analogues, but “reversed” 1,5-naphthyridine amide derivatives **207** were prepared by a standard peptide coupling conditions between 1,5-naphthyridine carboxylic acid and an amine derivative (Scheme 84) [102].

**Scheme 85:**
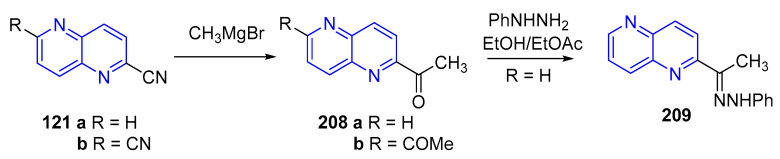
Synthesis of ketones **208** and hydrazone **209** from nitriles **121**.

1,5-Naphthyridine derivatives have been synthesized as new linkers for the construction of bridging ligands. These derivatives were prepared from a 1,5-naphthyridine to give the corresponding cyano 1,5-naphthyridine derivatives **121** (previously prepared, vide supra, Scheme 45), then these compounds were reacted with methyl magnesium bromide to afford ketones **208** (Scheme 85) [74]. The phenylhydrazone **209** derived from 2-acetyl-1,5-naphthyridine **208a** was obtained by the reaction with phenylhydrazine (Scheme 85) [103].

**Scheme 86:**
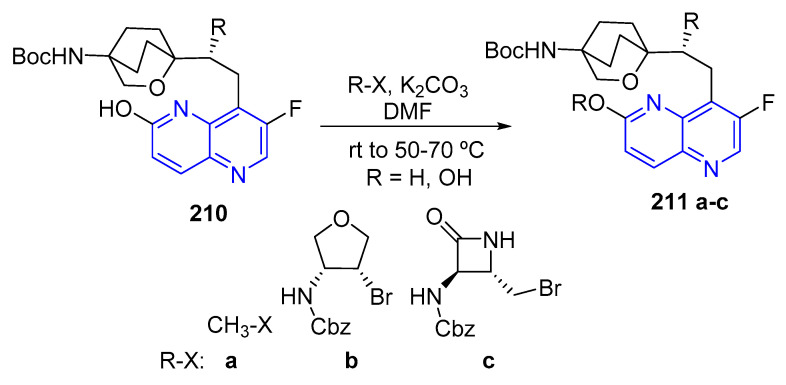
*O*-Alkylation of hydroxyl-1,5-naphthyridines.

On the other hand, some C-2 O-alkylated 1,5-naphthyridine oxabicyclooctane-linked compounds **211** were selectively synthesized by the alkylation of 2-hydroxy-naphthyridyl intermediate **210** with appropriately protected alkyl bromides, methyl bromide or more complexes to control quirality, in the presence of a base (K_2_CO_3_) (Scheme 86) [104].

**Scheme 87:**
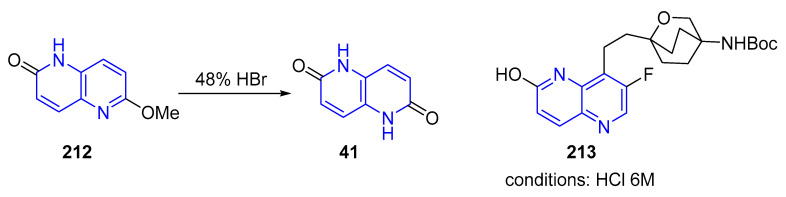
Acid *O*-demethylation of methoxy naphthyridines to obtain compounds **41** and **213**.

In contrast, the dealkylation of the ether moiety was also reported. For example, a *O*-demethylation of the ether group (OMe) linked to the naphthyridine ring **212** was performed with hydrogen bromide to obtain the corresponding 1,5-dihydro-1,5-naphthyridine-2,6-dione **41** (previously prepared, vide supra, Scheme 13), which was subjected to further functionalization (Scheme 87) [37]. Likewise, a similar strategy with aqueous HCl (6M) was used to demethylate the methoxy group at C-2 to give the 1,5-naphthyridine **213** [53].

**Scheme 88:**
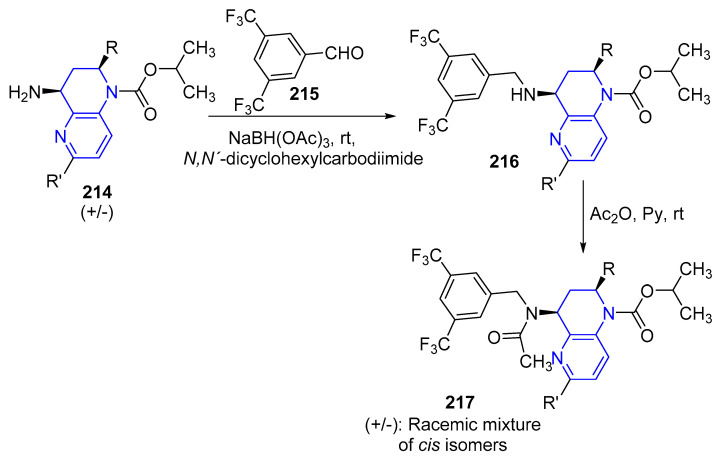
*N*-Functionalizations of compounds **214** to obtain derivatives **217**.

Previously prepared amino intermediates **214** were derivatized by reductive amination with *bis*(trifluoromethyl) benzaldehyde **215** to give derivatives *N*-benzyl amino derivatives **216** and by a further acetylation to afford the corresponding 1,5-tetrahydronaphthyridines **217** (Scheme 88) [47].

**Scheme 89:**
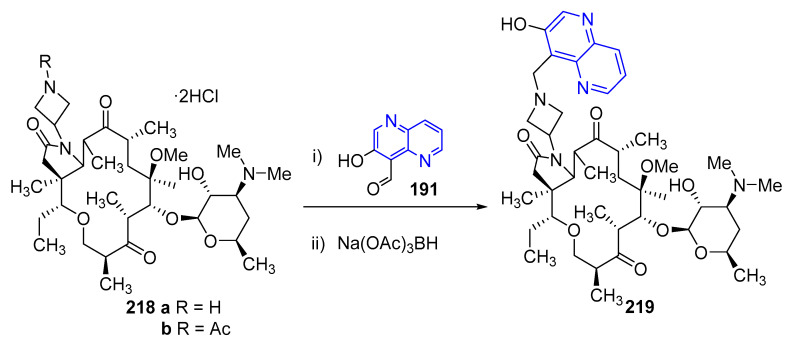
Reductive amination of compounds **218** to obtain derivative **219**.

The scale-up synthesis of the antibacterial azetidinyl ketolide **219** was achieved by a reductive amination between the 1,5-naphthyridine aldehyde **191** (previously prepared, vide supra, Scheme 78) and some clarithromycin derivatives **218** (Scheme 89) [13].

**Scheme 90:**
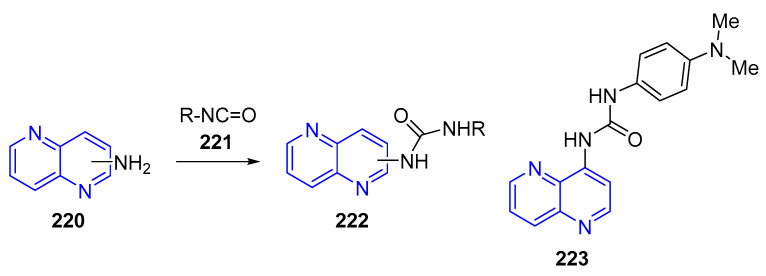
Synthesis of functionalized urea derivatives **222** and **223**.

In the synthesis of 1,5-naphthyridin-2-yl ureas **222** and **223**, azaheterocyclic amines **220** were reacted with various isocyanates **221**, in pyridine at reflux or in two steps in the presence of triphosgene, trimethylamine and then adding the desired amine (Scheme 90) [10,32].

**Scheme 91:**
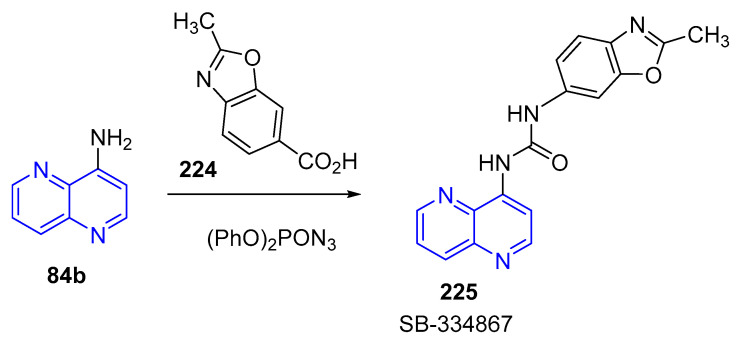
Preparation of diarylurea SB-334867.

In the same context, a series of diarylurea analogues of SB-334867 were obtained from 4-amino 1,5-naphthyridine **84b** (previously prepared, vide supra, Scheme 31) with the appropriate 4-nitrophenylisocyanate followed by reduction of the nitro group to the corresponding amine and a reductive alkylation to obtain **225** (Scheme 91) [105]. Lewin et al. reported a new method for the preparation of 1-(2-methylbenzoxazol-6-yl)-3-(1,5-naphthyridin-4-yl) urea **225** (SB-334867, Scheme 91) containing a very labile 2-methylbenzoxazole ring system, from 4-amino-1,5-naphthyridine **84b** involving a coupling reaction with **224**. The stability and the solubility of the compound was studied and it was found to be stable in DMSO for at least one year [64].

**Scheme 92:**
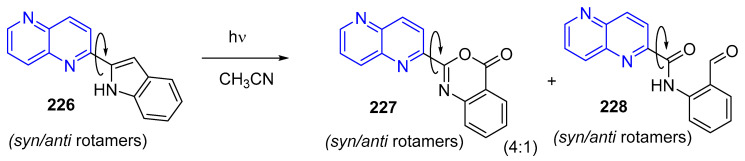
Photochemical addition of acetonitrile to naphthyridine **226.**

1,5-Naphthyridines substituted at the position 2 with an indol, 2-(1′*H*-indol-2′-yl)-1,5-naphthyridine **226**, underwent phototransformation upon 365 nm irradiation in acetonitrile at ambient temperature in the presence of air [106]. The experimental results, combined with the simulations of electronic and infrared (IR) spectra showed two photoproducts, the 2-(1,5-naphthyridin-2-yl)-4*H*-3,1-benzoxazin-4-one **227** as major product and *N*-(2-formylphenyl)-1,5-naphthyridine-2-carboxamide **228** as minor (Scheme 92). The results of this study can help in developing an efficient and easy method for the synthesis of molecules with a benzoxazinone structure.

### 3.6. Formation of Metal Complexes

The 1,5-naphthyridine compounds, when used as ligands in metal complexes, comprise two *N*-donor atoms, which, for geometric reasons, cannot bind to the same metal atom. In addition, when 1,5-naphthyridines form complexes with transition metals, they may behave either as mono or as bidentate ligands. In this section, coordination complexes will be classified by the nature of metal according to their appearance in the periodic table.

**Scheme 93:**
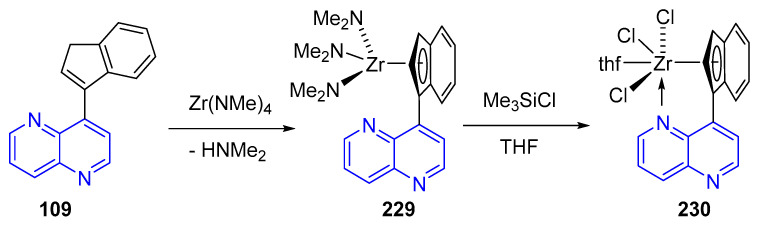
Preparation of zirconium complexes **229** and **230**.

For example, in 1,5-naphthyridin-8-yl-functionalized cyclopentadiene (Cp) and indene (Ind) ligands, the N5 is in a position to activate potential ligand-coupling reactions, while N1 can coordinate to a metal in a manner analogous to 8-quinolyl-functionalized Cp and Ind ligands. In this sense, the formation of a 1,5-naphthyridine complex with Zr(NMe)_4_ was studied [62]. Both components reacted quickly at room temperature with formation of complex **229** as the only product together with free HNMe_2_. This complex could be converted to the stable chloro derivative complex **230** by the addition of Me_3_SiCl (Scheme 93).

**Scheme 94:**
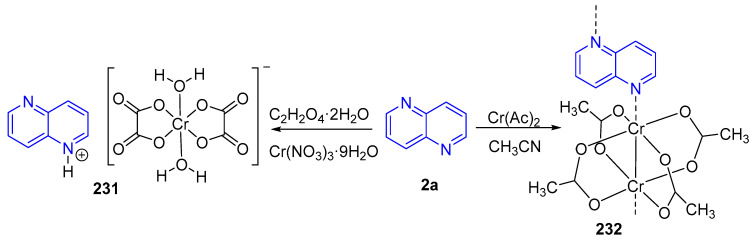
Formation of chromium complexes **231** and **232**.

A chromium (III) complex 1,5-naphthyridine *trans*-diaquadioxalatochromate(III) dehydrate **231**, was synthesized by self-assembly of chromium(III) nitrate with oxalic acid and 1,5-naphthyridine **2a** (previously prepared, vide supra, Scheme 1) to obtain violet crystals suitable for X-ray diffraction (Scheme 94) [107]. The structures were obtained as organic layers, built up by the 1,5-naphthyridine cations, arranged in a sandwich between the layers of the [Cr(C_2_O_4_)_2_(H_2_O)_2_]-complex anion and free water molecules. Also from 1,5-naphthyridine, paddlewheel-type dichromium(II,II) tetraacetate complex **232** was successfully prepared by liquid−liquid interdiffusion with chromium(II) acetate (Cr(Ac)_2_) in acetonitrile with a layer of pure CH_3_CN separating the two phases in a narrow glass tube (Scheme 94) [108].

**Scheme 95:**
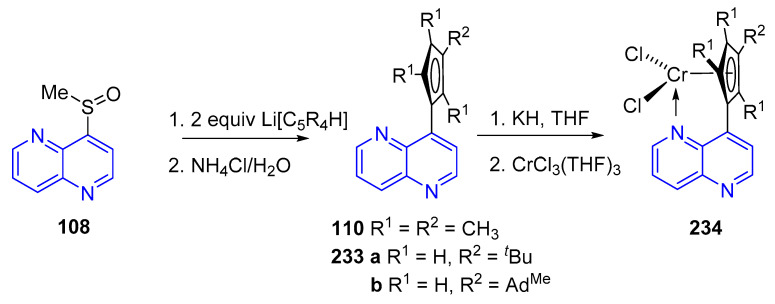
Formation of chromium(III) complex **234**.

Starting from functionalized cyclopentadienyl-1,5-naphthyridines **110** (previously prepared, vide supra, Scheme 40) and **233a**,**b**, a variety of chromium(III) half-sandwich complexes **234** have been obtained (Scheme 95) [109].

**Scheme 96:**
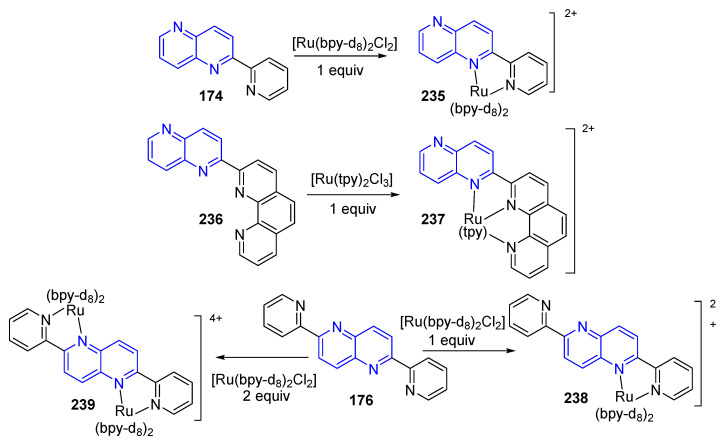
Preparation of heteroleptic ruthenium(II) complexes **235**, **237**-**239**.

When using a 1,5-naphthyridine nucleus with *N*-heterocyclic substituent as a coordinating entity in a reaction with [Ru(bpy-d_8_)_2_Cl_2_], a wide variety of ruthenium complexes with bidentate and tridentate ligands, have been obtained [74]. For example, the treatment of 2-(pyridin-2-yl)-1,5-naphthyridine **174** (previously prepared, vide supra, Scheme 73) with 1 equiv. of [Ru(bpy-d_8_)_2_Cl_2_] furnished the corresponding heteroleptic Ru(II) complex **235** (Scheme 96), while the treatment of 2-(1,5-naphthyridin-2-yl)-1,10-phenanthroline **236** with 1 equiv. of [Ru(tpy)_2_Cl_3_] afforded the corresponding heteroleptic Ru(II) complex **237** containing a tridentate ligand (Scheme 96). On the other hand, many of these ligands, as for example compound **176** (previously prepared, vide supra, Scheme 73), posses bisubstituted 1,5-naphthyridine scaffold as a linker between the two equivalent coordinating sites giving rise to both *bis*-bidentate and *bis*-tridentate ligands. When reacting with 2 equiv. of [Ru(bpy-d_8_)_2_Cl_2_] or [Ru(tpy)_2_Cl_3_], complexes **238** and **239** can be obtained, respectively (Scheme 96). The efficiency of the 1,5-naphthyridine linker has been compared to the previously studied pyrazine linker, and the latter is found to promote better communication between the two bound metals.

**Scheme 97:**
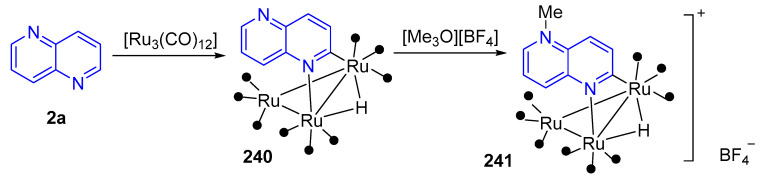
Formation of triruthenium cluster compounds **240**-**241**.

Cabeza et al. reported the synthesis of a triruthenium cluster compound containing cationic 1,5-naphthyridine ligand **241** by treatment of [Ru_3_(CO)_12_] with the neutral *N*-heterocycle **2a** and subsequent methylation of the product **240** with trimethyloxonium tetrafluoroborate as the methylating reagent (Scheme 97) [110].

**Scheme 98:**
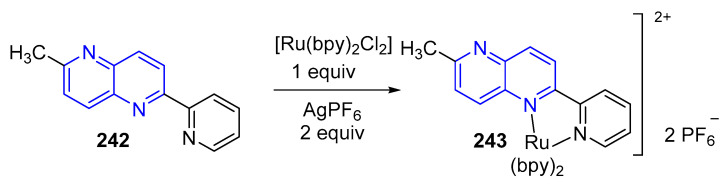
Synthesis of heteroleptic ruthenium(II) complex **243**.

The heteroleptic ruthenium(II) complex, [Ru(bpy)_2_(Me-pn)](PF_6_)_2_ **243**, 2-methyl-6-(pyridin-2-yl)-1,5-naphthyridine **242** ligand, has been prepared under photochemical reduction conditions (Scheme 98) [111].

**Scheme 99:**
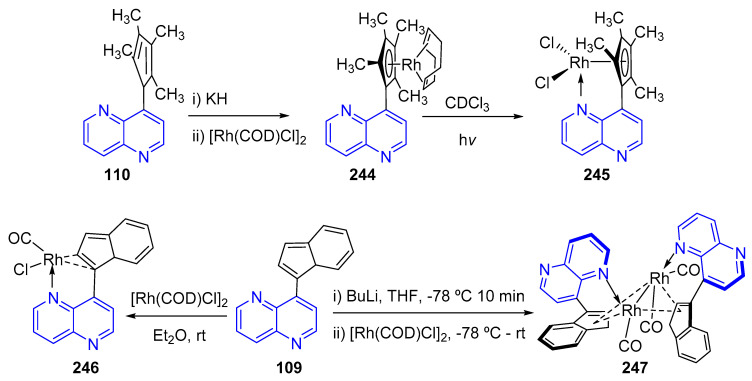
Formation of rhodium complexes **244**-**247**.

The synthesis of rhodium complexes of 1,5-naphthyridines was accomplished by the coupling of a *N*-donor-functionalized indenyl and cyclopentadienyl-1,5-naphthyridine unit (Scheme 99) [62]. The synthesis of the red Rh(III) complexes, **245** and **246** was achieved by reaction of the deprotonated ligand **110** or **109** (previously prepared, vide supra, Scheme 40) with the rhodium precursor, di-μ-chlorodicyclooctadienyldirhodium. For the synthesis of the binuclear derivative **247**, BuLi was used before the rhodium was added.

**Scheme 100:**
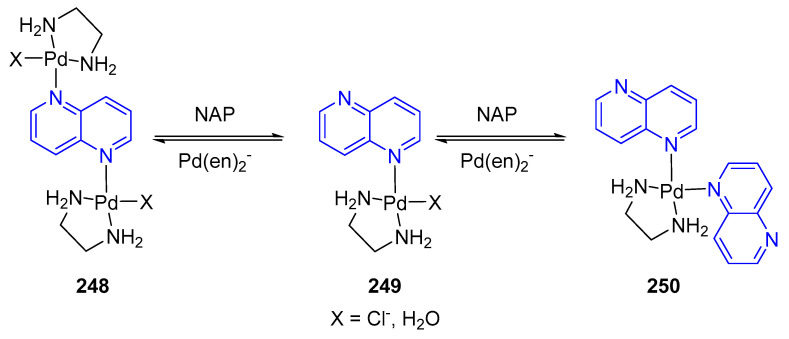
Interconversion of palladium(II) complexes derived from 1,5-naphthyridine in aqueos solutions.

Walmsley et al. investigated the interactions of 1,5-naphthyridine with Pd(II) in aqueous solution by 1D and 2D ^1^H NMR spectroscopy and potentiometric titration [112]. In this study, they observed that 1,5-naphthyridine acts as a ligand coordinating to Pd(II), in either [Pd(en)(H_2_O)_2_](NO_3_)_2_ or Pd(en)Cl_2_ form, in aqueous solutions to produce the 2:1 and 1:1 complexes, compounds **248** and **249** respectively (Scheme 100), as well as a 1:2 oligomeric species **250**. Some other different complexes were observed when pH was modified (not indicated) (Scheme 100).

**Scheme 101:**
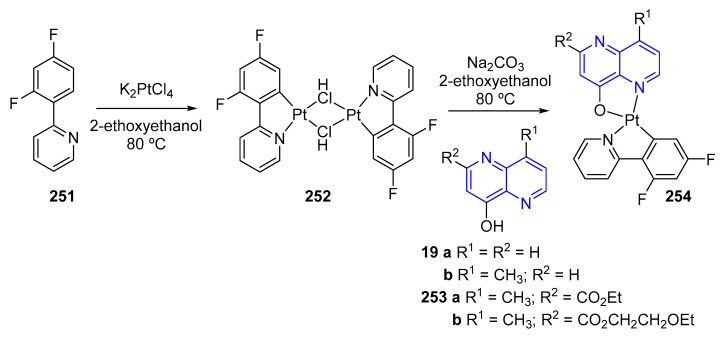
Formation of heteroleptic platinum(II) complexes **254**.

Heteroleptic platinum(II) complexes bearing two ligands, hydroxynaphthyridine and 2-(2,4-difluorophenyl)pyridine, were synthesized in two steps [19,113]. The first ligand **251** was attached by treatment with K_2_PtCl_4_ to form platinum dichloride-bridged dimer **252**, followed by reaction with naphthyridinol derivatives **19a**,**b** (previously prepared, vide supra, Scheme 6) or **253a**,**b** as second ligand in 2-ethoxyethanol to yield complexes **254** (Scheme 101).

**Scheme 102:**
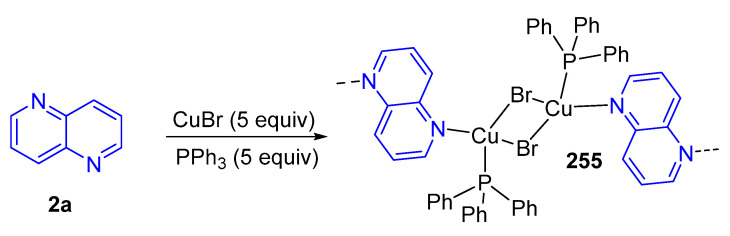
Synthesis of copper(I) mixed-ligand complex **255**.

Copper(I) mixed-ligand complexes, [Cu_2_(μ-Br)_2_(PPh_3_)](L)_n_ **255**, including a 1,5-naphthyridine ring were prepared by reaction with 5 equiv. of CuBr and 5 equiv. of PPh_3_ and obtained as crystalline materials (Scheme 102) [114]. Bidentate ligands, such as 1,5-naphthyridine **2a** (previously prepared, vide supra, Scheme 1), formed infinite chain complexes **255** with the ligands bridging the dimeric units.

**Scheme 103:**
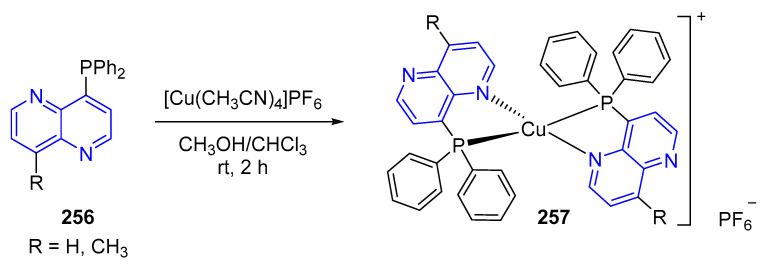
Preparation of homoleptic copper(I) complex **257**.

Later on, the homoleptic Cu(I) complexes **257** were obtained by the reaction of 4-diphenylphosphino-1,5-naphthyridine ligands **256** with [Cu(CH_3_CN)_4_]PF_6_ in CH_3_OH/CHCl_3_ (Scheme 103) [115].

When 1,5-naphthyridine dimers were used, copper(II) complexes were prepared with cooper(II) nitrate producing a discrete mononuclear complex in which the copper atom is chelated to the central internal nitrogens, demonstrating that the ligand could act in a variety of coordination modes [63]. In this work, copper complexes, as well as silver and palladium complexes were obtained in a similar way. Polynuclear silver(I) complexes with 1,5-naphthyridines were also synthesized by the reaction of the three AgX salts (X = NO_3_^−^, CF_3_COO^−^ and CF_3_SO_3_^−^) and 1,5-naphthyridine in ethanol at room temperature [116].

Regarding gold complexes, Pyykko et al. reported a computational study of some gold complexes of 1,5-naphthyridines [117]. In this case, it is indicated that gold atom can act as an intermolecular glue, coupling(hetero)aromatic rings, typically through C–Au←N bonds.

**Scheme 104:**
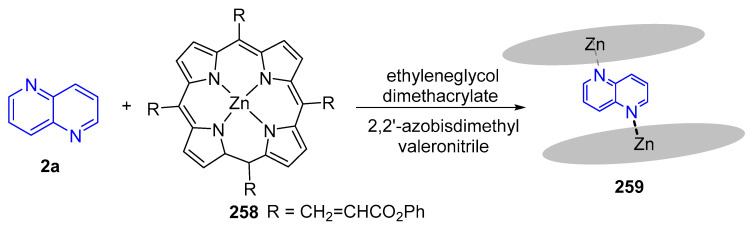
Preparation of complex **259** with a 1,5-naphthyridine bidentate ligand.

Matsui et al. used 1,5-naphthyridines complexed with zinc as a template molecule for the polymerization of porphyrin monomer units **259** (Scheme 104), in a bottom-up architecture construction of supramolecules consisting of two face-to-face porphyrins [118]. A schematic representation of a face-to-face arrangement of two porphyrins is shown in Scheme 104. In this case, 1,5-naphthyridine **2a** (previously prepared, vide supra, Scheme 1) acts as a linker through bidentate zinc complexation in intermolecular interactions of porphyrins.

Moreover, also ML_2×2_ type Zn(II), Co(II) and Ni(II) complexes were prepared from 1,5-naphthyridines [119].

**Scheme 105:**
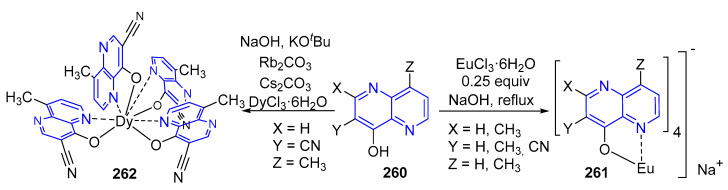
Formation of europium complexes **261** and dysprosium complex **262**.

A series of europium complexes with structurally rigid 4-hydroxy-1,5-naphthyridine ligands were developed [120]. The Eu^3+^ ion owns large radius and thus usually requires high coordination number of 8 or 9 to stabilize the coordination environment [121]. Therefore, when using bidentate ionic ligands, such as 4-hydroxy-1,5-naphthyridines **260**, to construct Eu^3+^ complexes, there are two typical synthetic strategies, ratio 3:1 (ionic ligands versus Eu^3+^ ions) plus one or more additional neutral ligands, or ratio 4:1 without any other neutral ligands. The latter strategy was adopted by Bian et al. and the 4-hydroxy-1,5-naphthyridine-based europium complexes **261** were synthesized with the structure of Na^+^ [Eu(NDs)_4_]^−^, where each Eu^3+^ ion is chelated by four 1,5-naphthyridine ligands, and one Na^+^ ion was incorporated as the counter ion (Scheme 105). For the synthesis, a mixture of ND-ligand and NaOH in ethanol was refluxed for 10 min and the suspension was then poured into a solution of EuCl_3_·6H_2_O and stirred overnight at reflux temperature. The same group prepared Eu(III) complexes with tridentate ligands, such as 8-hydroxy-1,5-naphthyridine-2-carboxylic acid [28] and 6-(diphenylphosphoryl)-4-hydroxy-2-methyl-1,5-naphthyridine [29] by the “self-assembly” of the ligand, NaOH, and EuCl_3_·6H_2_O in a ratio of 3:3:1 in methanol.

**Scheme 106:**
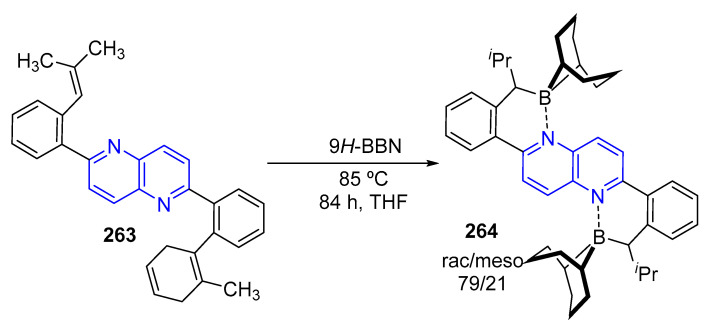
Hydroboration reaction of 1,5-napthyridines to obtain borane **264**.

In addition, a series of dysprosium(III) ion coordinated by four chelated naphthyridine-like ligands (L = 4-hydroxy-8-methyl-1,5-naphthyridine-3-carbonitrile **260**, X = H, Y = CN, Z = CH_3_) and an alkali metal ion (A = Na, K, Rb, Cs) **262** were synthesized and characterized (Scheme 105) [122]. Not only transition metals, but also non-transition metals have been used for the preparation of 1,5-naphthyridine complexes. The hydroboration of 1,5-naphthyridine-based precursor **263** (Scheme 106) with 9*H*-9-borabicyclo[3.3.1]nonane (9H-BBN) afforded π-conjugated N→B-ladder borane **264** [123].

**Scheme 107:**
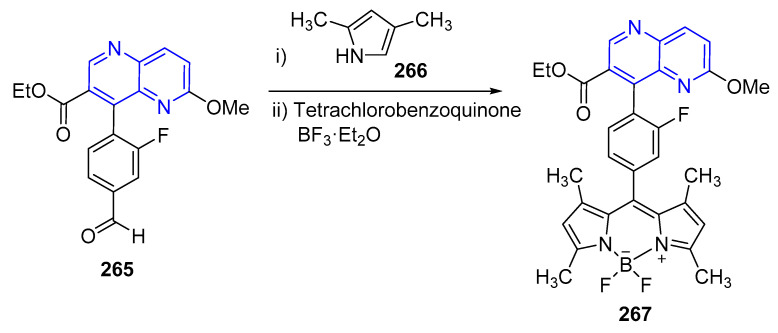
Preparation of BODIPY derivative **267**.

Boron-dipyrromethene (BODIPY) derivatives containing a naphthyridine ring were designed from 8-(4-formylaryl)-2-methoxynaphthyridine **265** in high yield. A Knoevenagel reaction was done with 2,4-dimethylpyrrole **266** followed by treatment with BF_3_·Et_2_O to produce BODIPY compound **267**. Further functionalization of the pyrrole rings may afford new BODIPY derivatives (Scheme 107) [95].

**Scheme 108:**
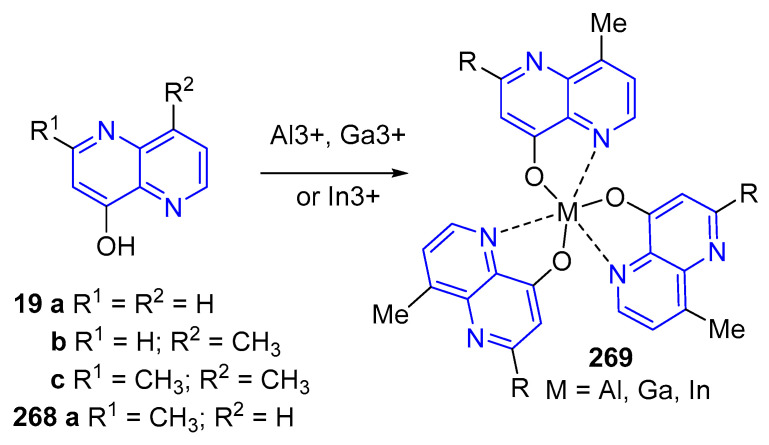
Synthesis of aluminium, gallium and indium complexes **269**.

Some research groups were involved in the study of group III metal chelates from 4-hydroxy-1,5-naphthyridine derivatives. Chen, Wu et al. reported the synthesis of aluminium, gallium and indium chelates of 4-hydroxy-1,5-naphthyridines **269** (Scheme 108) [31]. Due to the differing reactivity of the metal starting materials and the different solubility of the metal chelate products, various reaction conditions and the isolation/purification method were adopted in the final metal chelation reactions, and aluminium triisopropoxide, gallium chloride and indium chloride were used for the introduction of metals.

On the other hand, Wang et al. reported the DFT/B3LYP/6-31G(d) and TD-DFT calculations for the theoretical generation of group III metal chelates of 4-hydroxy-8-methyl-1,5-naphthyridine [124]. Calculation results are consistent with the experimental data previously reported by Chen, Wu et al. One year later, Lee et al. published another theoretical study with aluminium chelates of 4-hydroxy-1,5-naphthyridines [125].

## 4. Properties of 1,5-Naphthyridines

The aromaticity of 1,5-naphthyridine ring has been studied with magnetic criteria, magnetic susceptibility isotropic and nucleus-independent chemical shifts (NICS), calculated at the BLYP/6-31G(d) level of theory. According to this study, the magnetic susceptibility and not NICS is a reliable criteria in their case [126]. In another study, the aromaticity of quinolines, naphthyridines and phenanthrolines was evaluated [59]. According to the results, quinoline is the most aromatic substrate, followed by naphthyridine and then phenanthroline, and substantial influence of the position of the nitrogen atoms of the (poly)aromatic compounds protonation reaction was observed. Recently, stacking interactions involving Asp−Arg and Glu−Arg salt-bridges with aromatic 1,5-naphthyridines have been studied using robust ab initio methods. The guanidinium of Arg is consistently positioned, such that two nitrogen atoms are located above the ring centroids and the salt-bridge dipole is aligned with the electric field above the ring [127].

**Scheme 109:**
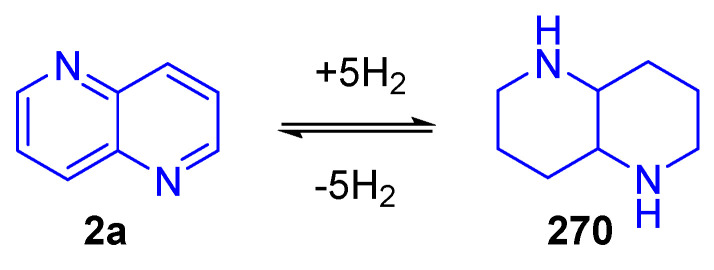
1,5-Naphthyridine as organic hydrogen carrier.

These heterocyclic compounds could be considered as organic hydrogen carriers. A high level ab initio quantum chemistry calculations suggested that the release of H_2_ (when passing from aromatic 1,5-naphthyiridine to perhydro-1,5-naphthyiridine) was greatly favored thermodynamically and the corresponding perhydro nitrogenated heterocycles possess high hydrogen storage capacity [128]. The 1,5-naphthyridine compound **2a** (previously prepared, vide supra, Scheme 1) exhibits 3.64 kcal mol^−1^ and spontaneously dehydrogenates (Scheme 109).

DFT calculations have been also applied to calculate proton affinities, polarizabilities, and some ionization energies and atomic and ring neutral bond orbital (NBO) for some polycyclic aromatic nitrogen heterocyclics (PANHs), including 1,5-naphthyridine [129]. In 1,5-naphthyridines, which contain two-nitrogen atoms in their skeleton, the charge delocalization weakens the mutual effects of the nitrogens and therefor the proton affinities decrease.

Static quantum chemical calculations and first principles molecular dynamics (FPMD) simulations were used to examine the behavior of 1,5-naphthyridine-2,6-diol, which indicated that the N−H interactions were sufficiently strong to induce proton transfer processes that caused this molecular species to exist preferentially as a single layer [130].

In addition, in some other theoretical studies the molecular recognition was predicted by the hydrogen bonding (HB) in order to design biologically active molecules. In this case, 1,5-naphthyridine showed HB basicity of nitrogen lower than the measured values for quinoline and isoquinoline [131].

Hexadecane/water and 1-octanol/water partition coefficients have been measured for 1,5-naphthyridine ring and ∆logP values have been determined to be derived for a number of hydrogen bond acceptors that are neutral under normal physiological conditions. It has been shown that minimized electrostatic potential is a useful descriptor to predict the contribution of hydrogen bond acceptors to ΔlogP [132].

**Scheme 110:**
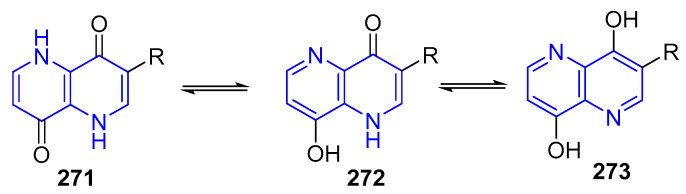
Theoretical tautomeric studies of 4,8-dioxygenated 1,5-naphthyridines.

On the other hand, theoretical studies of the tautomerism of 4,8-dioxygenated 1,5-naphthyridine have been done in the gas phase and solution phase using polarisable continuum method at the B3LYP/6-311++G level [133]. In the gas phase all **271** form were more stable than the others (**271** > **273** > **272**). With increasing polarity, total energy of all compounds were more negative. The charges on all five positions were affected by substituents and solvents. In addition, a regular variation was found with increasing of dielectric constant (Scheme 110).

## 5. Applications of 1,5-Naphthyridines

Along with the synthesis of 1,5-naphthyridines indicated in previous sections, a wide range of applications has been reported many of them being related to the biological activity of the compounds and others to their physical properties.

### 5.1. Biological Activity

Among the biological activities reported for 1,5-naphthyiridine derivatives antiproliferative, antibacterial, antiparasitic, antiviral properties, in most cases enzyme inhibitor, anti-inflammatory activity or influence on the cardiovascular or central nervous system (CNS) diseases have been to mentioned.

#### 5.1.1. Antiproliferative Activity

**Figure 6:**
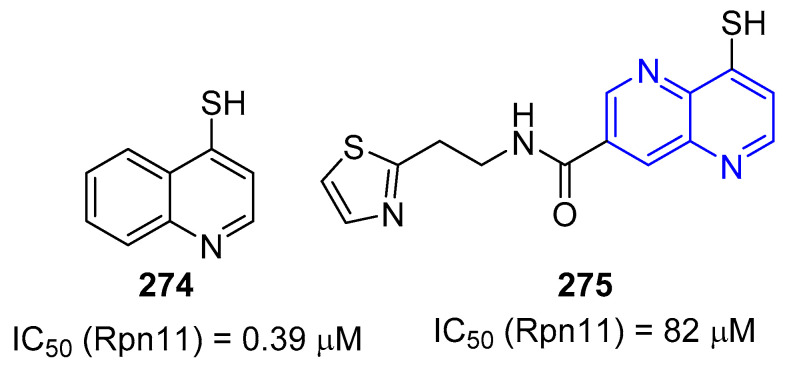
Inhibition of Rpn11.

1,5-Naphthyridine **275** (Figure 6) prepared by a fragment-based drug discovery (FBDD) approach as an analogue of lead compound **274**, showed not only micromolar IC_50_ values (~82 μM) as Rpn11-selective inhibitor (proteasome inhibitor) but also presented also cytotoxicity toward cancer cell lines [34]. Inhibition of Rpn11 may lead to preferential apoptosis of neoplastic cells because these cells are thought to have a higher dependency on proteasome-dependent protein quality control compared to normal cells.

**Figure 7:**
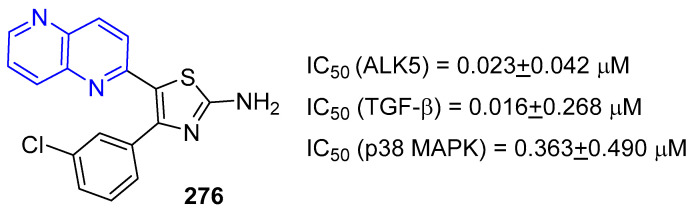
Inhibition of ALK5, on enzyme and TGF-α-dependent cells.

Significantly, protein kinases are one of the most targeted group; some kinase inhibitors have been approved by FDA for treatment of cancer. In 2004 Gellibert et al. reported the selectivity of 1,5-naphthyridine derivatives against p38 MAP kinase. In this case, 1,5-naphthyridin-4-yl-substituted aminothiazoles, such as compound **276** in Figure 7, and pyrazoles showed potent inhibition of ALK5 on both the enzyme and the TGF-*α*-dependent cellular assays. Transforming growth factor (TGF-α) is a pluripotent cytokine involved in a variety of biological processes such as development, cell growth, differentiation, cell adhesion, migration, extracellular matrix deposition, fibrosis and immune response regulation. Indeed, TGF-*α* overexpression has been implicated in multiple disease states including pulmonary fibrosis, liver fibrosis, renal glomerulosclerosis, and cancer [15]. Also a pyrazol substituted 1,5-naphthyridine showed inhibition of ALK5 on the TGF-β type I receptor [134].

**Figure 8:**
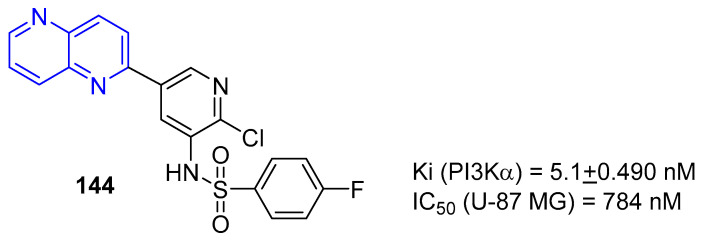
PI3Kα inhibitory activity and cytotoxicity on U-87 MG cells.

In 2011, Nishimura et al. prepared 1,5-naphthyridine derivative **144** (previously prepared, vide supra, Scheme 62) and studied the PI3Kα inhibitory activity and determined the IC_50_ values using U-87 MG human brain cells (Figure 8) [73]. Therapeutics targeting the PI3K pathway may have utility for the treatment of cancer, since this target plays an important role in cell growth and survival and it is dysregulated in many human cancers. Even the 1,5-naphthyridine derivative **144** shows a good enzyme inhibitory activity (Ki value < 10 nM) they decided not to focuse on this scaffold because the additional nitrogen at position 5 seemed not to offer significant advantages over quinolines.

**Figure 9:**
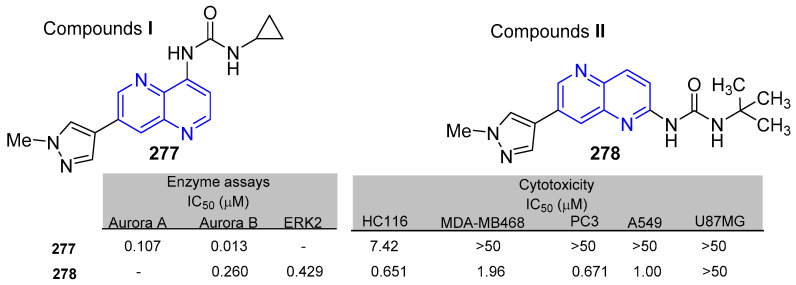
Biological activity on Aurora kinases A and B and cytotoxicity values.

In 2014, new chemical entities of biological interest, such as 7-aryl-1,5-naphthyridin-4-ylureas appeared showing excellent inhibitory activities toward Aurora kinases A and B for the treatment of malignant diseases based on pathological cell proliferation [32]. In this study, the most active compound, 1-cyclopropyl-3-[7-(1-methyl-1*H*-pyrazol-4-yl)-1,5-naphthyridin-4-yl]urea **277** (Figure 9), displayed IC_50_ values of 107 and 13 nM against Aurora kinases A, and B, respectively. However, the compound showed low antiproliferative activity when tested toward tumor cell lines. Then, this group considered modifying the position of the urea appendage on 1,5-naphthyridine ring from position 4 to 2 obtaining the 7-aryl-1,5-naphthyridin-2-ylureas, as compound **278** (Figure 9) [10]. This modification maintained inhibition of the Aurora B kinase, but also led to the inhibition of the Raf/MEK/ERK signaling pathway. In this sense, several analogues were active at micromolar and submicromolar range against ERK2 and Aurora B, associated with very promising antiproliferative activity.

**Figure 10:**
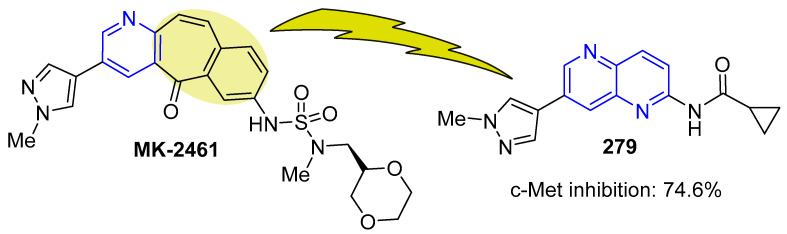
Inhibition of c-Met kinase activity.

The compound MK-2461 was developed by Merck and identified as an ATP-competitive c-Met inhibitor. C-Met is the high affinity receptor tyrosine kinase (RTK) that binds with the hepatocyte growth factor (HGF). Based on scaffold Wu et al. reported the synthesis and biological study of a series of 1,5-naphthyridine derivatives **279** in which the heptatomic ring is omitted compared with MK-2461 (Figure 10) [9]. The compounds were tested at a single concentration of 10 μM and defined as effective inhibiting over half of the c-Met kinase at that concentration. Noteworthy, compound **279** with a cyclopropyl group proved to by the most active presenting the highest (74.6%) inhibition of c-Met kinase. In addition, compounds were also tested in vitro for anti-tumor activities against cervix (Hela) and lung (A549) human cancer cell lines.

**Figure 11:**
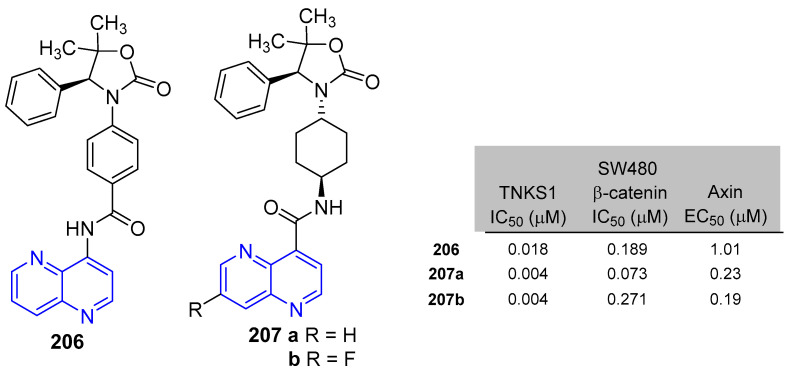
Inhibition of tankyrase activity.

In 2013, orally bioavailable amide substituted 1,5-naphthyridines, as compound **206** (previously prepared, vide supra, Scheme 84), were reported as potent and selective tankyrase inhibitors (Figure 11). The inhibition of tankyrase may reduce the levels of β-catenin-mediated transcription and inhibit tumorigenesis, offering a novel approach towards the treatment of colorectal cancer. In order to further improve the potency via the H-bond donor properties of the hydrogen at position 2, naphthyridine derivative **207a** (R = H) was prepared. Although, improved enzyme and cellular potency, but poor oral exposure in mice, were achieved. Further blocking the sites of potential oxidative metabolism yielded fluorinated analogue **207b** (R = F), which featured retained potency, with improved solubility and improvements in oral exposure (Figure 11) [102].

**Figure 12:**
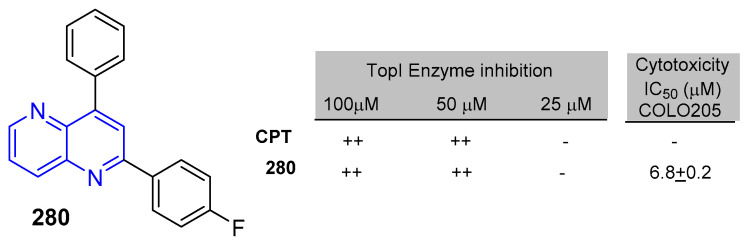
Inhibition of topoisomerase I activity.

DNA topoisomerases unravel turns in DNA that occur as a result of DNA transcription and replication. A series of 4-phenyl-1,5-naphthyridines derivatives exhibited inhibitory effect on topoisomerase I comparable to those observed for the natural inhibitor camptothecin (CPT), and it was the first time that simple 1,5-naphthyridines were tested as topoisomerase I inhibitors [45]. Among the prepared derivatives, aromatized naphthyridines were selected for the evaluation of their therapeutic effect against a human colon cancer cell line (COLO205). The fluorinated derivative **280** (Figure 12) was the most cytotoxic, showing good enzyme inhibition and IC_50_ values close to CPT.

**Figure 13:**
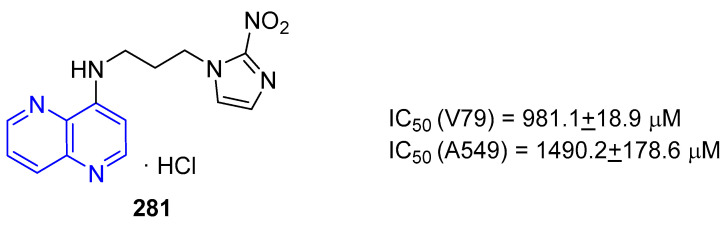
Cytotoxicity against cancer cells.

One point for the effectiveness of cancer therapy depends on the evolution of DNA in the cell. Therefore, those compounds that can directly affect DNA, its replication or repair, are very interesting in the treatment of cancer. In 2009, a nitroimidazol 1,5-naphthyridine hydrochloride derivative **281** (Figure 13) was synthesized and evaluated as DNA binder and its cytotoxicity, radiosensitization and interaction with chemotherapeutic agents studied. Despite this compound did not show DNA-binding activity and resulted less potent hypoxic cytotoxins than weak DNA-intercalative bioreductives, it can still show good hypoxic selectivity values and interact with radiation/chemoterapeutic agents [18].

Rajković et al. in 2018 synthesized new 1,5-naphthyridine-bridged (1,5-nphe) dinuclear platinum(II) complexes, with the general formula [{Pt(L)Cl}2(μ-1,5-nphe)](ClO_4_)_2_, and evaluated the in vitro cytotoxic activity of these complexes against three tumor cell lines, murine colon carcinoma (CT26), murine mammary carcinoma (4T1) and murine lung cancer (LLC1) and two normal cell lines, murine mesenchymal stem cells (MSC) and human fibroblast (MRC-5) cells [135].

#### 5.1.2. Antibacterial and Antifungal Activity

**Figure 14:**
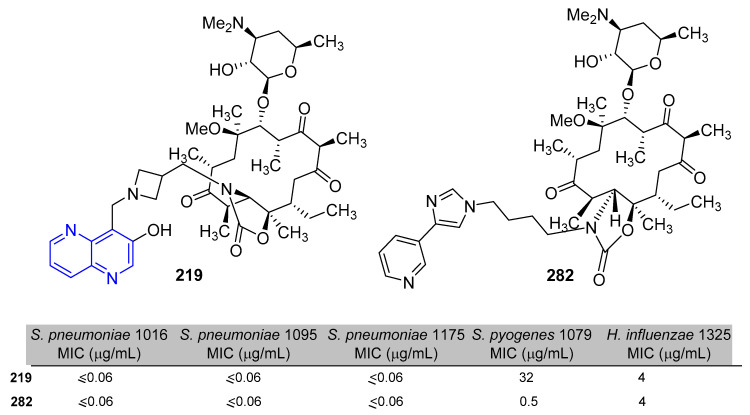
Antibacterial activity of 1,5-naphthyridine-containing macrolides.

Regarding antibacterial activity, macrolide antibiotics with a 1,5-naphthyridine core in their structure present some improvements with resistant respiratory pathogens for the treatment of community-acquired infections [12]. The best results were obtained for the 3-hydroxy-1,5-naphthyridine analogue **219** (previously prepared, vide supra, Scheme 89). This compound is differentiate from telithromycin **282** in both hepatic microsomal extraction ratio and CYP3A time-dependent inactivation, mantaining preclinical in vivo efficacy (Figure 14).

**Figure 15:**
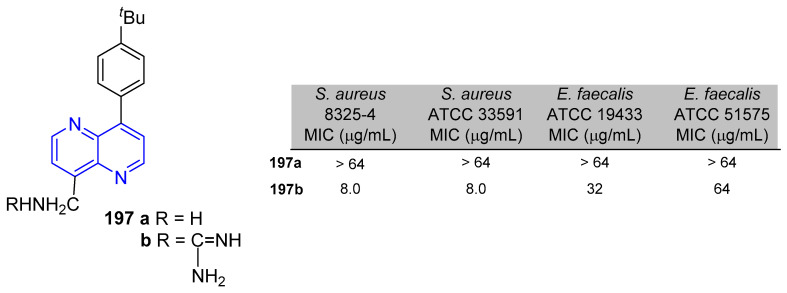
Antibacterial activity of some 1,5-naphthyridines against drug-sentsitive and drug-resistant bacteria.

The development of new therapies against methicillin-resistant *Staphylococcus aureus* (MRSA) is needed to counteract the significant threat that MRSA presents to human health. In this regard, 1,5-naphthyridine derivatives have been evaluated as antibacterial agents against methicillin-sensitive and methicillin-resistant *S. aureus* and vancomycin-sensitive and vancomycin-resistant *Enterococcus faecalis*. While aminomethyl derivative **197a** (previously prepared, vide supra, Scheme 80) did not exhibit significant antibacterial activity, the guanidinomethyl derivative **197b** showed more pronounced antibacterial activity. (Figure 15) [98].

Antibacterial activity of a 1,5-naphthyridines with chromium metal ion and oxalic acid has been evaluated by testing their antagonistic effect against Gram-positive bacteria, *Enterococcus faecalis*, *Staphylococcus aureus*, *Listeria innocua*; and Gram-negative species *Shigella flexneri*, *Escherichia coli*, *Salmonella enterica* and *Pseudomonas aeruginosa* [107]. Antibacterial study confirmed that the 1,5-naphthyridine ligand is biologically active against Gram-negative *S. enterica* bacteria and the chromium(III) complex showed enhanced activity against *L. innocua*, *P. aeruginosa*, and *E. coli* upon coordination. The free 1,5-naphthyridine ligand was found to be biologically inactive against *S. flexneri* and *E. faecalis* bacteria. The coordination of this ligand with the anionic chromium (III) moiety induced appreciable antimicrobial activities against the Gram-negative *S. enterica* bacteria but not towards the Gram-positive *S. aureus* bacteria.

**Figure 16:**
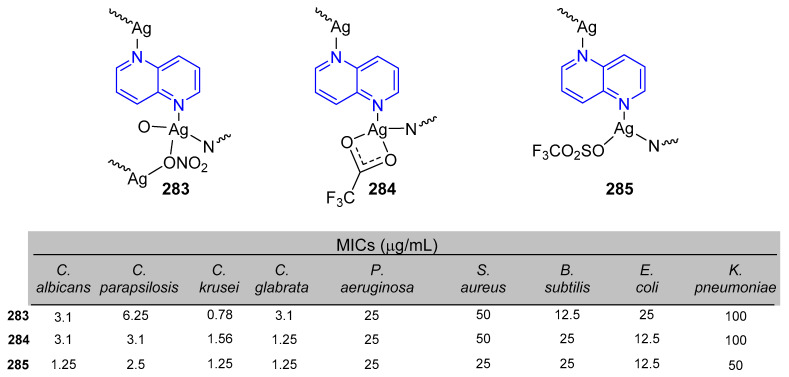
Antibacterial activity of silver complexes.

The antimicrobial efficiency of polynuclear silver(I) complexes with 1,5-naphthyridine **283**–**285** was evaluated against the broad panel of Gram-positive and Gram-negative bacteria and fungi (Figure 16) [116]. The complexes showed good to moderate antibacterial activity with the minimal inhibitory concentration (MIC) values being in the range 12.5–100 μg/mL, while their antifungal activity against the investigated *Candida* spp. was significantly higher (MIC = 0.78–6.25 μg/mL, Figure 16). Moreover, complexes **283** and **284** effectively inhibited *Candida albicans* biofilms formation, while **283** also inhibited the formation of mixed *Candida albicans*/*Pseudomonas aeruginosa* biofilms. Toxicological evaluations on zebrafish (*Danio rerio*) embryos revealed that all silver(I) complexes could be applied as antifungal agents, whereas **285** had the best therapeutic potential showing both the lowest MIC values against the tested *Candida* strains and the non-toxic in vivo response in the zebrafish embryos at these doses.

**Figure 17:**
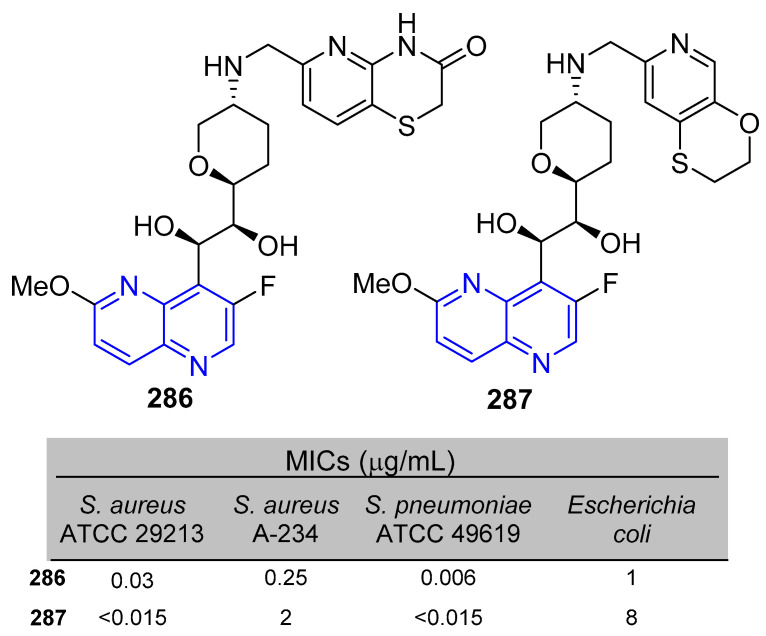
Antibacterial activity of THP-naphthyridine derivatives.

Novel bacterial topoisomerase inhibitors (NBTIs) are new class of broad-spectrum antibacterial agents targeting bacterial DNA gyrase and topoisomerase IV at a site distinct from quinoline binding. Bicyclic aromatic 1,5-naphthyridine derivatives linked to a tetrahydropyran (THP) ring through a *syn*-diol linker showed potent anti-Gram-positive activity [65]. For instance, compound **286** was found to be a dual DNA gyrase-topoisomerase IV inhibitor with broad antibacterial activity and low propensity for spontaneous resistance development, but suffered from high hERG K^+^ channel block (Figure 17). On the other hand, analog 287 displayed lower hERG K^+^ channel block, while retaining potent in vitro antibacterial activity and acceptable frequency for resistance development. Furthermore, 1,5-naphthyridine 287 showed moderate clearance in rat and promising in vivo efficacy against *Staphylococcus aureus* in a murine infection model. These THP-naphthyridine-based bacterial topoisomerase inhibitors display promising properties and deserve further efforts to be eventually transformed into new clinically used antibacterial agents.

**Figure 18:**
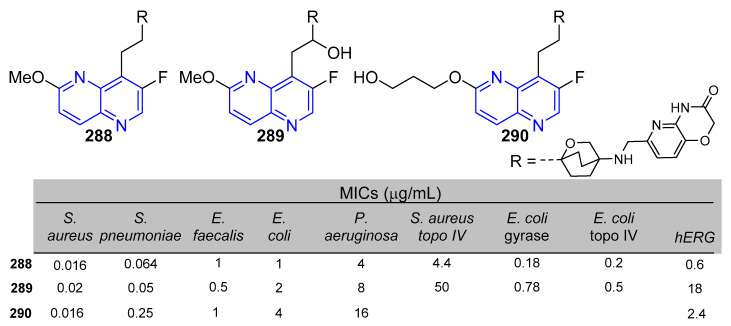
Broad-spectrum antibiotics containing a 1,5-naphthyridine core.

Naphthyridine derivatives, whose structural features consist of three structural motifs: a left hand site bicyclic aromatic heterocycle (a 1,5-naphthyridine), a right hand side aromatic heterocycle connected by a 8-atom central linker (a oxabicyclooctane) placing a basic nitrogen at position 7, present no cross-resistant to known antibiotics and therefore serve as excellent candidates to combat drug-resistant bacteria [25]. Singh et al. evaluated these compounds against a panel of key Gram-positive and Gram-negative strains of bacteria, as well as for hERG activity and for in vivo efficacy in murine model of *Staphylococcus aureus* infection. This type of compound, such as derivative **288**, showed a potent broad-spectrum as antibacterials. NBTIs with reduced off-target activity (hERG IC_50_ > 18 μM) and improved physical properties (Figure 18). On the other hand, hydroxyl substituted derivative **289** was bactericidal, selectively inhibited DNA synthesis, *Staphylococcus aureus* gyrase (IC_50_ = 1.02 μM) and topoisomerase IV (IC_50_ = 10.4 μM) and showed parenteral and oral efficacy (ED_50_) at less than 2.5 mg/kg doses in a *S. aureus* murine infection model. In another studies, it was demostrated that substitutions on the other three carbons generally have detrimental effect on the activity, with no clear hERG activity [53,91]. In these cases, for example, compound **290**, with a hydroxy propyl ether, retained the potency, spectrum and in vivo efficacy of **288** associated with the decreased hERG activity and improved physical property [104].

**Figure 19:**
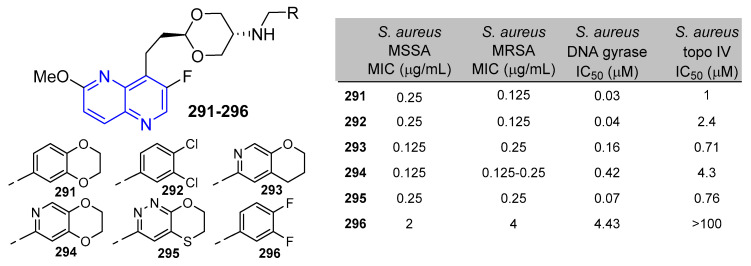
Antibacterial activity of dioxane-linked 1,5-naphthyridine derivatives.

Further studies for the development of bacterial topoisomerase inhibitors reported the synthesis of 1,5-naphthyridine dioxane-linked derivatives **291**–**296** (Figure 19) [96]. These compounds showed inhibitory activity on both gyrase and topoisomerase IV enzymes, demonstrating also improved antistaphylococcal activity and reduced hERG inhibition. Secondary amine derivatives bearing a monocyclic enzyme-binding moiety with a lipophilic *para* substituent possessed potent activity when tested against *Staphylococcus aureus*. Incorporation of a fluorinated naphthyridine DNA-binding motif robustly enhanced the antibacterial activity and topoisomerase IV inhibition of compounds **291**–**295** with minimal impact on the hERG profile. Dioxane-linked NBTIs thus offer opportunity to obtain and optimize novel therapeutics for treating antibiotic-resistant infections.

#### 5.1.3. Antiparasitic Activity

**Figure 20:**
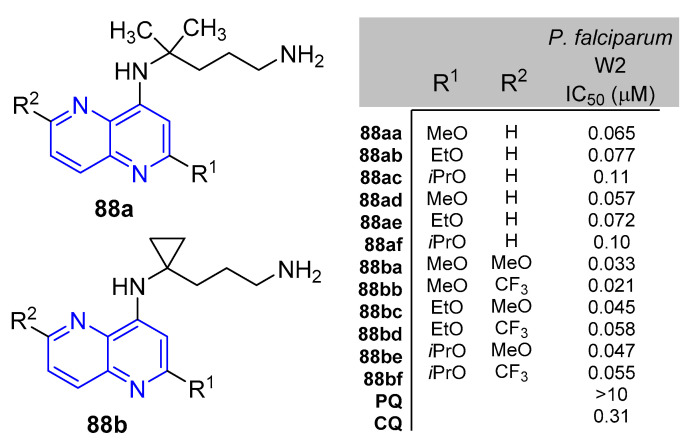
Evaluation of 1,5-naphthyridine-based molecules as antimalarial agents.

Malaria is a leading cause of mortality worldwide amongst infectious diseases. In 2007, 1,5-naphthyridine derivatives **88a** and **88b** (previously prepared, vide supra, Scheme 32) were evaluated as antimalarial agents (Figure 20) [11]. Compounds showed lower toxicity than the parent compound primaquine (PQ), preserving the desired antimalarial activity. Some compounds exhibit a therapeutic index over 10 times superior to that of the commonly used antimalarial drug chloroquine (CQ).

**Figure 21:**
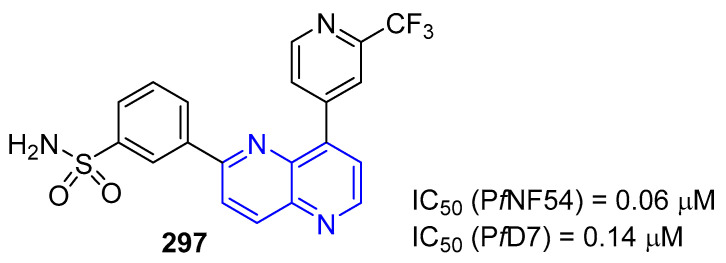
Evaluation of 1,5-naphthyridine-sulfonamides as antimalarial agents.

Later on, a series of 2,8-disubstituted-1,5-naphthyridine analogues were evaluated for in vitro antiplasmodial activity, as inhibitors of *Plasmodium* protein kinases (PKs) [35]. Several analogues exhibited low nanomolar activity against both chloroquine sensitive NF54 (IC_50_ < 100 nM) and multidrug resistant (K1) strains of the human malaria parasite *Plasmodium falciparum*. Compound **297** showed improved bioavailability in mouse and was chosen for in vivo efficacy studies based on the pharmacokinetics and antiplasmodial activity (Figure 21). From a total of 48 analogues made for SAR investigation, 26 showed improved blood stage activity compared to the original hit. Several analogues exhibited low nanomolar activity (IC_50_ < 100 nM) and were equipotent against both NF54 and K1.

**Figure 22:**
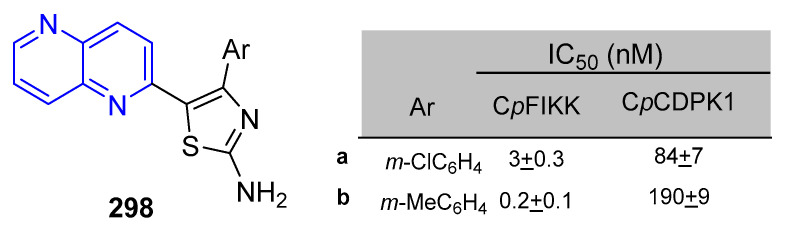
Inhibition of CpFIKK and CDPK1 kinases by a 1,5-naphthyridine aminothiazole derivative.

In order to identify inhibitors of *Cryptosporidium* FIKK (CpFIKK), a focused library of 2500 known kinases inhibitors was screened. FIKK kinases are expressed mostly during the blood stage of the *Cryptosporidium* species life cycle, as well as in the genomes of *Plasmodium*, *Toxoplasma*, *Neospora* and *Eimeria* [136]. Two compounds, with a 1,5-naphthyridine aminothiazole scaffold **298a** and **298b** showed potent inhibition of CpFIKK kinase, with IC_50_ = 3 nM and 0.2 nM, respectively (Figure 22) [101]. Moreover, compound **298b** was identified both CpCDPK1 selective as well as dually acting CpFIKK-CDPK1 inhibitor and used at appropriate concentrations, which could serve as a useful in vitro tool for understanding the biological role of FIKK in *C. parvum*.

**Figure 23:**
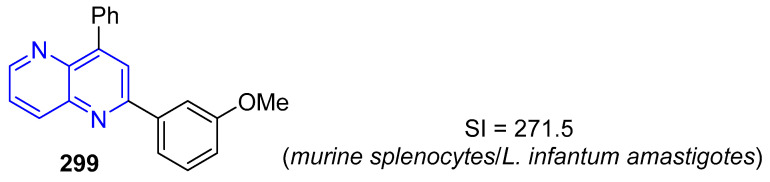
1,5-Naphthyridine derivative with antileishmanial activity.

In 2018, a wide range of substituted 1,5-naphthyridines showed antileishmanial activity on promastigotes and amastigote-infected splenocytes of *L. infantum* [46]. These 1,5-naphthyridines displayed higher antiparasitic activity on intracellular amastigotes than on free-living promastigotes. And in particular, compound **299** demonstrated its high in vitro antileishmanial activity and low toxicity showing the highest selective inhibition (SI) value of this family (SI = 271.5) (Figure 23). Moreover, these derivatives inhibited leishmanial and human topoisomerase IB enzymatic activity.

#### 5.1.4. Antiviral Activity

**Figure 24:**
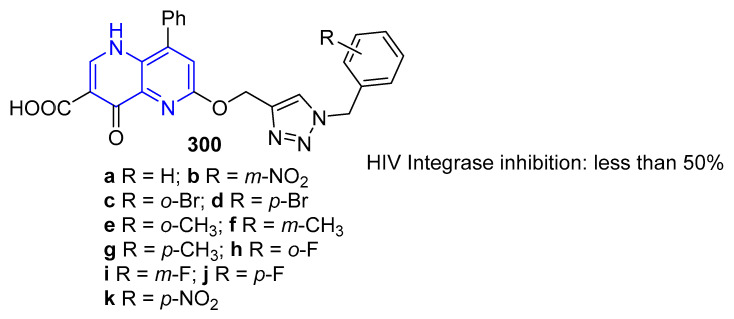
Inhibition of HIV integrase by triazole substituted 1,5-naphthyridine derivatives.

Some 1,5-naphthyridine heterocyclic derivatives are of interest as antiviral agents. In 2009 a series of triazole-substituted 1,5-naphthyridine-3-carboxylic acids **300** (Figure 24) have been screened as HIV integrase inhibitors for the treatment of AIDS, even if potential inhibitory activities were not observed [22].

**Figure 25:**
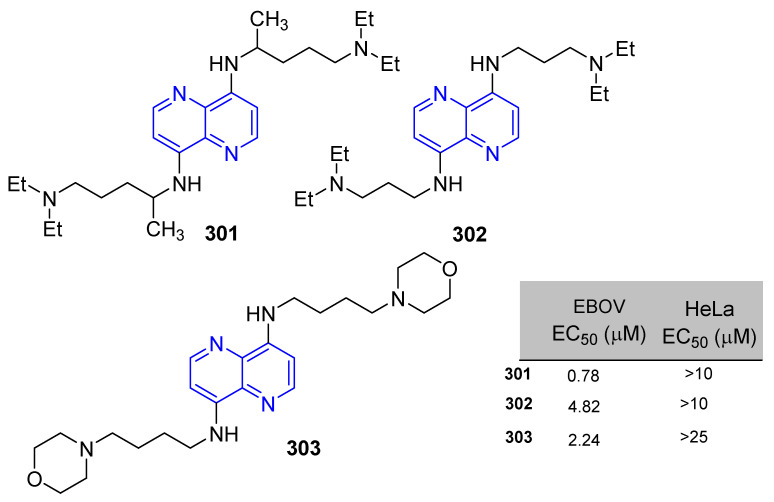
Anti-EBOV pharmacophores.

The Ebola virus (EBOV), considered a significant public health concern due its high fatality rate, can cause lethal acute hemorrhagic Ebola virus disease. There is currently no FDA-approved vaccine or medication to counter this disorder. In 2019, some aminoalkyl substituted 1,5-naphthyridines were described as anti-EBOV pharmacophores [27]. The naphthyridine inhibitors **301**, **302** and **303** were found active in low-micromolar range (Figure 25). Furthermore, the envisioned naphthyridine derivatives have a structural similarity to the FDA approved antimalarial drug CQ, which was found to have moderate anti-EBOV activity.

#### 5.1.5. Anti-inflammatory Activity

**Figure 26:**
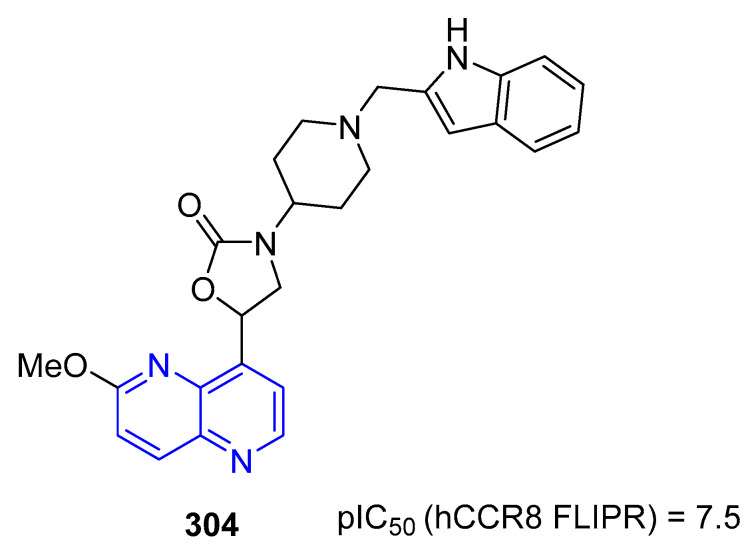
A human CCR8 antagonist. pIC_50_ value measure by a fluorometric imaging plate reader assay (FLIPR).

In 2007, some 1,5-naphthyridinyl-oxazolidin-2-one derivatives, as compound **304**, were studied as human CCR8 antagonist [137]. CCR8 may represent a potential target for treatment of asthma and other allergic diseases and it was demonstrated that CCR8 served as a co-receptor for diverse T-cell tropic, dual-tropic, and macrophage-tropic HIV-1 strains (Figure 26).

**Figure 27:**
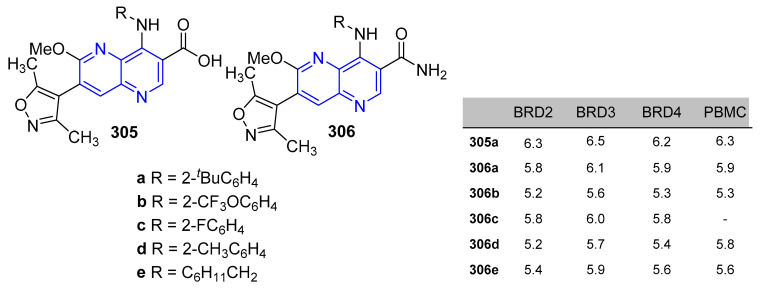
Inhibitors of bromodomain (BRD2-4) and peripheral blood mononuclear cells (PBMC) domain and their pIC_50_ values.

A series of naphthyridine derivatives **305** and **306** displayed bromodomain (BRD) and extra-terminal (BET) domain inhibition (Figure 27) [24]. The 1,5-naphthyridine derivatives with improved binding affinity relative to the 1,8- and 1,6-regioisomerswere found to be effective in a mouse model of inflammation. Cyclised analogues have good cell activity, good in vivo pharmacokinetics in rat associated with good solubility, making valuable scaffolds as bromodomain and extra-terminal (BET domain inhibitors. The best compounds were progressed in a mouse model of inflammation and exhibited dose-dependent anti-inflammatory pharmacology.

#### 5.1.6. Activity in Cardiovascular Diseases

**Figure 28:**
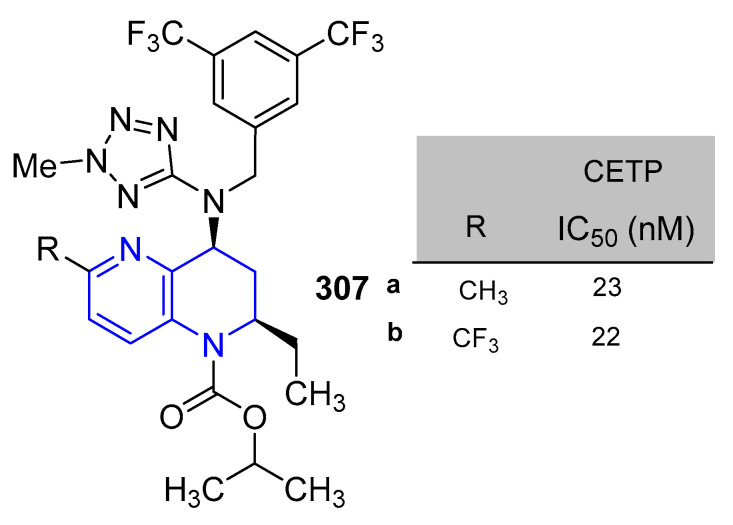
Inhibitors of CETP.

The in vitro activity as cholesterol ester transfer protein (CETP) inhibitors of some 1,5-tetrahydronaphthyridine derivatives **307**, substituted at positions 2 and 5, has been studied by Fernandez et al. (Figure 28) [47]. In this work, it was also observed that these derivatives exhibited robust HDL-c elevation in vivo. The in vitro study in human plasma led to the identification of two compounds with good activity as CETP inhibitors important for the treatment of cardiovascular diseases. 

A relationship has been observed between cardiovascular disease and hyperuricemia, hypertension, diabetes and renal disease. In 2016, in a study of a series of potent inhibitors of Human Uric Acid Transporter 1 (hURAT1), 1,5-naphthyridine was used as template. Unfortunately, the IC_50_ was quite high (>10 μM) [138].

#### 5.1.7. Activity in Central Nervous System (CNS)

**Figure 29:**
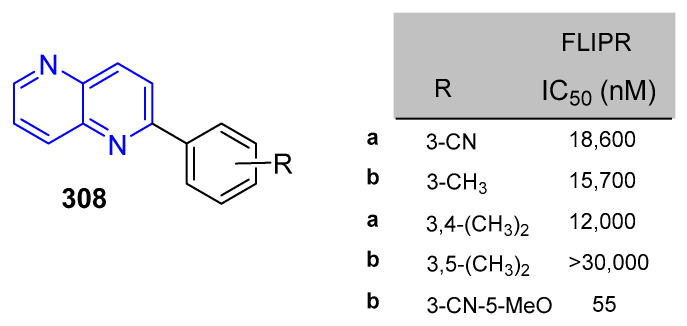
1,5-Naphthyridine analogues as mGLuR5 antagonists. pIC_50_ values measure by a fluorometric imaging plate reader assay (FLIPR).

Non-competitive metabotropic glutamate receptor 5 (mGluR5) antagonists are viewed as having promise against a number of CNS and peripheral diseases, including the treatment of pain, anxiety, gastro-esophageal reflux disease (GERD), Fragile X syndrome, and Parkinson’s disease. In this regard, 1,5-naphthyridine derivatives **308** showed activity as receptor modulators, such as mGLuR5 antagonists. (Figure 29) [94].

**Figure 30:**
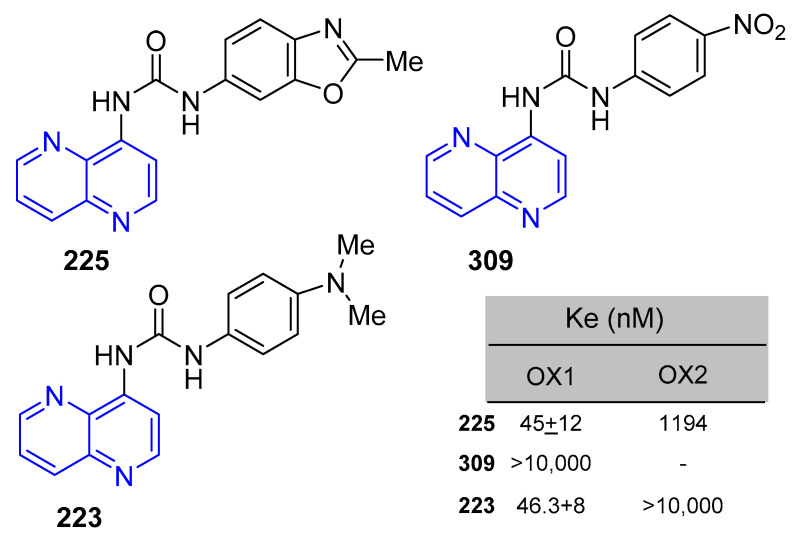
Orexin-1 receptor antagonists.

On the other hand, 1,5-naphthyridine derivatives **223** (previously prepared, vide supra, Scheme 90), **225** (previously prepared, vide supra, Scheme 91) and **309** with urea substituents were studied as orexin-1 receptor antagonists (Figure 30) [105]. Orexin-expressing neurons are located predominantly in a small area in the hypothalamus and locus cerulean. However, the nerve fibers of orexin neurons project throughout the CNS, suggesting that orexins have multiple functions. In fact, the orexin system has already been shown to modulate a variety of important biological processes, including sleep/wake cycles, feeding, drug addiction and reward, as well as energy homeostasis.

**Figure 31:**
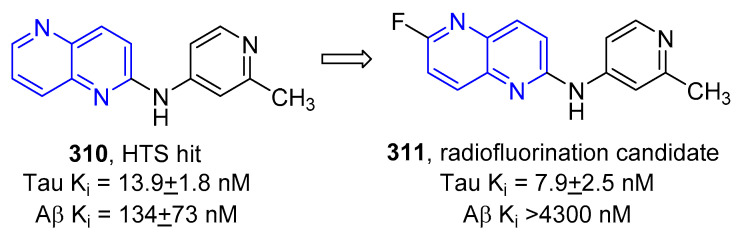
Selective tau aggregate binder.

In 2017, 1,5-naphthyridines showed an optimal pharmacokinetic profile as potent and selective binders to human Alzheimer disease (AD) aggregated tau, which implies them as potential tau PET tracer binders [38]. In certain neurodegenerative diseases, known as tauopathies, tau becomes detached from microtubules and forms insoluble aggregates in the brain. AD is characterized by plaques containing amyloid beta (Aβ) and neurofibrillary tangles (NFTs) containing tau. In a radioligand filter binding assay and via displacement studies on human AD sections, compounds **310** and **311** were shown to be potent and selective tau aggregate binder (Figure 31). Based on these results, compound **311** has been selected for [18F] radiolabeling and PET imaging studies in different preclinical species. Neither off-target activity in a receptor panel and a kinase panel, nor MAO-A and MAO-B inhibitory activities were found.

#### 5.1.8. Activity in Hormonal Diseases

**Figure 32:**
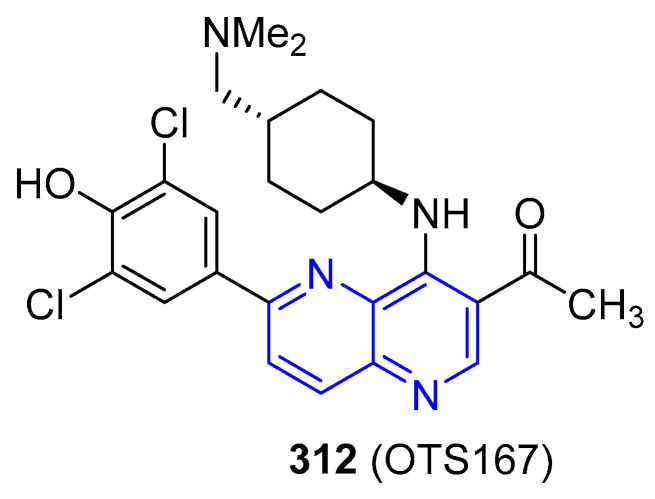
A human β-cell replication-promoter.

In a recent study, a series of 1,5-naphthyridine derivatives for the chemical inhibition of dual specificity tyrosine-phosphorylation-regulated kinase 1A (DYRK1A) have been described, as a promising therapeutic strategy for diabetes [21]. These 1,5-naphthyridine derivatives **312** (OTS167) exhibit highly potent human β-cell replication-promoting activity and dramatically reduced cytotoxicity (Figure 32). The generation of several compounds demonstrate an important, underappreciated avenue for lead series development: Desirable, highly potent “off-target” activities of advanced lead compounds.

### 5.2. Other Applications

The physical properties reported for the new 1,5-naphthyiridine derivatives include applications as sensors, semiconductors and solar cells, among others.

#### 5.2.1. Light-Emitting Compounds

It is well known that organic light-emitting diodes (OLED**s**), also known as organic EL (electroluminescent) diodes, have acquired a lot of importance since their discovery because they have high efficiency, a long lifetime and a high controllability, compared to conventional lighting devices [139]. In these OLEDs, the emissive electroluminescent layer is a film of organic compound, situated between two electrodes that emits light in response to an electric current. There are three main families of OLED materials, those based on small molecules, polymers and phosphorescent compounds.

**Figure 33:**
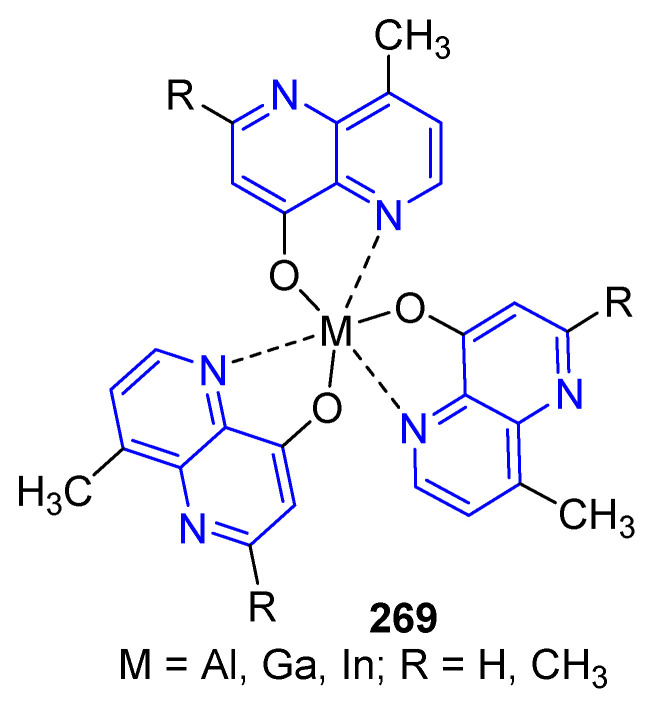
Metal complexes of group III as OLEDs.

Chelated compounds between metals of group III (Al^3+^, Ga^3+^ or In^3+^) and 4-hydroxy-1,5-naphthyridines **269** (previously prepared, vide supra, Scheme 108) have been studied as the authentic deep blue analogues of green tris-(8-hydroxyquinoline)aluminium (Figure 33). Therefore, the versatile and effective application of these chelates for OLEDs have been demonstrated [31,140]. These studies determinate the ambipolar charge transport, high carrier mobilities and high dispersive shape of the aluminium chelate derived from 1,5-naphthyridines. Their photophysical properties have been also studied by DFT and TD-DFT theoretical methods [124].

**Figure 34:**
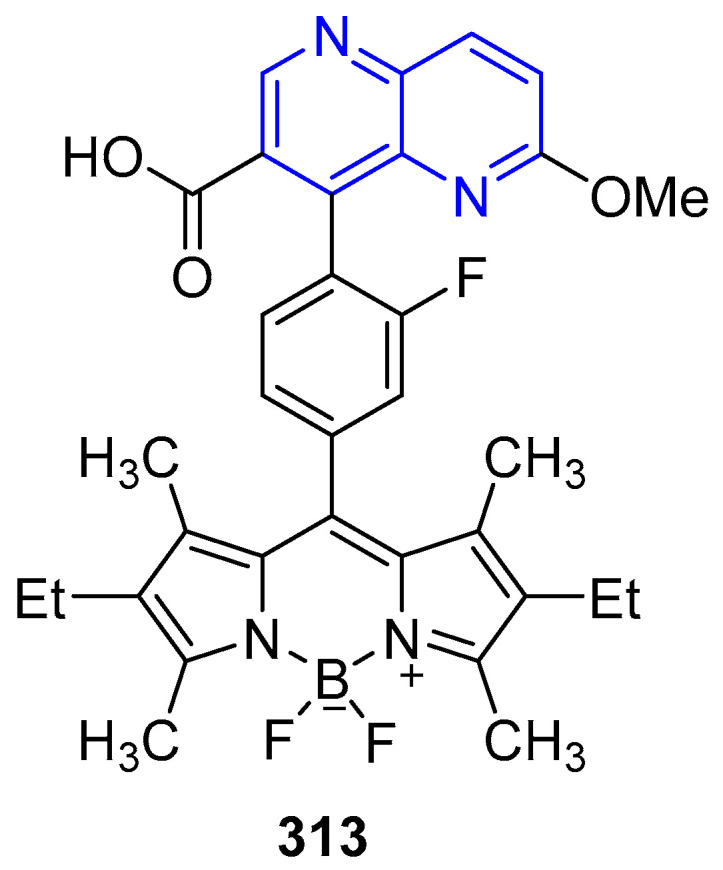
A BODIPY-linked 1,5-naphthyridine-COOH dye.

Borane naphthyridines containing carboxylic acids, such as derivative **313** (Figure 34), presented electrochemical and electrochemiluminescence properties [95,141]. Specifically, this BODIPY carboxylic acid showed a high fluorescence quantum yield, which significantly exceeds the range previously reported for structurally similar dyes. This ability results were very attractive for biological assays since the BODIPY-COOH dyes could easily conjugate to a wide range of biomolecules as it contains a carboxyl terminal. In this sense, as the dye generates significant electrochemiluminescence, these compounds serve an excellent alternative adding fluorescence for dyes with a small Stokes shift.

**Figure 35:**
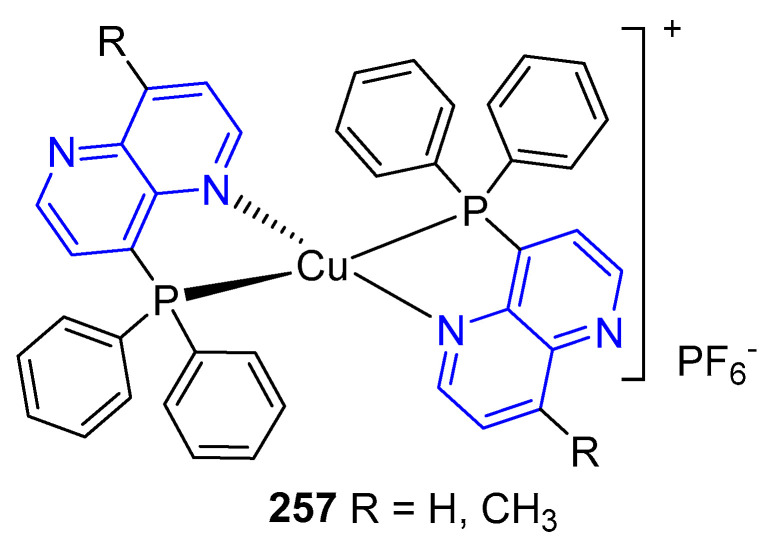
Fluorescent copper complexes.

The emission energy of copper complexes are useful for the design of emissive materials. Moreover, changes in *N*-heteroaromatic ligands vary significantly the emission energies over the visible region from red to blue and that emission energies correlated with the reduction potentials of the coordinated *N*-heteroaromatic ligands [114]. In another paper, the peak emission of copper complexes **257** (previously prepared, vide supra, Scheme 103) containing phosphino-1,5-naphthyrine ligands were shifting to the blue region compared to that of quinoline derivative (Figure 35), which could be useful in designing artifices of fluorescent complexes emitting lights that cover a full range of visible colors [115].

**Figure 36:**
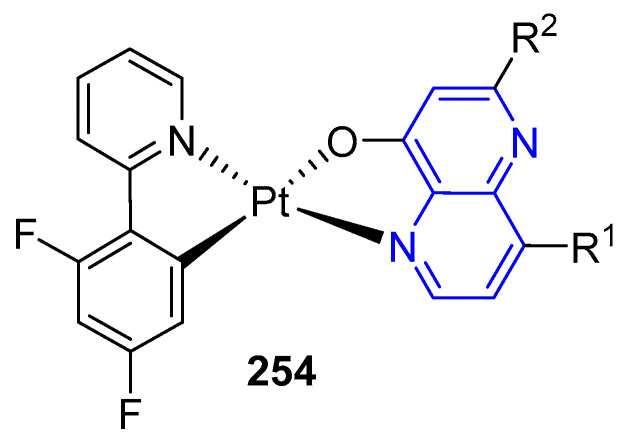
Platinum complexes as WOLEDs.

In the past few decades, white organic light-emitting diodes (WOLEDs) have drawn increasing attention because of their potential applications in full color at-panel displays and solid-state lighting. Therefore, the color of these devices could be tuned from yellow to red by adjusting the doping concentrations. For this reasons, this series of compounds can be used as emitting dyes in light-emitting diodes (LED) [19]. Some heteroleptic platinum (II) complexes with 4-hydroxy-1,5-naphthyridine ligands **254** (previously prepared, vide supra, Scheme 101) were used for the construction of WOLEDs with multilayer structures (Figure 36) [33,113,142].

**Figure 37:**
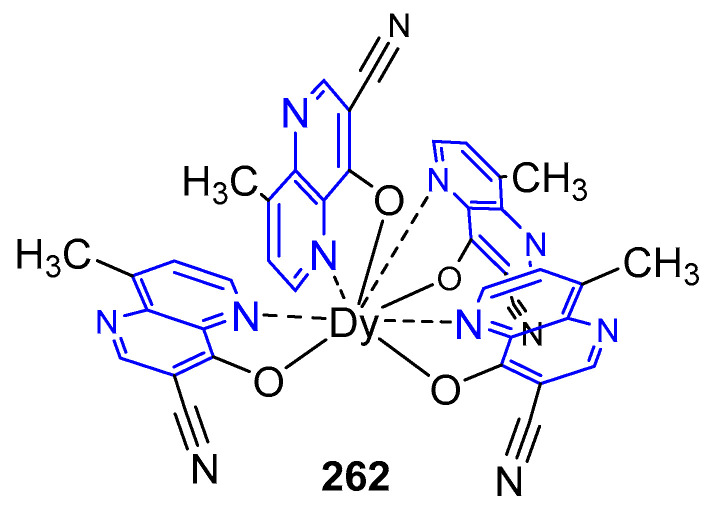
Dysprosium complexes as light emitters.

In 2016, lanthanide luminescence, based on the Dy ion, under a pulsed magnetic field for single-molecule magnets (SMMs) is observed for the first time in a series of dysprosium compound **262** (previously prepared, vide supra, Scheme 105) coordinated by four naphthyridine like ligands, containing the [DyL4] entity, and linked with an alkali metal ion (Figure 37) [122]. These SMMs will be also attractive multifunctional materials in the application of molecular spintronics and quantum computing.

**Figure 38:**
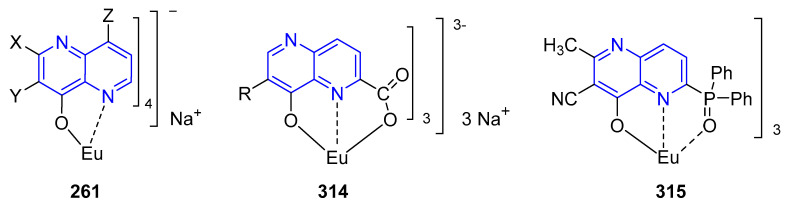
Europium complexes as light emitters.

Similarly, naphthyridine derivatives as ligands have been reported as photoluminescent when forming complexes with europium, such in **261** (previously prepared, vide supra, Scheme 105), with high photoluminescence quantum yields (PLQYs) and high thermo- and photostabilities (Figure 38) [120].

The same group, developed carboxyl functionalized naphthyridine ligands in europium(III) complexes **314** [28] or compounds with diphenylphosphoryl group **315** [29]. These compounds result quite promising for potential application in bioimaging and pH sensing, especially after the coordination stability is further improved by connecting ligands with multidentate groups such as cyclen, coating the complex with oil and nanocapsules, or dispersing the complex in SiO_2_. Comparing the bidentate complexes with the tridentate complexes, an improved and exceptionally high PLQYs in powder, as well as in a CH_2_Cl_2_ solution and poly(methylmethacrylate) films were observed.

**Figure 39:**
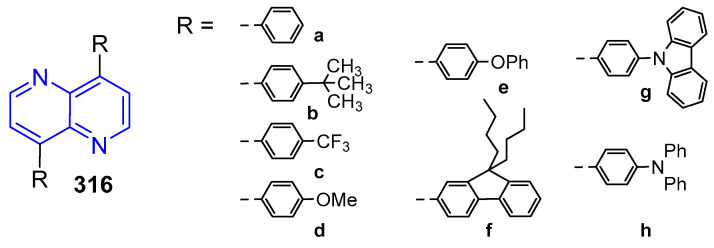
Organic light-emitting diodes.

No-metallated compounds, with a simple architecture, are attracting considerable attention in the future display technology as organic light-emitting diodes (OLEDs). In this sense, organic optoelectronic materials based exclusively on a 1,5-naphthyridine core, such as compounds **316**, were computationally studied and the quantum chemical calculations suggested that the 4,8-substituted-1,5-naphthyridines might be promising blue-emitting (or blue-green-emitting for **316h**) materials, electron transport materials and hole-injecting/hole-transport materials (Figure 39) [20].

**Figure 40:**
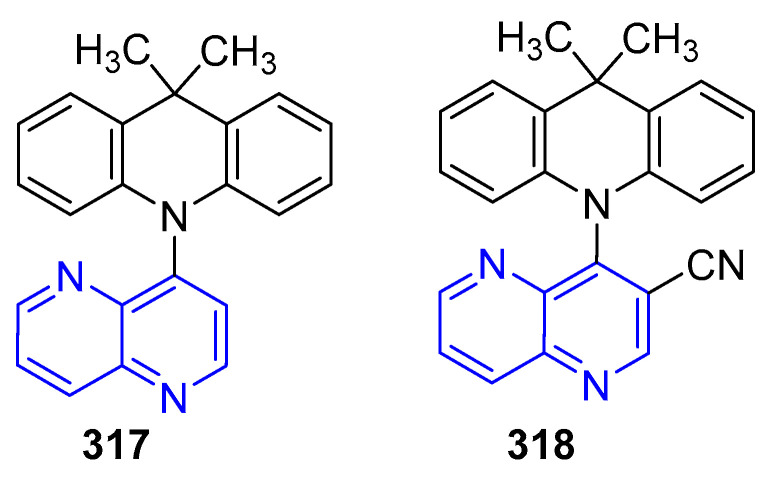
Thermally activated delayed fluorescence (TADF) emitters in OLED.

1,5-Naphthyridinylacridine-based compounds present a great potential as thermally activated delayed fluorescence (TADF) emitters in OLEDs, as indicated in a work of 2019 [66]. In this case, emitting compounds posses a suitable combination of rigid acridine donors and rigid planar naphthyridine or cyan-naphthyridine acceptors in the structures of **317** and **318** (Figure 40). Moreover, the molecule design strategy of incorporating TADF character and agrgregation induced emission (AIE) properties into one molecule provides important guidance to develop efficient neat-film emitters for high-efficiency non-doped OLEDs. Or as recently reported, 1,5-naphthyridine with acridine substituent in another position still represents a promising structure for efficient TADF materials [8].

**Figure 41:**
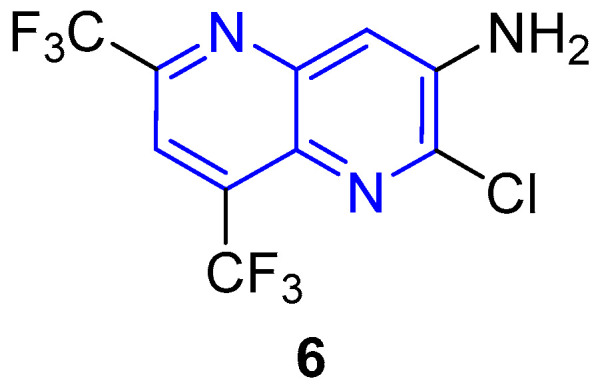
Compound with strong solvatochromic fluorescence.

Based on push–pull-type fluorophores, naphthyridine derivatives are amine sensors based on a chemical reaction between the fluorophore and the amine, and this interaction results in changes in fluorescence. Fluorophore **6** (previously prepared, vide supra, Scheme 2), having two trifluoromethyl groups and a Cl atom on a 1,5-aminonaphthyridine framework, showed strong solvatochromic fluorescence (Figure 41) [16]. In addition, in the presence of amines such as ethylamine, diethylamine, and aniline, further considerable bathochromic shifts in the fluorescence were observed. Density functional calculations identified the source of the fluorescence behavior as exciplex formation between 15-Nap-Cl and the corresponding amine. The fluorescence behavior was exploited to fabricate a sensor that can identify various primary, secondary, and tertiary amines.

#### 5.2.2. Solar Cells

Organic photovoltaics (OPVs) are particularly promising alternatives for solar-cell generation of energy because of the abundance of their constituent elements and base materials, their low cost, and relative ease of chemical synthesis. Aluminium chelates of 1,5-naphthyridine derivatives have been studied as exciton-blocking material for organic photovoltaics with prolonged lifetime [125,143]. Also π-conjugated naphthyridine N→B-ladder boranes are materials of interest for use in organic n-type applications in organic electronics [123].

**Figure 42:**
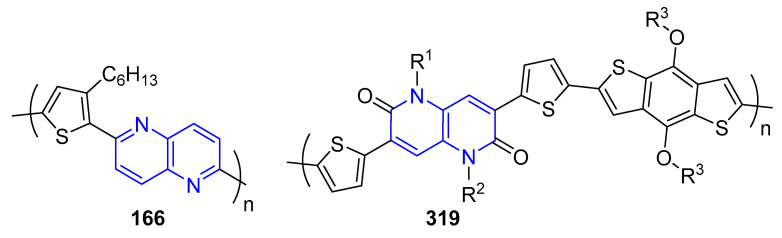
Semi-conducting polymers.

Poly[1,5-naphthyridine-(3-hexylthiophene)] semi-conducting polymer **166** (previously prepared, vide supra, Scheme 70) has been accomplished to be used as polymer-sensitized solar cell (Figure 42) [40]. In this case, solar cells are assembled using functionalized polythiophenes as a photoactive layer offering interesting properties [97]. In 2016, polymers **319** with 1,5-dialkyl-1,5-naphthyridine-2,6-dione **43** (previously prepared, vide supra, Scheme 13) as an electron acceptor and benzo[1,2-b:4,5-b′]dithiophene (BDT) as an electron donor were designed for producing efficient organic solar cells (Figure 42) [37]. Several insights of these compounds were also studied experimentally and theoretically [144].

**Figure 43:**
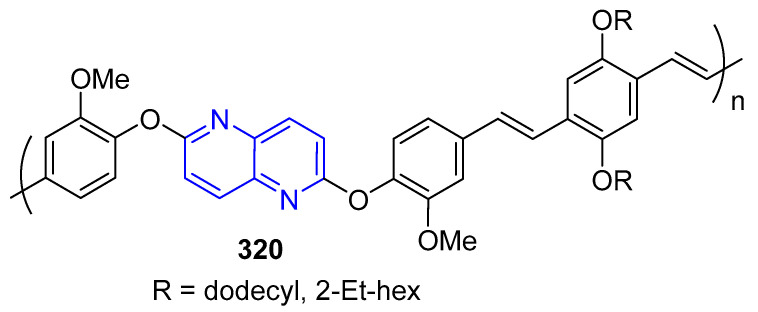
Iodide ion detectors.

Other polymeric compounds, as derivatives **320** exhibited high sensitivity and selectively in detecting iodide ion over a wide range of competing ions, such as Br^−^, Cl^−^, F^−^, NO_3_^−^, CH_3_COO^−^, SO_4_^2−^, SO_3_^2−^, S_2_O_3_^2−^ and CN^−^ (Figure 43) [67]. The incorporation of long alkoxy and alkyl side chains in the structure of these polymers rendered them better solubility in most common organic solvents, which warrant their suitability for photovoltaic devices application.

#### 5.2.3. Energy Storage

Focusing on the feasibility of using organic fuels for virtual hydrogen flow cell battery systems, based on thermodynamic considerations of fuel hydrogenation/dehydrogenation reactions, 1,5-naphthyridine derivatives have been studied as assessment of the energy density and open circuit potentials (OCPs) [145]. Heterocyclic saturated hydrocarbons and their dehydrogenation products were promising organic carriers that could yield theoretical OCPs higher than that for the hydrogen fuel cell.

**Figure 44:**
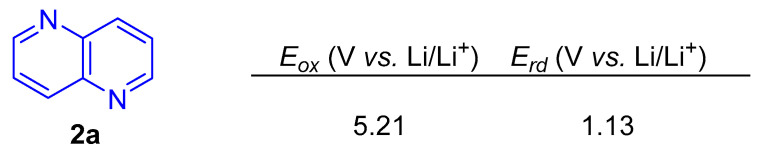
Additives in lithium-ion batteries.

In a screening by ab initio calculations, most of the suitable compounds for use as a film-forming on anodes tended to be isomers of naphthalene containing two nitrogen heteroatoms, such as 1,5-naphthyridine **2a** (previously prepared, vide supra, Scheme 1) (Figure 44) [146]. This results of interest in the search of novel functional additives and nonaqueous solvents for use in lithium-ion batteries.

**Figure 45:**
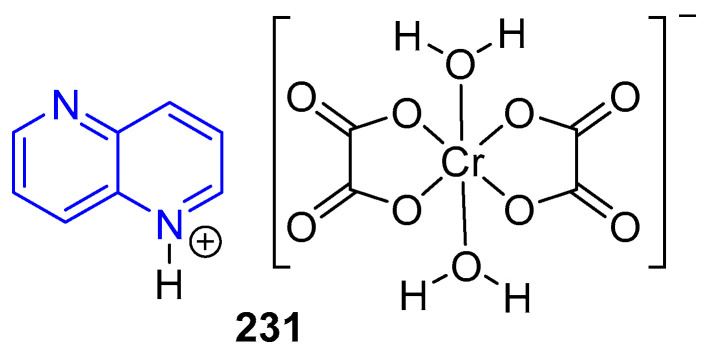
Chromium complex as semi-conductor.

A 1,5-naphthyridine *trans*-diaquadioxalatochromate (III) dihydrate complex **231** (previously prepared, vide supra, Scheme 94) was classified as semi-conductor (Figure 45) [107]. All the existing spin-allowed transitions presented in the electronic absorption spectrum has been assigned using Tanabe-Sugano diagram and the Racah parameter was calculated to evaluate the nephelauxetic effect of (Cr3þ).

## 6. Conclusions

In this review, the synthesis and reactivity of 1,5-naphthyridine derivatives published in the last 18 years have been collected. An analysis of the papers indicates that this type of compounds attracts great interest from various points of view, not only in preparative organic synthesis, but also in medicinal and applied chemistry.

In recent years, interest in their possible therapeutic properties against many diseases has increased. In fact, many of the derivatives presented in this review show important biological activity as antiproliferative, antibacterial, antifungal, antiparasitic, antiviral and anti-inflammatory, as well as activity in cardiovascular, hormonal and central nervous system diseases. In addition, a large number of works are related to other applications. Several 1,5-naphthyridines are very interesting compounds as OLEDs or for their use in solar cells.

We expect the data collected in this review, dedicated to the synthesis, reactivity and biological applications of naphthyridine derivatives, will be of interest to organic chemists, medicinal chemists and biologists. On the other hand, the data collected related to the electrical and optical properties of 1,5-naphthyridine derivatives will be useful for the development of new technologies.
